# Synthesis and preliminary evaluation of novel PET probes for GSK-3 imaging

**DOI:** 10.1038/s41598-024-65943-z

**Published:** 2024-07-10

**Authors:** Surendra Reddy Gundam, Aditya Bansal, Manasa Kethamreddy, Sujala Ghatamaneni, Val J. Lowe, Melissa E. Murray, Mukesh K. Pandey

**Affiliations:** 1 Division of Nuclear Medicine, Department of Radiology, Mayo Clinic, Rochester, MN 55905 USA; 2 Department of Neuroscience, Mayo Clinic, Jacksonville, FL 32224 USA

**Keywords:** Diagnostic markers, Drug development

## Abstract

Non-invasive imaging of GSK-3 expression in the brain will help to understand the role of GSK-3 in disease pathology and progression. Herein, we report the radiosynthesis and evaluation of two novel isonicotinamide based ^18^F labeled PET probes, [^18^F]**2** and [^18^F]**6** for noninvasive imaging of GSK3. Among the developed PET probes, the in vitro blood–brain permeability coefficient of **2** (38 ± 20 × 10^–6^ cm/s, *n* = 3) was found to be better than **6 (**8.75 ± 3.90 × 10^–6^ cm/s, *n* = 5). The reference compounds **2** and **6** showed nanomolar affinity towards GSK-3α and GSK-3β. PET probe [^18^F]**2** showed higher stability (100%) in mouse and human serums compared to [^18^F]**6** (67.01 ± 4.93%, *n* = 3) in mouse serum and 66.20 ± 6.38%, *n* = 3) in human serum at 120 min post incubation. The in vivo imaging and blocking studies were performed in wild-type mice only with [^18^F]**2** due to its observed stability. [^18^F]**2** showed a SUV of 0.92 ± 0.28 (*n* = 6) in mice brain as early as 5 min post-injection followed by gradual clearance over time.

## Introduction

Glycogen synthase kinase (GSK-3) is a constitutively active serine/threonine protein kinase with nearly 100 protein substrates. In mammals, GSK-3 is expressed as two major isoforms, GSK-3α (51 kDa) and GSK-3β (47 kDa). GSK-3α and GSK-3β show > 98% amino acid identity within their respective catalytic domains^1^. Both GSK-3α and GSK-3β are involved in the regulation of numerous biological functions such as metabolism, cell signaling, apoptosis, proliferation, differentiation and Wnt signaling pathways. These functions are considered regulatory factors in many neurodevelopmental processes^1–8^. Abnormal GSK-3α and GSK-3β activities have been associated with multiple diseases including diabetes, inflammation, cancer, bipolar disorder and neurodegenerative diseases including Parkinson’s disease (PD), Alzheimer’s disease (AD) and Huntington’s disease (HD)^9–13^. Multiple studies revealed that GSK-3α/β were overexpressed in the brains of individuals with AD and in numerous AD mouse models^14^. Tau is a phosphoprotein predominantly present in neurons with 85 potential serine (S), threonine (T), and tyrosine (Y) phosphorylation sites^2^. Aberrant GSK-3α/β levels in neurons cause hyperphosphorylation of the tau protein leading to the formation of insoluble oligomeric tau protein aggregates, known as neurofibrillary tangles (NFTs)^15,16^. Formation of NFTs in neurons disrupts their structural integrity, which is the hallmark of the AD pathology^17,18^. In a tau mouse model (rTg4510 mice), human tauopathy was replicated by overexpressing human P301L mutant tau in mouse brain and showed an age-dependent increase in neuronal loss, synaptic loss and cognitive impairment^19^. In rTg4510 mice, the accumulation of human P301L mutant tau was found to activate GSK-3β and increased the levels of phosphorylated alpha synuclein (αSyn)^19^. In fact, the interplay between insoluble tau protein, activation of GSK-3β and αSyn was hypothesized to exacerbate the pathology in rTg4510 mice^19^. Considering the importance of GSK-3 in neurological disorders, imaging of GSK-3 expression and activity will allow us to better understand the role of GSK-3 in the pathophysiology of various neurological disorders. Given the extremely high sensitivity of positron emission tomography (PET) imaging, it is an obvious choice as a technique for the visualization and quantification of GSK-3 expression in the body. Many small molecules targeting GSK-3 have been radiolabeled with ^11^C (*t*_1/2_ = 20.4 min) for GSK-3 imaging^20–37^ and significant efforts are ongoing to introduce ^18^F (t_1/2_ = 109.8 min) labeled GSK-3 radiotracers to allow relatively longer studies.

**Figure 1:**
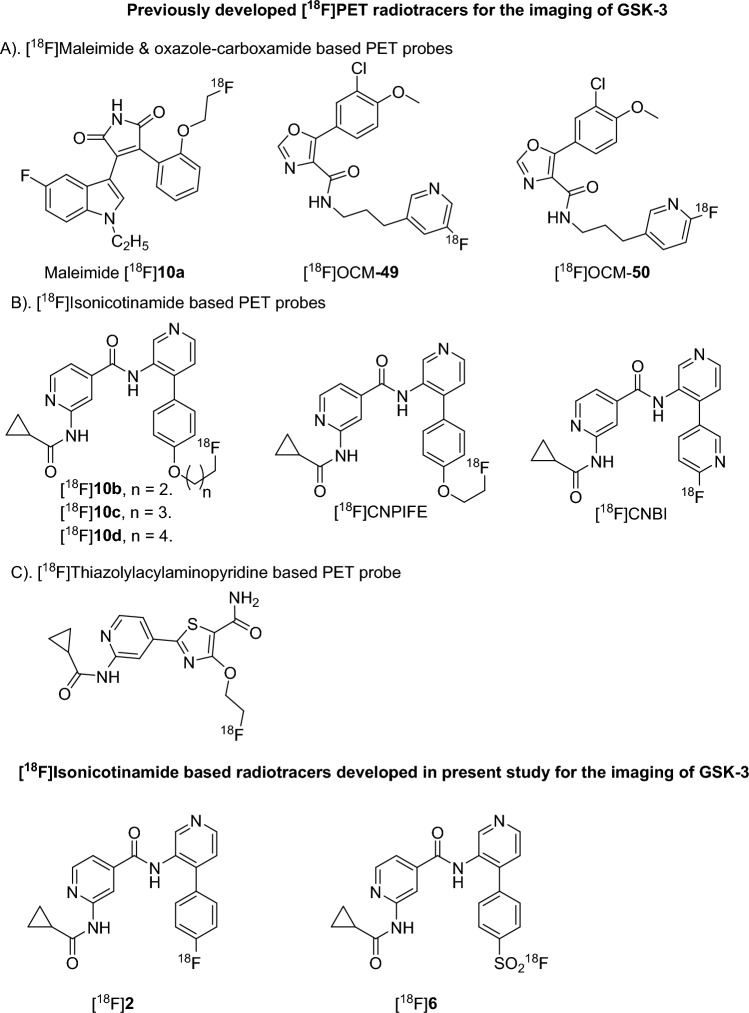
[^18^F] PET probes for GSK-3 imaging.

In 2014, Cristobal et al. studied the effect of GSK-3β overexpression on [^18^F]fluorodeoxyglucose ([^18^F]FDG) uptake by PET^38^. In 2017, Ming-Yu Ngai et al. reported maleimide based ^18^F-labeled tracers for GSK-3β imaging (Fig. 1)^39^. In 2021, Varlow et al. reported oxazole-carboxamide based ^18^F- tracers for neuroimaging of GSK-3 (Fig. 1)^40–42^. Among the ^18^F-labeled oxazole-carboxamide probes, [^18^F]OCM50 displayed good GSK-3β selectivity with a K_i_ value of 0.28 nM and a standardized uptake value (SUV) of ~ 2.0 at 2 min post injection in Sprague Dawley rats. However, [^18^F]OCM50 is yet to be investigated in a pathological model for GSK-3 imaging. Recently, Jia et al. reported thiazolyl acyl aminopyridine based ^18^F-labeled tracers for GSK-3β imaging (Fig. 1)^43^. Luo et al. in 2016 introduced an isonicotinamide class of GSK-3 inhibitors with high brain permeability and excellent GSK-3 selectivity^44^. Later, Zhong et al. and our group reported ^18^F-labeled GSK-3 PET imaging probes with an isonicotinamide core. An ^18^F-isonicotinamide PET probe with bipyridine group ([^18^F]CNBI) was synthesized via aromatic nucleophilic radio fluorination (S_N_Ar) using ^18^F^–^ (fluoride). However, the [^18^F]CNBI PET probe showed moderate metabolic stability and poor brain uptake in FVB/NJ mice (Fig. 1). The ^18^F- labeled isonicotinamide PET probes containing a phenyl pyridine group with alkoxy group substitution ([^18^F]CNPIFE & [^18^F]**10b-d**) were synthesized by aliphatic nucleophilic radio fluorination (S_N_2) using ^18^F^-^ (fluoride)^45–47^. These alkoxy substituted ^18^F probes showed moderate blood–brain barrier permeability and good selectivity towards GSK-3. Unfortunately, these probes were found to be metabolically unstable in serum. To continue our endeavors on developing a better PET probe for GSK-3 imaging, we further designed two new isonicotinamide based PET probes, [^18^F]**2** and [^18^F]**6** (Fig. 1). The developed PET probe [^18^F]**2** is designed based on the highly potent, selective, and orally active GSK-3 inhibitor that was presented in Luo et al.’s study^44^. On the other hand, [^18^F]**6** was designed by replacing the fluorine atom of [^18^F]**2** with a fluoro-sulfate group for fast isotopic exchange with ^18^F-fluoride.

The present study describes the radiosynthesis of [^18^F]**2** and [^18^F]**6** along with the synthesis of their reference standards (**2** and **6**) and precursors. Their preliminary evaluation is also detailed, including blood–brain barrier permeability, affinity towards GSK-3α/β and radiochemical stability in isotonic saline and mouse and human serums. PET probe [^18^F]**2** was also evaluated for in vivo PET imaging and ex vivo biodistribution with and without self-blocking in wild type FVB/NJ mice.

## Results and discussion

### Synthesis of precursors and reference standards

**Figure 2:**
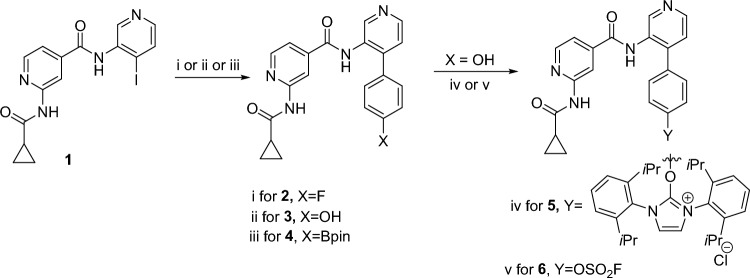
Synthesis of reference standards and precursors. Reaction condition: (i) Pd(PPh_3_)_4_ (0.3 equiv.), K_2_CO_3_ (3.0 equiv), 4-flourophenyl boronic acid pinacol ester (1.2 equiv.), 80 °C, Dioxane:H_2_O (4:1), 8.0 h. 65%. (ii) Pd(PPh_3_)_4_ (0.3 equiv.), K_2_CO_3_ (3.0 equiv.), 4-hydroxyphenyl boronic acid pinacol ester (1.2 equiv.), 80 °C, Dioxane:H_2_O (4:1), 8.0 h, 50%. (iii) Pd(PPh_3_)_4_ (0.1 equiv.), K_2_CO_3_ (3.0 equiv.), 1,4-benzenediboronic acid dipinacol ester (3.0 equiv.), 80 °C, Dioxane:H_2_O:EtOH (4:1:1), 8.0 h, 10%. (iv) Ag_2_CO_3_ (0.5 equiv.), 2-chloro-1,3-bis(2,6-diisopropylphenyl)imidazolium chloride (1.0 equiv.), CHCl_3_, 60 °C, 4.5 h. 60% (v) 1-(Fluorosulfuryl)-2,3-dimethyl-1*H*-imidazol-3-ium trifluoromethanesulfonate (1.2 equiv.), Et_3_N (1.5 equiv.), CH_3_CN, room temperature (RT), 1.0 h. 50%.

The synthetic routes of the key intermediates, precursors and non-radioactive standards are presented in Fig. 2. Briefly, the synthesis was initiated by preparing 2-(cyclopropanecarboxamido)*-N*-(4-iodopyridin-3-yl)isonicotinamide (**1)** by following a previously well-established method^44–47^. The ^1^H and ^13^C-NMR spectroscopic data were consistent with previously reported values. The reference compound **2** and intermediate **3** were prepared by palladium (Pd) catalyzed Suzuki–Miyaura coupling of 2-(cyclopropanecarboxamido)-*N*-(4-iodopyridin-3-yl)isonicotinamide (**1**) with 4-flourophenyl boronic acid pinacol ester and 4-hydroxyphenyl boronic acid pinacol ester, respectively. Compound** 4**, the boronic ester precursor for radiolabeling with fluorine-18, was synthesized using the above mentioned Suzuki–Miyaura coupling with slight modification including; reducing the Pd-catalyst quantity from 0.3 equivalent to 0.1 equivalent and increasing the amount of 1,4-phenyldiboronic ester from 1.2 equivalent to 3.0 equivalent with 2-(cyclopropanecarboxamido)-*N*-(4-iodopyridin-3-yl)isonicotinamide (**1**). Compound **5**, the uronium precursor for radiolabeling with fluorine-18, was prepared by treating intermediate **3** with Ag_2_CO_3_ and 2-chloro-1,3-bis(2,6-diisopropylphenyl)imidazolium chloride in chloroform at 60 °C. Compound **6**, the fluoride-18 exchange precursor, was obtained by treating intermediate **3** with 1-(fluorosulfuryl)-2,3-dimethyl-1*H*-imidazol-3-ium trifluoromethanesulfonate and triethyl amine in acetonitrile. All synthesized compounds were fully characterized by standard spectroscopic techniques including ^1^H-NMR, ^13^C-NMR, ^19^F-NMR, IR and HRMS (NMR and HRMS spectral data are included in supporting information).

### Radiochemistry

**Figure 3:**
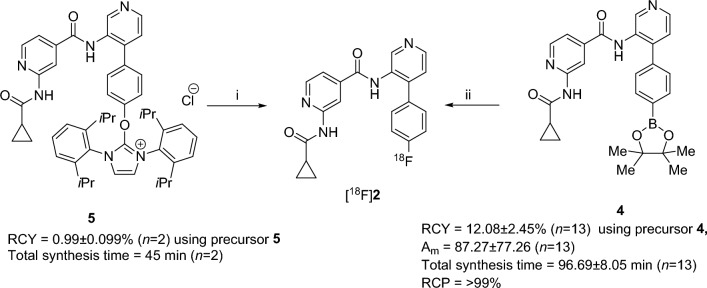
Radiosynthesis of [^18^F]**2**. Reaction conditions: (i) fluoride-18, 2-butanone: EtOH: NBu_3_ = 10:1:0.1 (1.0 mL), 130 °C, 20 min. 0.99 ± 0.099% (*n* = 2) decay-corrected radiochemical yield (RCY), total synthesis time = 45 min (*n* = 2). (ii) Kryptofix 222, K_2_CO_3_, fluoride-18, 120 °C, DMI (300 µL), Cu(OTf)_2_(impy)_4_ (2.0 Equai.) 20 min. 12.08 ± 2.45% (*n* = 13)) decay-corrected radiochemical yield (RCY), molar activity (A_m_) of 87.27 ± 77.26 (*n* = 13) GBq/µmole, total synthesis time = 96.69 ± 8.05 min (*n* = 13).

**Figure 4:**
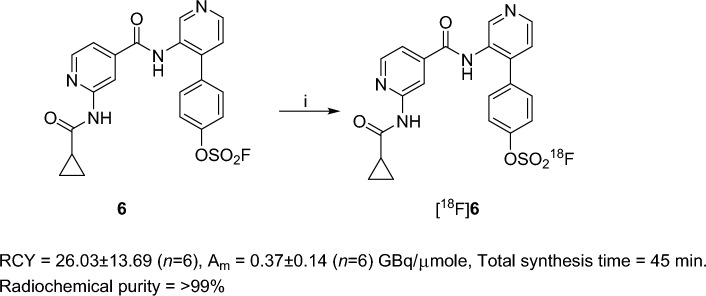
Radiosynthesis of [^18^F]**6**. Reaction condition: i) Kryptofix 222, fluoride-18, K_2_CO_3_, RT, 5 min, CH_3_CN, 26.03 ± 13.69% (*n* = 6) decay-corrected radiochemical yield (RCY), A_m_ of 0.37 ± 0.14 (*n* = 6) GBq/µmole at end of the formulation. Total synthesis time = 45 min.

The radiosynthetic route for [^18^F]**2** and [^18^F]**6** is outlined in Figs. 3 and 4, respectively. For the radiosynthesis of [^18^F]**2,** initially, we employed the ^18^F-deoxyfluorination method on compound **5**, as reported by Neumann et al.^48^ This method offers several advantages over other methods including a stable and easily accessible precursor, no azeotropic drying for fluorine-18 and faster radiolabeling, but it gave 0.99 ± 0.09% (*n* = 2) decay-corrected radiochemical yield. Therefore, we selected another well-established copper mediated oxidative ^18^F-flourination approach using aryl boronic pinacol ester, which typically tolerates both electron rich and poor arenes having multiple functional groups. In this method, we employed copper-mediated oxidative ^18^F-flourination on compound **4** as reported by Chen et al. with azeotropically dried fluoride-18 in 1,3-dimethyl-2-imidazolidinone (DMI) as a solvent at 120 °C for 20 min^49^. This method gave 12.08 ± 2.45% (*n* = 13) decay-corrected radiochemical yield and a molar activity (A_m_) of 87.27 ± 77.26 (*n* = 13) GBq/µmole of [^18^F]**2** with > 99% radiochemical purity (SI-Fig. S2). For radiosynthesis of [^18^F]**6,** we opted for ^18^F-SuFEx click chemistry as reported by Peng Wu et al.^50^. In this method, we treated compound **6** with azeotropically dried fluoride-18 at room temperature (RT) for 5 min in acetonitrile as a solvent. This method gave 26.03 ± 13.69% (*n* = 6) decay-corrected radiochemical yield with a molar activity (A_m_) of 0.37 ± 0.14 (*n* = 6) GBq/µmole of [^18^F]**6** at > 99% radiochemical purity (SI-Fig. S5).

### Blood–brain barrier permeability, binding affinity, and serum stability assessments

To evaluate the blood–brain barrier permeability and binding affinity of the developed PET probes[^18^F]**2** and [^18^F]**6**, their respective nonradioactive versions were used. For blood–brain barrier permeability evaluation, we applied in vitro parallel artificial membrane permeability assay (PAMPA) and evaluated binding affinity via an inhibition assay to determine the IC_50_ value, as described in method section. We further measured the stability of both PET probes, [^18^F]**2** and [^18^F]**6**, using radio thin layer chromatography (radio-TLC) over time, as also described in the method section. The IC_50_ values of **2** and **6** towards GSK-3α and GSK-3β were found to be in nanomolar range as outlined in Table 1 and SI-Fig. S16. Among the two compounds, **2** showed ~ 1.8-fold higher affinity towards both GSK-3α and GSK-3β as compared to **6**.

**Table 1:** IC_50_ values of **2** and **6** against glycogen synthase kinase α and β (GSK-3α and β).

Compound	IC_50_ GSK-3α (nM)	IC_50_ GSK-3β (nM)
2	29.24 ± 2.93 (*n* = 3)	24.29 ± 2.67 (*n* = 3)
6	55.66 ± 4.89 (*n* = 3)	43.99 ± 2.84 (*n* = 3)

Blood–brain barrier permeability results for both non-radioactive compounds **2** and **6** are shown in Table 2. In this PAMPA assay, two controls (propranolol HCl and atenolol) were used. Propranolol is highly blood–brain permeable and showed a permeability coefficient of 32 ± 1 × 10^–6^ cm/s (*n* = 4), whereas atenolol has low blood–brain permeability and showed a permeability coefficient of < 0.14 × 10^–6^ cm/s (*n* = 3). Among the tested compounds, **2** showed a permeability coefficient of 36 ± 20 × 10^–6^ cm/s (*n* = 3) and -logP_e_ value of 4.50 ± 0.24 (*n* = 3), whereas **6** showed a permeability coefficient of 8.75 ± 3.90 × 10^–6^ cm/s (*n* = 5) and − logP_e_ value of 5.10 ± 0.19 (*n* = 3). It was encouraging to observe that both **2** and **6** showed greater permeability than the low blood–brain permeable atenolol. Interestingly, **2** expressed a much closer permeability coefficient to the highly blood–brain permeable control propranolol HCl, as compared to **6**. The blood–brain barrier permeability parameters of **2** were even better than those reported previously for other isonicotinamide-based candidates, [^19^F]F-CNBI (1.6 ± 0.01 × 10^–6^ cm/s (*n* = 3) and [^19^F]F-CNPIFE (14.29 ± 0.25 × 10^–6^ cm/s (*n* = 5) (Table 2)^46^. The affinity of the developed compounds, **2** and **6** were measured against GSK-3α and β through the IC_50_ value and are shown in Table 1. Both the compounds showed nanomolar affinity, but compound **2** showed stronger inhibition to both GSK-3α/β as compared to compound** 6** in an inhibitory assay.

**Table 2:** Blood–brain barrier permeability results for compounds **2** and **6** at pH 7.4.

Compound	Permeability coefficient, P (10^–6^ cm/s), Average ± SD	− logP_e_, Average ± SD
**2**	38 ± 20 (*n* = 3)	4.50 ± 0.24 (*n* = 3)
**6**	8.75 ± 3.90 (*n* = 5)	5.10 ± 0.19 (*n* = 5)
Propranolol HCl (highly permeable control)	32 ± 1 (*n* = 3)	4.50 ± 0.01 (*n* = 3)
Atenolol (low permeable control)	< 0.14 (*n* = 3)	–

**Figure 5:**
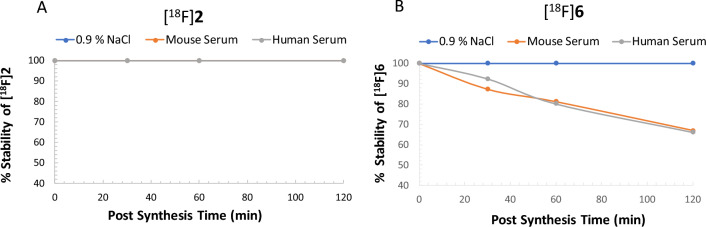
Scatter plot showing the stability of (**A**) [^18^F]**2** and (**B**) [^18^F]**6** in isotonic NaCl solution and mouse and human serums over time.

The stability of PET probes [^18^F]**2** and [^18^F]**6** was measured in isotonic NaCl solution (0.9% NaCl) and mouse and human serums at 37 °C for up to 120 min by rad-TLC at 0 min, 30 min, 60 min and 120 min post incubation. The results obtained are presented in Fig. 5 and SI-Figs. S10–S15, SI-Table S1. Of the two PET probes, [^18^F]**6** showed poor stability in both mouse and human serums as compared to [^18^F]**2**. In the case of PET probe [^18^F]**2**, no degradation observed up to 120 min in isotonic NaCl solution or human and mouse serums. On the other hand, [^18^F]**6** showed defluorination from 30 min time point onwards in both mouse and human serums. In isotonic NaCl solution, [^18^F]**6** showed complete stability up to 120 min. In mouse serum, no degradation was observed at 0 min incubation, but [^18^F]**6** showed only 87.30 ± 8.99% stability at 30 min, which was further decreased to 67.01 ± 6.38% (*n* = 3) at 120 min post incubation. Similarly, in human serum no degradation observed at 0 min incubation, but, [^18^F]**6** showed only 92.46 ± 2.6% stability at 30 min, which was further decreased to 66.20 ± 4.93% (*n* = 3) at 120 min post incubation. The plausible explanation of the instability of [^18^F]**6** could be attributed to the covalent interaction of [^18^F]**6**’s SO_2_F group with primary amines of amino acid residues present in the protein rich mouse and human serums. In fact, in an unrelated study, Zhao et al. reported formation of sulfonamide bond between sulfuryl fluoride and primary amine of conserved lysine residues of different kinases^51^. In contrast, PET probe [^18^F]**2** showed high stability in isotonic saline, mouse and human serums as compared to previously reported isonicotinamide based ^18^F-PET probes^45,46^. Based on **2**’s favorable blood–brain barrier permeability, nanomolar binding affinity and PET probe [^18^F]**2**’s excellent stability in both mouse and human serums, further in vivo evaluations were performed only with PET probe [^18^F]**2**.

### PET imaging and ex-vivo biodistribution

**Figure 6:**
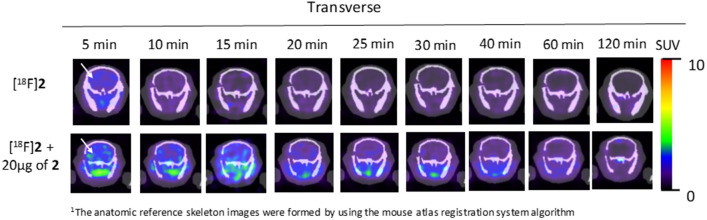
Representative PET/X-ray^1^ images (transverse section) showing uptake of [^18^F]**2** with 20 µg of **2** in brain (white arrow) of wild-type FVB/NJ mice at different time points post-injection.

**Figure 7:**
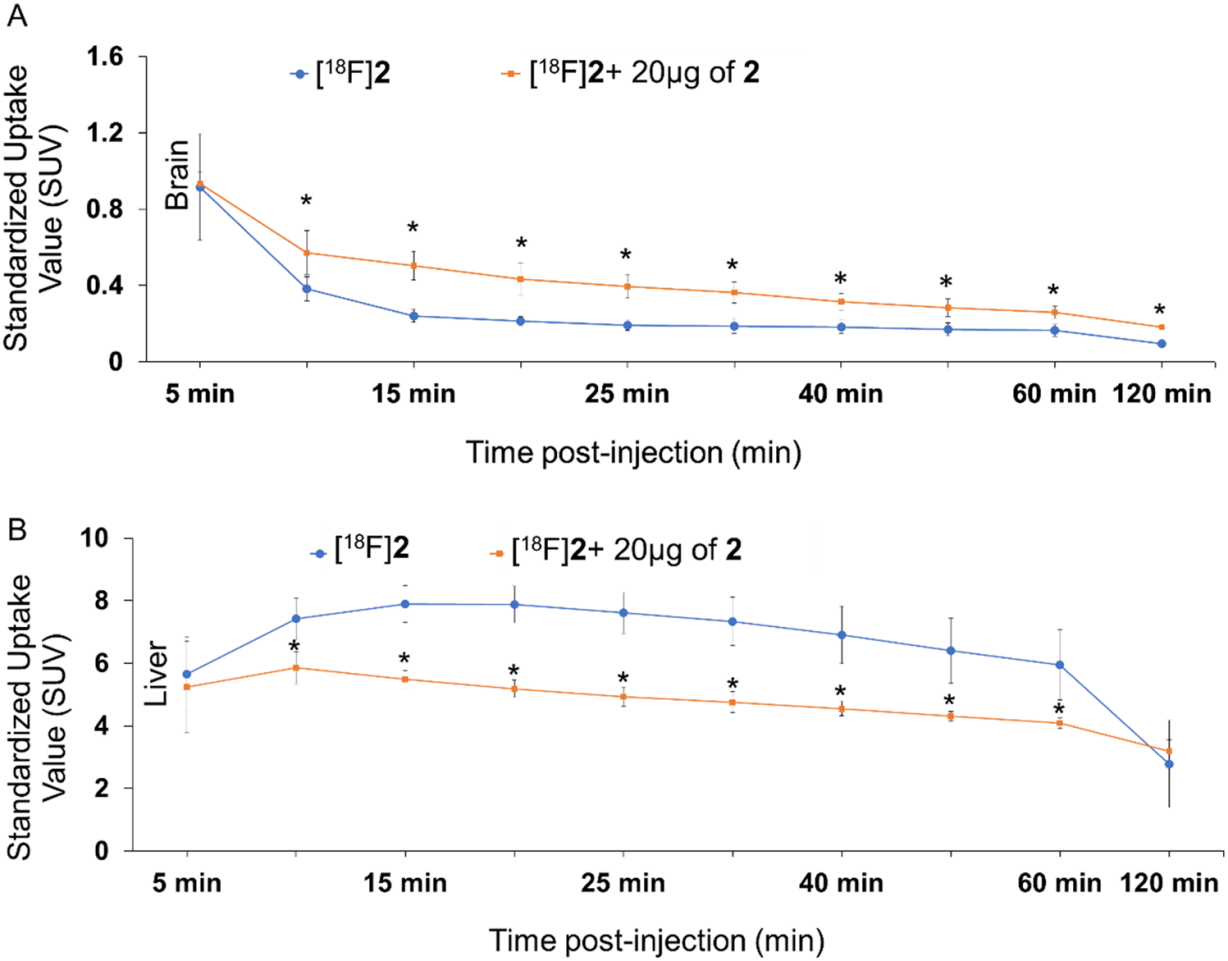
Time-activity curves of [^18^F]**2** at the baseline (blue) and under blocking conditions [^18^F]**2** with 20 µg of **2** (orange) in the (**A**) brain and (**B**) liver of wild-type FVB/NJ mice at different time points (min) post-injection. Data expressed as standardized uptake value (SUV). The SUVs were calculated by PET image analysis and each data point is average ± standard deviation. *P < 0.05 [^18^F]**2** vs [^18^F]**2** + 20 µg of **2**.

**Figure 8:**
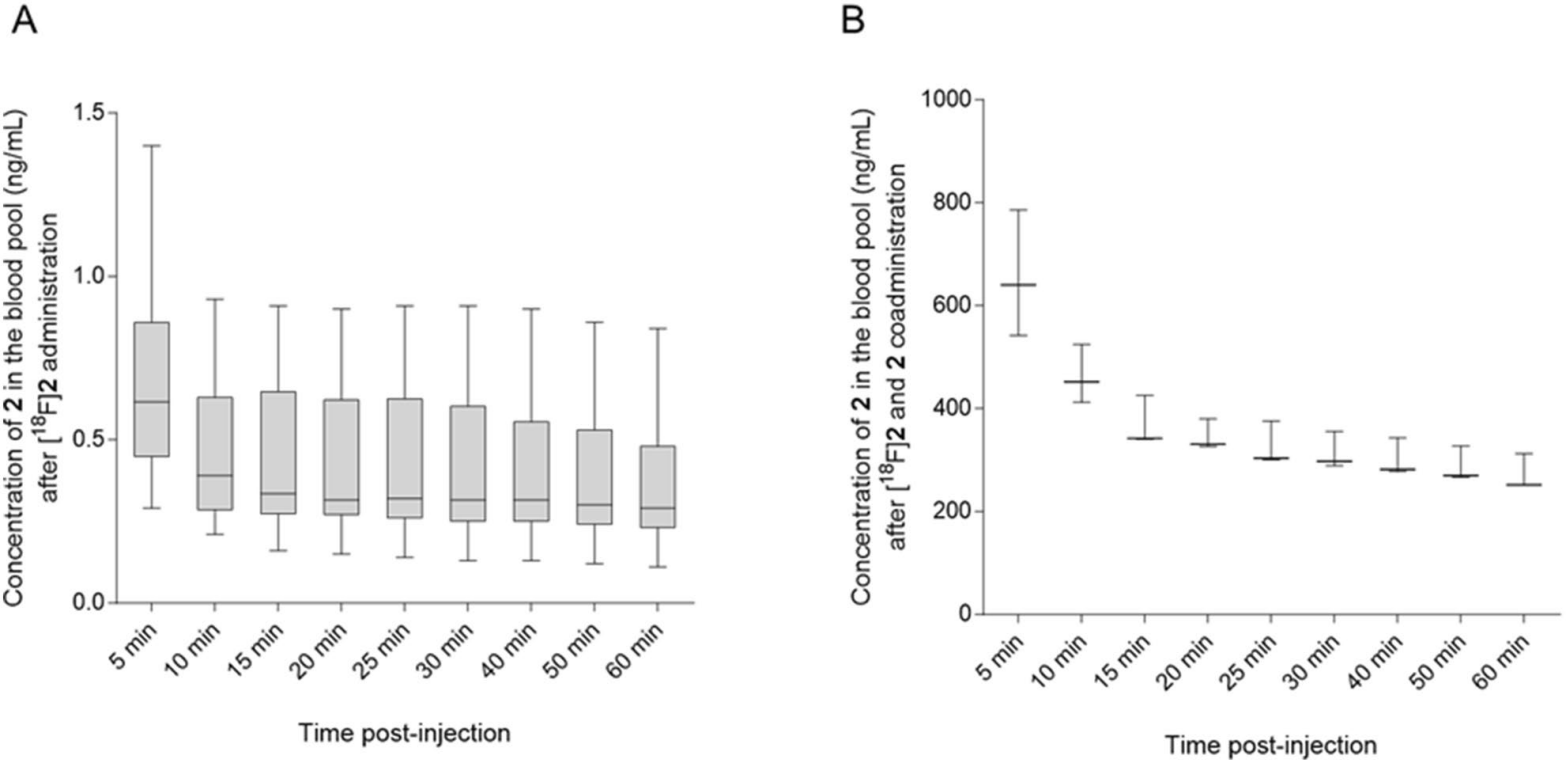
Box and Whiskers plot showing blood clearance of compound **2** overtime (**A**) after administration of [^18^F]**2** (*n* = 6) and (**B**) after coadministration of [^18^F]**2** with 20 µg of **2** (*n* = 3) in FVB/NJ mice.

To evaluate the imaging potential and brain uptake of PET probe [^18^F]**2,** we selected FVB/NJ wild-type mice and performed radiotracer uptake studies via tail vein injection of [^18^F]**2** followed by dynamic imaging upto 60 min and static imaging at 120 min post-injection. Ex-vivo biodistribution was performed immediately after 120 min static imaging. Uptake of [^18^F]**2** was assessed in the brain and liver of wild-type FVB/NJ mice by PET imaging at different time points post-injection with and without co-administration of **2** (reference compound as a self-blocking agent). Representative transverse sectional PET images of the brain at different time points are shown in Fig. 6 and representative coronal and sagittal sectional PET images of the liver and whole-body PET images are shown in SI-Figs. S17 and S18, respectively. It was encouraging to observe brain uptake of PET probe [^18^F]**2** with a standard uptake value (SUV) of 0.92 ± 0.28 (*n* = 6) at 5 min post-injection. Approximately 80% of PET probe [^18^F]**2** was cleared from the wild-type mice brain in the first 30 min. As expected, we observed a significantly different uptake profile for [^18^F]**2** in the brain and liver as summarized in Fig. 7 and SI-Table S2. The uptake of [^18^F]**2** in the brain was lower than in the liver at all time points post-injection. In addition, co-administration of compound **2** with PET probe [^18^F]**2**, showed no difference in uptake in the brain at the first 5 min time point, but increased to 1.5-fold at 10 min time point and again to twofold at 15 min, 20 min, 25 min and 30 min time points, indicating a systemic response. Interestingly, while the coadministration of compound** 2** increased uptake of [^18^F]**2** in the brain, uptake in the liver simultaneously decreased. In fact, co-administration of nonradioactive reference compound (blocking with** 2**) should decrease the uptake of [^18^F]**2** as observed in liver, but in contradiction, it increased the uptake of [^18^F]**2** in the brain, further suggesting a systemic response. The contrasting response in uptake of [^18^F]**2** in the brain in the presence of **2** could be attributed to the peripheral effect. It is plausible that co-administration of **2** would preferably bind to GSK-3α/β present in the liver and would decrease the overall uptake of [^18^F]**2** in the liver but would simultaneously enhance the bioavailability of [^18^F]**2** to the brain and other highly perfused organs, thereby enhancing the uptake in those organs. This is also evident from the higher concentration of **2** in the blood of the animals in which [^18^F]**2** was co-administered with **2** as compared to administration of only [^18^F]**2** (Figs. 6, 7 and 8 and SI-Table S2, SI-Figs. S17, S18). A similar observation was also reported by Lindberg et al. 2021 with [^11^C]verubulin in rats, where co-injection of verubulin increased the uptake of [^11^C]verubulin in the brain and was attributed to the saturation of the enzymes (e.g. cytochrome P450 enzymes) that metabolize the tracer in the liver, leading to increased bioavailability^52^. It is also plausible that the isonicotinamide class of compounds are substrate for cytochrome P450 enzymes.

To further understand the pharmacokinetics of [^18^F]**2**, ex vivo biodistribution was performed with different groups of mice with and without co-administration of **2**. Animals were sacrificed at 5 min, 10 min and 120 min post-injection of [^18^F]**2**. Vital organs and tissues were collected and uptake of the [^18^F]**2** was measured as SUV after counting the radioactivity in each harvested tissue. A significant increase in the uptake of [^18^F]**2** in cerebellum, brain stem, thalamus, hippocampus, cortex, and caudate nucleus was observed in the presence of **2** at 120 min post-injection as summarized in Table 3 and SI-Figs. S19–S21. Based on ex vivo biodistribution, increased radioactivity in the blood in the presence of **2** at 5 min, 10 min and 120 min post-injection supports the hypothesis of increased bioavailability of [^18^F]**2** in the presence of **2**. Other than in the brain, the positive effect of **2** on uptake of [^18^F]**2** was also observed in the lungs, heart, kidneys, muscle, small intestine, cecum, bladder, and feces collected at 5 min and 10 min post-injection as shown in Table 3, SI-Figs. S19 and S20. Other organs and tissues like spleen, pancreas and adipose tissue showed higher uptake of [^18^F]**2** in the presence of **2** at 120 min post-injection also supporting the increase in bioavailability of [^18^F]**2** as shown in Table 3 and SI-Fig. S21. Additionally, observed higher uptake of [^18^F]**2** in pancreas could potentially be extended to beta cell mass imaging as a separate study. Presence of higher quantity (radioactivity) of [^18^F]**2** in urine, cecum, and feces at 120 min post-injection as compared to 5 min and 10 min post-injection shows increased excretion of the radiotracer with time. Even though PET probe [^18^F]**2** shows moderate brain uptake in wild-type mouse brain, it would be intriguing to know how selective this probe would be in a GSK-3 over-expressing mouse brain. The PET probe [^18^F]**2** belongs to the isonicotinamide class of compounds, which were developed as highly selective inhibitors for GSK-3, among other kinases^44^. Therefore, it would be valuable to evaluate PET [^18^F]**2** in pathological conditions to finally evaluate its selectivity, binding affinity, and retention in vivo in the presence and absence of self-blocking as a future study.

**Table 3:** Uptake (SUV) and biodistribution of [^18^F]**2** and [^18^F]**2** with 20 µg of **2** in whole body of wild-type FVB/NJ mice at 5 min, 10 min and 120 min post intravenous (i.v.) administration assessed by ex vivo biodistribution.

Organ	[^18^F]**2**	[^18^F]**2** + 20 µg of **2**	[^18^F]**2**	**[**^18^F]**2** + 20 µg of **2**	[^18^F]**2**	[^18^F]**2 + ** 20 µg of** 2**
	5 min post-injection	5 min post-injection	10 min post-injection	10 min post-injection	120 min post-injection	120 min post-injection
	SUV, Average ± SD (*n* = 4)	SUV, Average ± SD (*n* = 3)	SUV, Average ± SD (*n* = 4)	SUV, Average ± SD (*n* = 3)	SUV, Average ± SD(*n* = 3)	SUV, Average ± SD (*n* = 3)
Cerebellum	0.75 ± 0.49	0.62 ± 0.03	0.30 ± 0.05	0.39 ± 0.15	0.05 ± 0.02	0.18 ± 0.03*
Brain stem	0.81 ± 0.49	0.66 ± 0.03	0.34 ± 0.06	0.44 ± 0.15	0.06 ± 0.03	0.22 ± 0.04*
Thalamus	0.84 ± 0.51	0.65 ± 0.04	0.34 ± 0.07	0.44 ± 0.10	0.05 ± 0.03	0.20 ± 0.04*
Hippocampus	1.14 ± 0.74	0.78 ± 0.06	0.47 ± 0.15	0.45 ± 0.18	0.08 ± 0.05	0.24 ± 0.09*
Cortex	0.79 ± 0.50	0.65 ± 0.02	0.32 ± 0.07	0.43 ± 0.10	0.05 ± 0.03	0.19 ± 0.03*
CN	0.87 ± 0.48	0.65 ± 0.04	0.34 ± 0.07	0.45 ± 0.05*	0.06 ± 0.02	0.19 ± 0.03*
Lung	0.65 ± 0.35	1.17 ± 0.15*	0.48 ± 0.03	1.08 ± 0.15*	0.18 ± 0.07	0.52 ± 0.09*
Heart	0.69 ± 0.36	1.38 ± 0.11*	0.45 ± 0.02	1.14 ± 0.16*	0.16 ± 0.08	0.48 ± 0.12*
Liver	11.85 ± 0.33	8.37 ± 0.36	14.43 ± 1.48	7.85 ± 0.41*	3.08 ± 1.44	4.00 ± 0.22
Left kidney	1.37 ± 0.69	2.47 ± 0.30*	1.01 ± 0.16	2.27 ± 0.32*	0.54 ± 0.06	2.18 ± 0.51*
Right kidney	1.34 ± 0.69	2.46 ± 0.18*	0.97 ± 0.12	2.30 ± 0.35*	0.48 ± 0.15	2.30 ± 0.81*
Spleen	0.74 ± 0.27	1.00 ± 0.05	0.55 ± 0.20	1.14 ± 0.50*	0.15 ± 0.07	0.44 ± 0.11*
Pancreas	1.27 ± 0.56	1.83 ± 0.16	0.85 ± 0.23	1.53 ± 0.15*	0.22 ± 0.09	0.57 ± 0.15*
Muscle	0.47 ± 0.23	0.90 ± 0.17*	0.43 ± 0.03	0.72 ± 0.09*	0.11 ± 0.04	0.32 ± 0.06*
Bone	0.30 ± 0.14	0.48 ± 0.02	0.29 ± 0.04	0.50 ± 0.03*	1.08 ± 0.33	1.22 ± 0.31
Urine	0.10 ± 0.10	0.20 ± 0.29	1.78 ± 1.21	0.85 ± 0.79*	20.55 ± 8.76	3.20 ± 2.32
Bladder	0.40 ± 0.12	0.71 ± 0.09*	0.48 ± 0.08	0.84 ± 0.13*	1.58 ± 1.90	0.73 ± 0.54
Blood	0.38 ± 0.18	0.93 ± 0.14*	0.34 ± 0.02	0.79 ± 0.12*	0.15 ± 0.07	0.43 ± 0.07*
Stomach	0.80 ± 0.24	0.86 ± 0.32	0.53 ± 0.09	0.97 ± 0.63	0.68 ± 0.41	1.28 ± 1.08
Skin	0.46 ± 0.17	0.66 ± 0.16	0.52 ± 0.16	0.66 ± 0.10	0.10 ± 0.04	0.71 ± 0.66
Adipose	0.29 ± 0.09	0.33 ± 0.05	0.24 ± 0.07	0.38 ± 0.05*	0.14 ± 0.04	0.25 ± 0.05*
Feces	0.36 ± 0.12	0.79 ± 0.17*	0.51 ± 0.05	1.06 ± 0.21*	2.77 ± 0.79	3.25 ± 0.96
SI	1.94 ± 1.12	3.12 ± 0.27*	3.33 ± 0.49	3.22 ± 0.15*	5.22 ± 0.09	10.72 ± 1.95*
LI	1.09 ± 0.45	1.47 ± 0.17	0.80 ± 0.06	1.48 ± 0.31*	0.57 ± 0.32	1.22 ± 0.63
Cecum	0.54 ± 0.07	0.73 ± 0.03*	0.54 ± 0.03	0.93 ± 0.16*	4.67 ± 4.60	1.79 ± 0.60
Eyes	0.30 ± 0.12	0.35 ± 0.05	0.20 ± 0.03	0.36 ± 0.04*	−	0.29 ± 0.09

*CN* caudate nucleus; *SI* small intestine; *LI* large intestine.

*P < 0.05 [^18^F]**2** vs [^18^F]**2** + 20 µg of **2**.

## Conclusions

Two new isonicotinamide based candidates, [^18^F]**2** and [^18^F]**6,** are successfully synthesized. Both compounds **2** and **6** demonstrated nanomolar affinity towards GSK-3α and GSK-3β and high blood–brain barrier permeability. However, during the serum stability analysis PET probe [^18^F]**6** was found to be unstable and therefore precluded from further in vivo analysis, whereas PET probe [^18^F]**2** was found to be stable in both mouse and human serums, and thus used for in vivo evaluation. PET probe [^18^F]**2** showed a SUV of 0.92 ± 0.28 (*n* = 6) at 5 min post injection in wild-type FVB/NJ mouse brain, which was significantly higher than for previously reported isonicotinamide based PET probes used in GSK-3α/β imaging. The observed binding affinity, blood–brain barrier permeability and stability of PET probe [^18^F]**2** in serum along with its moderate brain uptake in wild-type FVB/NJ mice certainly suggests to further evaluate its brain uptake, selectivity, and retention in an in-vivo animal model of validated GSK-3 expression including AD.

## Methods

### General consideration

All chemicals and solvents were purchased from various commercial manufacturer (Sigma-Aldrich, Fisher Scientific, and Alfa Aesar) and used as received without further purification unless otherwise stated. TLC analysis of reaction mixtures was developed on alumina plates, compounds were visualized under UV light and/or by treatment with a solution of *p*-anisaldehyde followed by heating. The NMR spectra of all compounds including ^1^H NMR, ^13^C NMR and ^19^F NMR were recorded on a Bruker Ultra shield Advance III HD 500 MHz spectrometer. High-resolution mass spectra were recorded on a MICROMASS ESI-TOF MS from the University of Illinois Urbana-Champaign. Oasis^®^ HLB plus short (Part No.186008083), Sep-pak C18 plus short (Part No. WAT020515) and Sep-Pak tC18 plus short cartridges (Part No. WAT023501) were purchased from Waters Corporation. Chromafix 30-PSHCO_3_ cartridges (Lot. No 092.101) were purchased from Synthra GmbH. An aluminum heating block was used as the heating source for all radiochemical reactions. Radio-TLC analysis of reaction mixtures was developed on glass microfiber chromatography paper impregnated with silica gel and analyzed using Eckert & Ziegler AR-2000 radio-TLC imaging scanner. HPLC purifications were performed on a Shimadzu HPLC system. Semipreparative HPLC was performed on a Synergy 4µ Fusion-RP 80 Ao column 20 × 10.0 mm, at a UV detector wavelength of 238 nm using solvent A (H_2_O + 0.1% TFA) and solvent B (CH_3_CN + 0.1% TFA) with a flow rate of 4 mL/min, by applying a gradient method (0–2 min = 2% B, 2–30 min = 2% B to 80% B). Preparative HPLC was performed on a Luna-5 µm C18(2) 100 A^o^ column 250 × 21.2 mm, at a UV detector wavelength of 238 nm using solvent A (H_2_O + 0.1% TFA) and solvent B (CH_3_CN + 0.1% TFA) with a flow rate of 4 mL/min, by applying a gradient method (0–2 min = 2% B, 2–40 min = 2% B to 98% B). Final purity and identity of compounds were determined by analytical HPLC performed on a Phenomenex -Jupiter, 5 μm C18(2) 300 A^o^, LC Column 250 × 4.6 mm, at a UV detector wavelength of 238 nm using solvent A (H_2_O + 0.1% TFA) and solvent B (CH_3_CN + 0.1% TFA) with a flow rate of 1.0 mL/min by applying a gradient method (0–10 min = 16% B to 23% B, 10–30 min = 23% B to 35% B, For [^18^F]**2** 17.3 min retention time and [^18^F]**6**, 28.0 min retention time). All radiolabeling experiments were performed manually.

### Synthesis of compound-1

2-(cyclopropanecarboxamido)*-N*-(4-iodopyridin-3-yl)isonicotinamide (**1)** was synthesized by following previously described protocols^44–47^. The obtained product was characterized by ^1^H and ^13^C NMR spectroscopic data after purification and were consistent with previously reported values.

### Procedure A—General procedure for Suzuki cross coupling

A 20 mL round bottom flask was charged with 2-(cyclopropanecarboxamido)-*N*-(4-iodopyridin-3-yl)isonicotinamide (**1**), (204 mg, 0.5 mmol), corresponding substituted phenylboronic acid pinacol ester or substituted phenylboronic acid (0.6 mmol), K_2_CO_3_ (202.5 mg, 1.5 mmol) and Pd(PPh_3_)_4_ (173.79 mg, 0.15 mmol) under nitrogen environment. Degassed 1,4-dioxane (4.0 mL) and water (1.0 mL) was added to the reaction mixture. Resultant mixture was stirred at 80 °C in an oil bath for 8 h and after completion, reaction mixture was cooled to room temperature. Obtained reaction mixture was diluted with H_2_O (10 mL) and extracted with EtOAc (3 × 10 mL). The combined organic phase was dried over anhydrous Na_2_SO_4_ and concentrated in vacuo. The crude material was purified by silica-gel column chromatography (CHCl_3_:CH_3_OH 1:0 to 0.9:0.1) to afford pure compounds.

### Synthesis of 2-(cyclopropanecarboxamido)-*N*-(4-(4-fluorophenyl)pyridin-3-yl)isonicotinamide (2)

The title compound was synthesized following procedure **A** using 2-(cyclopropanecarboxamido)-*N*-(4-iodopyridin-3-yl)isonicotinamide (**1)** (204 mg, 0.5 mmol) and 4-flouro phenyl boronic acid (84 mg, 0.6 mmol) as coupling partners. The compound **2** was isolated as a white powder. Yield: 65% (300 mg, R*f* = 0.4, 1:9 methanol: chloroform). Mp 162-164 °C. IR (Neat): ν_max_ 3285, 3031, 1691, 1649, 1560, 1533, 1482, 1350, 1416, 1258, 1177, 830 and 698 cm^-1^; ^1^H NMR (DMSO-d_6_, 500 MHz) δ 10.99 (1H, s), 10.40 (1H, s), 8.64 (1H, s), 8.57 (1H, d, *J* = 5.0 Hz), 8.46–8.44 (2H, m), 7.55 (2H, t, *J* = 7.5 Hz), 7.48 (1H, d, *J* = 4.5 Hz), 7.39 (1H, d, *J* = 3.5 Hz), 7.29 (2H, t, *J* = 8.5 Hz), 2.03 (1H, pent, *J* = 6.5 Hz), 0.84 (4H, d, *J* = 7.0 Hz); ^13^C NMR (125 MHz, DMSO-d_6_) δ 173.3 (C), 165.4 (C), 162.6 (C, d, *J* = 237.5 Hz), 153.2 (C), 149.8 (CH), 149.0 (CH), 148.5 (CH), 145.0 (C), 143.7 (C), 133.3 (C, d,* J* = 3.75 Hz), 131.3 (C), 130.8 (2 × CH, d,* J* = 8.75 Hz), 124.9 (CH), 117.1 (CH), 116.1 (2 × CH, d,* J* = 21.25 Hz), 112.0 (CH), 14.7 (CH), 8.3 (2 × CH_2_); ^19^F NMR (470 MHz, DMSO-d^6^) δ ppm: -113.47 (CF). HRMS (ESI-TOF) m/z: [M + H] + calcd for C_21_H_18_FN_4_O_2_, 377.1414; found, 377.1417.

### Synthesis of 2-(cyclopropanecarboxamido)-*N*-(4-(4-hydroxyphenyl)pyridin-3-yl)isonicotinamide (3)

The tittle compound was synthesized following the general procedure **A** using 2-(cyclopropanecarboxamido)-*N*-(4-iodopyridin-3-yl)isonicotinamide (**1)**, (204 mg, 0.5 mmol) and 4-hydroxy phenylboronic acid pinacol ester (132.06 mg, 0.6 mmol) as a coupling partners. The compound **3** was isolated as a light-yellow powder; Yield: 50% (94 mg, R_*f*_ = 0.3, 1:9 methanol: chloroform). Mp 150- 152 °C. IR (Neat): ν_max_ 3170, 1684, 1611,1558, 1516, 1481, 1435, 1405, 1319, 1276, 1170, 831, and 722 cm^-1^ ; ^1^H NMR (DMSO-d_6_, 500 MHz) δ 10.98 (1H, s), 10.35 (1H, s), 9.71 (1H, s), 8.59–8.52 (2H, m), 8.48 (2H, s), 7.43 (2H, d, *J* = 16.0 Hz), 7.37 (2H, d, *J* = 6.5 Hz), 6.82 (2H, d, *J* = 7.0 Hz), 2.03 (1H, s), 0.85 (4H, s); ^13^C NMR (125 MHz, DMSO-d_6_) δ 173.3 (C), 165.4 (C), 158.4 (C), 153.3 (C), 149.6 (CH), 149.0 (CH), 148.0 (CH), 146.3 (C), 143.8 (C), 131.3 (C), 130.0 (2 × CH), 127.2 (C), 124.8 (CH), 117.1 (CH), 116.0 (2 × CH), 112.0 (CH), 14.7 (CH), 8.3 (2 × CH_2_); HRMS (ESI-TOF) m/z: [M + H] + calcd for C_21_H_19_N_4_O_3_, 375.1457; found, 375.1446.

### Synthesis of 2-(cyclopropanecarboxamido)-*N*-(4-(4-(4,4,5,5-tetramethyl-1,3,2-dioxaborolan-2-yl)phenyl)pyridin-3-yl)isonicotinamide (4)

A 20 mL round bottom flask was charged with 2-(cyclopropanecarboxamido)-*N*-(4-iodopyridin-3-yl)isonicotinamide (**1)**, (204 mg, 0.5 mmol) 1,4-phenylelediboronicacid pinacol ester (495 mg, 1.5 mmol), K_2_CO_3_ (202.5 mg, 1.5 mmol)) and Pd(PPh_3_)_4_ (57.9 mg, 0.05 mmol) under nitrogen environment. Degassed 1,4-dioxane (4.0 mL), water (1.0 mL) and ethanol (1.0 mL) were added to the reaction mixture. The mixture was stirred at 80 °C in an oil bath for 8 h and after completion reaction mixture was cooled to room temperature. The obtained reaction mixture was diluted with H_2_O (10 mL) and extracted with EtOAc (3 × 10 mL). The combined organic phase was dried over anhydrous Na_2_SO_4_ and concentrated in vacuo. The crude material was purified by silica-gel column chromatography (CHCl_3_:CH_3_OH 1:0 to 0.9:0.1) to afford pure compound. The compound **4** was isolated as a white powder. Yield: 10% (63 mg, R*f* 0.6, 1:9 methanol: chloroform). Mp 260-262 °C; IR (Neat): ν_max_ 3004, 1742, 1563, 1359, 1210, 1144, 1090, 1021, 746 and 657 cm^–1^; ^1^H NMR (DMSO-d_6_, 500 MHz) δ 10.98 (1H, s), 10.42 (1H, s), 8.66 (1H, s), 8.58 (1H, d, *J* = 5.0 Hz), 8.45 (2H, d,* J* = 5.0 Hz ), 7.74 (2H, d,* J* = 8.0 Hz), 7.54 (2H, d, *J* = 7.5 Hz), 7.47 (1H, d, *J* = 5.0 Hz), 7.40 (1H, d, *J* = 4.0 Hz), 2.03–1.99 (1H, m), 1.29 (12H, s), 0.87–0.83 (4H, m); ^13^C NMR (125 MHz, DMSO-d_6_) δ 173.3 (C), 165.4 (C), 153.2 (C), 149.8 (CH), 149.0 (CH), 148.5 (CH), 145.4 (C), 143.8 (C), 140.0 (C), 135.1 (2 × CH), 131.3 (C), A signal for the quaternary carbon directly attached to the boron atom was not observed due to ^10^B and ^11^B quadrupolar coupling. 128.1 (2 × CH), 124.8 (CH), 117.1 (CH), 111.9 (CH), 84.2 (2 × C), 25.1 (4 × CH_3_), 14.7 (CH), 8.3 (2 × CH_2_); HRMS (ESI-TOF) m/z: [M + H] + calcd for C_27_H_30_BN_4_O_4_, 485.2360; found, 485.2363.

### Synthesis of 2-(4-(3-(2-(cyclopropanecarboxamido)isonicotinamido)pyridin-4-yl)phenoxy)-1,3-bis(2,6-diisopropylphenyl)-1*H*-imidazol-3-ium chloride (5)

A 10 mL vial was charged with compound **3** (187.06 mg, 0.5 mmol), 2-chloro-1,3-bis(2,6-diisopropylphenyl)imidazolium chloride (229.74 mg, 0.5 mmol) and silver carbonate (68.93 mg, 0.25 mmol). Anhydrous chloroform (5.0 mL) was added to the reaction mixture. The mixture was stirred at 60 °C in an oil bath for 4.5 h and cooled to room temperature. The precipitate was removed by filtration and the filtrate was concentrated in vacuo. The crude material was recrystallized in CHCl_3_ to afford pure compound. The compound **5** was isolated as a white powder. Yield: 60% (501 mg, R_*f*_ = 0.2, 1:9 methanol: chloroform). Mp 210-212 °C. IR (Neat): ν_max_ 2971, 1748, 1730, 1682, 1646, 1506, 1452, 1261, 1147 and 749 cm^–1^; ^1^H NMR (DMSO-d_6_, 500 MHz) δ 11.03 (1H, s), 10.49 (1H, s), 8.55–8.54 (2H, m), 8.48 (2H, s), 8.45 (1H, d, *J* = 4.5 Hz), 8.40 (1H, s), 7.53 (2H, t, *J* = 8.0 Hz), 7.44 (3H, d, *J* = 7.0 Hz), 7.38 (4H, d, *J* = 7.5 Hz), 7.27 (1H, t, *J* = 5.0 Hz), 6.82 (2H, d, *J* = 8.0 Hz), 2.53–2.51 (4H, m), 2.08 (1H, s), 1.21 (12H, d, *J* = 6.5 Hz), 1.12 (12H, d, *J* = 6.5 Hz), 0.83 (4H, d, *J* = 10.5 Hz); ^13^C NMR (125 MHz, DMSO-d_6_) δ 173.3 (C), 165.0 (C), 153.5 (C), 153.2 (C), 150.2 (CH), 148.9 (CH), 148.7 (CH), 145.5 (4 × C), 144.3 (C), 144.0 (C), 143.2 (C), 135.6 (C), 132.7 (2 × CH), 131.2 (C), 131.1 (2 × CH), 127.5 (2 × C), 125.5 (4 × CH), 124.6 (CH), 123.2 (2 × CH), 117.2 (CH), 117.1 (2 × CH), 112.1 (CH), 29.3 (4 × CH), 25.4 (4 × CH_3_), 23.0 (4 × CH_3_), 14.7 (CH), 8.2 (2 × CH_2_); HRMS (ESI-TOF) m/z: [M–Cl] + calcd for C_48_H_53_N_6_O_3_, 764.4174; found, 764.4174.

### Synthesis of 4-(3-(2-(cyclopropanecarboxamido)isonicotinamido)pyridin-4-yl)phenyl sulfurofluoridate (6)

A 10 mL round bottom flask was charged with compound **3** (187.06 mg, 0.5 mmol), 1-(Fluorosulfuryl)-2,3-dimethyl-1*H*-imidazol-3-ium trifluoromethanesulfonate (197 mg, 0.6 mmol) and triethyl amine (104.3 μL, 0.75 mmol). Anhydrous acetonitrile (3.0 mL) was added to the reaction mixture. The mixture was stirred for 1 h at room temperature. After completion reaction mixture was concentrated in vacuo. The obtained crude material was purified by silica-gel column chromatography (CHCl_3_:CH_3_OH 1:0 to 0.9:0.1) to afford pure compound. The compound **6** was isolated as a pale-yellow powder. Yield: 50% (305 mg, R_*f*_ = 0.4, 1:9 methanol: chloroform). Mp 216- 218 °C; IR (Neat): ν_max_ 3255, 1739, 1682, 1649, 1500., 1560, 1231, 1410, 917, 1144, 821, 746 and 716 cm^–1^; ^1^H NMR (DMSO-d_6_, 500 MHz) δ 10.97 (1H, s), 10.47 (1H, s), 8.68 (1H, s), 8.60 (1H, d, *J* = 5.0 Hz), 8.44 (1H, d, *J* = 5.0 Hz), 8.40 (1H, s), 7.69 (4H, t, *J* = 10.5 Hz), 7.53 (1H, d, *J* = 5.0 Hz), 7.35 (1H, d, *J* = 4.5 Hz), 2.04–2.02 (1H, m), 0.84 (4H, d, *J* = 7.5 Hz); ^13^C NMR (125 MHz, DMSO-d_6_) δ 173.3 (C), 165.5 (C), 153.2 (C), 150.0 (C), 149.8 (CH), 149.0 (CH), 148.6 (CH), 144.1 (C), 143.7 (C), 138.0 (C), 131.4 (C), 131.1 (2 × CH), 124.9 (CH), 121.9 (2 × CH), 117.1 (CH), 111.9 (CH), 14.6 (CH), 8.2 (2 × CH_2_); ^19^F NMR (23 °C, 470 MHz, DMSO-d_6_) δ ppm: 39.0 (CF). HRMS (ESI-TOF) m/z: [M + H] + calcd for C_21_H_17_FN_4_O_5_S, 457.0982; found, 457.0978.

### Radiolabeling

#### Synthesis of [^18^F]2 from compound 5 via ^18^F-deoxyfluorination methodology

Cyclotron generated [^18^F]fluoride solution (1.0 mL, or 1.85 GBq) was trapped on an anion exchange cartridge (Chromafix 30-PSHCO_3_) with > 99% efficiency. [^18^F]fluoride trapped cartridge was washed with 1.0 mL mixture of 2-butanone and ethanol in 10:1 (v:v) ratio to maximize the elution efficiency. The [^18^F]fluoride eluent was prepared by dissolving the compound **5** (8.0 mg) in a mixture of 2-butanone: EtOH: NBu_3_ in the v:v ratio of 10: 1: 0.1 (1.0 mL). The trapped [^18^F]fluoride was transferred to a 5.0 mL reaction vial sealed with a teflon cap with eluent using a 3.0 mL syringe (1.1 GBq, with 60% efficiency) and stirred at 130 °C for 20 min. After completion, the reaction vial was cooled to room temperature for 1 min. [^18^F]Fluoride labeling (radio fluorination) was checked using silica r-TLC with 5% MeOH-95% CHCl_3_ as a mobile phase. The reaction mixture was transferred into a falcon tube (50 mL) containing 30 mL of H_2_O. The contents were loaded to an Oasis HLB plus short cartridge for purification, followed by passing air (36 mL) through the cartridge. The trapped [^18^F]**2** was washed with water (5.0 mL), dried with air (36 mL) and subsequently eluted from the cartridge into a 5.0 mL vial with ethanol (1.0 mL) and was measured to contain 0.014 GBq (n = 2, 0.75 ± 0.07% of the initial trapped activity). This method gave 0.99 ± 0.099% (*n* = 2) decay-corrected radiolabeling yield. Total synthesis time was 45 min (*n* = 2) from the time fluoride-18 was trapped on the anion exchange cartridge. The identity of the synthesized [^18^F]**2** PET probe was confirmed by matching the retention time with the reference standard **2** on an analytical HPLC. HPLC was performed on a Phenomenex-Jupiter, 5 μm C18(2) 300 A^o^, LC Column 250 × 4.6 mm at a UV detector wavelength of 238 nm, using solvent A (H_2_O + 0.1% TFA) and solvent B (CH_3_CN + 0.1% TFA) with a flow rate of 1.0 mL/min, by applying a gradient method (0–10 min = 16% B to 23% B, 10–30 min = 23% B to 35% B) showing a 16.9 min retention time. Radiolabeling was performed manually.

#### Synthesis of [^18^F]2 from compound 4 via copper mediated oxidative ^18^F-flourination methodology

To increase the radiosynthesis yield of [^18^F]**2**, copper mediated oxidative ^18^F-flourination was attempted as described below. Cyclotron generated fluoride-18 solution (3.0 mL, 5.1 ± 0.94 GBq, n = 13) was trapped on an anion exchange cartridge (Chromafix 30-PSHCO_3_) with > 99% efficiency. Fluoride-18 ion eluent was prepared by dissolving K 2.2.2 (8.0 mg) and K_2_CO_3_ (4.0 mg) in CH_3_CN: H_2_O in v:v ratio of 8:2 (1.0 mL). The trapped [^18^F]fluoride was eluted with eluent and transferred to a teflon capped 5.0 mL reaction vial using a 3.0 mL syringe with > 97% elution efficiency. The resultant reaction vial was heated at 110 ^o^C under N_2_ flow to remove water-acetonitrile azeotrope. Overall, three cycles of CH_3_CN (1.0 mL × 3) addition and evaporation were performed to completely dry the [^18^F]fluorideK^+^-K 2.2.2 reagent. Finally, 8.2 mg of compound **4** with 27 mg of Cu(OTf)_2_(Py)_4_ dissolved in DMI (0.4 mL) was added to the reaction vial, and the reaction mixture was stirred at 120 °C for 20 min. After completion, the reaction vial was cooled to room temperature for 1 min and diluted with 0.5 mL EtOAc. Fluoride-18 labeling (radio fluorination) was checked using silica *r*-TLC with 5% MeOH-95% CHCl_3_ as a mobile phase. The reaction contents were transferred into a falcon tube (50 mL) containing 30 mL of H_2_O. The contents were loaded to an Oasis HLB plus short cartridge for purification, followed by pushing air (36 mL) through the cartridge. The trapped [^18^F]**2** was washed with water (5.0 mL), dried with air (36 mL) and subsequently eluted from the cartridge into a 5.0 mL vial with ethanol (1.0 mL). Next, [^18^F]**2** in 1.0 ml of ethanol was purified by semipreparative HPLC Synergy 4µ Fusion -RP 80 A^o^ column 20 × 10.0 mm, at a UV detector wavelength of 238 nm using solvent A (H_2_O + 0.1% TFA) and solvent B (CH_3_CN + 0.1% TFA) with a flow rate of 4 mL/min, by applying a gradient method (0–2 min = 2% B, 2–30 min = 2% B to 80% B) showing retention time ~ 17.5–18.5 min or preparative HPLC using a Luna- 5 µm C18(2) 100 A^o^ column 250 × 21.2 mm, at a UV detector wavelength of 238 nm using solvent A (H_2_O + 0.1% TFA) and solvent B (CH_3_CN + 0.1% TFA) with a flow rate of 4 mL/min, by applying a gradient method (0–2 min = 2% B, 2–40 min = 2% B to 98% B), showing ~ 31.5–32.5 min retention time. The final product was concentrated using a standard C-18 Sep-Pak based trap and release method. The product was eluated with ethanol. Finally, [^18^F]**2** was formulated in 0.9% isotonic NaCl solution containing 10% ethanol (9 mL (*n* = 9) and 10 mL (*n* = 4)) and filtered through a 0.22 μm filter into a 50 mL sterilized vial. The obtained [^18^F]**2** measured to contain 0.33 ± 0.08 GBq (n = 13, 6.61 ± 1.33% (n = 13) of the initial trapped activity). This method gave 12.08 ± 2.45% (n = 13) decay-corrected radiochemical yield and a molar activity (A_m_) of 87.27 ± 77.26 (*n* = 13) GBq/µmole of [^18^F]**2** with > 99% radiochemical purity at the end of the formulation. Total synthesis, purification and formulation time was 96.69 ± 8.05 min (n = 13) from the time fluoride-18 was trapped on the anion exchange cartridge. The identity of the synthesized [^18^F]**2** PET probe was confirmed by co-injecting with its reference standard **2** and matching the retention time of 16.9 min within 5% deviation range on an analytical HPLC. Analytical HPLC was performed on a Phenomenex -Jupiter, 5 μm C18(2) 300 A^o^, LC Column 250 × 4.6 mm at a UV detector wavelength of 238 nm using solvent A (H_2_O + 0.1% TFA) and solvent B (CH_3_CN + 0.1% TFA) with a flow rate of 1.0 mL/min, by applying a gradient method (0–10 min = 16% B to 23% B, 10–30 min = 23% B to 35% B). After product conformation moved to all in vitro and in vivo studies. Radiosynthesis, purification and formulation were performed manually. The high variation in molar activity of [^18^F]**2** is mainly due to the manual radiosynthesis, and long-term instability of compound **4** (precursor) used for F-18 labeling. Freshly prepared precursor compound **4** gave better radiochemical yield than when radiosynthesis was performed with a 1–2-week-old precursor.

#### Synthesis of [^18^F]6 from compound 6 via ^18^F-SuFEx click chemistry

Cyclotron generated fluoride-18 solution (3.0 mL, 2.63 ± 1.08 GBq, n = 6) was trapped on an anion exchange cartridge (Chromafix 30-PSHCO_3_) with > 99% efficiency. Fluoride-18 eluent was prepared by dissolving K 2.2.2 (8.0 mg) and K_2_CO_3_ (4.0 mg) in CH_3_CN: H_2_O in v: v ratio of 8:2 (1.0 mL). The trapped [^18^F]fluoride was transferred in to a 5.0 mL reaction vial sealed with teflon cap with eluent using a 3.0 mL syringe with > 97% efficiency. The reaction vial was heated at 110 ^o^C under N_2_ flow to remove water-acetonitrile azeotrope. Overall, three cycles of CH_3_CN (1.0 mL × 3) addition and evaporation were performed to completely dry the [^18^F]fluoride ion^-^K^+^-K 2.2.2 reagent. Finally, 3.0 mg of compound **6** dissolved in CH_3_CN (1.0 mL) was added to the reaction vial having [^18^F]fluoride ion^-^K^+^-K 2.2.2 reagent as descried previously, and the reaction mixture was stirred at room temperature for 5 min. Fluoride-18 labeling was checked using silica r-TLC with 5% MeOH-95% CHCl_3_ as a mobile phase. The reaction contents were transferred into a centrifuge tube (50 mL) containing 30 mL of H_2_O. The contents were subjected to a C-18 cartridge for purification, followed by pushing air (36 mL) through the cartridge. The trapped [^18^F]**6** was washed with water (5 mL), dried with air (36 mL), subsequently eluted from the cartridge into a 5.0 mL vial with ethanol (1.0 mL). Final [^18^F]**6** in 1.0 ml of ethanol was purified by a semipreparative HPLC using a Synergy 4µ Fusion -RP 80 A^o^ column 20 × 10.0 mm at a UV detector wavelength of 238 nm using solvent A (H_2_O + 0.1% TFA) and solvent B (CH_3_CN + 0.1% TFA) with a flow rate of 4 mL/min, by applying a gradient method (0–2 min = 2% B, 2–30 min = 2% B to 80% B), showing ~ 21.5–22.5 min retention time. The final product was concentrated using a standard C-18 Sep-Pak based trap and release method. The product was eluated with 1.0 mL of ethanol, formulated in 9 mL of 0.9% isotonic NaCl solution and filtered through a 0.22 μm filter into a 50 mL sterilized vial. Total final volume is 10 mL. the obtained [^18^F]**6** measured to contain 0.55 ± 0.49 GBq (n = 6, 19.60 ± 10.30% of the initial trapped activity). This method gave 26.03 ± 13.69% (n = 6) decay-corrected radiochemical yield (RCY) and molar activity (A_m_) of 0.37 ± 0.14 (*n* = 6) GBq/µmole of [^18^F]**6** > 99% radiochemical purity (RCP) at the end of the formulation. Total synthesis, purification and formulation time was 45 min (*n* = 6) from the time fluoride-18 was trapped on an anion exchange cartridge. The identity of the synthesized [^18^F]**6** PET probe was confirmed by co-injecting with their reference standard **6** and matching retention time of 27.6 min within 5% deviation range on an analytical HPLC. Analytical HPLC was performed on a Phenomenex-Jupiter, 5 μm C18(2) 300 A^o^, LC Column 250 × 4.6 mm at a UV detector wavelength of 238 nm using solvent A (H_2_O + 0.1% TFA) and solvent B (CH_3_CN + 0.1% TFA) with a flow rate of 1.0 mL/min, by applying a gradient method (0–10 min = 16% B to 23% B, 10–30 min = 23% B to 35% B). After product conformation moved to all in vitro studies. Radiosynthesis, purification and formulation were performed manually.

### In vitro assays

Blood–brain barrier permeability assessment, inhibition assays and serum radiochemical stability assays were performed by following our previously reported protocol as briefly mentioned below^46^.

### Blood–brain barrier permeability assessment

In vitro parallel artificial membrane permeability assay (PAMPA) was performed by Pion Inc., East Sussex, UK for assessing blood–brain permeability of nonradioactive version of PET probes [^18^F]2, [^18^F]6, propranolol HCl and atenolol. The blood–brain permeability was expressed through the permeability coefficient, P (10^–6^ cm/s) and -logP_e_.

### Determination of IC_50_

The phosphorylation of glycogen synthase kinase substrate by the human GSK-3α or GSK-3β was performed in the presence of 0–400 nM of compound **2** or **6** as per manufacturer’s instructions using human GSK-3α/β kinase enzyme assay system (Promega Corporation, Madison, WI). Phosphorylation of glycogen synthase kinase substrate was quantified using a luminescent ADP-Glo™ Kinase assay system (Promega Corporation, Madison, WI). The IC_50_ of compounds **2** and **6** were determined from the resulting dose–response curve and IC_50_ curve fitting tool of the GraphPad Prism 10 (GraphPad Software, San Diego, CA).

### Serum stability analysis

Stability of [^18^F]**2** and [^18^F]**6** was assessed in isotonic NaCl solution and human and mouse serums. To perform this stability assay, 100 µL of [^18^F]**2** (~ 37.0 MBq) or 100 µL of [^18^F]**6** (~ 37.0 MBq) as in final formulation was added to 100 µL of human serum or mouse serum in a microcentrifuge tube. The resultant mixture was incubated at 37 °C for 120 min. To assess the radiochemical stability of the probes, a small aliquot (~ 0.5 µL) was taken out from the mixture in triplicate at 0 min, 30 min, 60 min and 120 min during incubation, and analyzed on a rad-TLC using 0.5:9.5 methanol: chloroform as a mobile phase using *i*-TLC to estimate the stability of [^18^F]**2** or [^18^F]**6** at different time points post-incubation. After Stability preparative processes initial *i*-TLC development was considered as 0 min.

### In vivo PET imaging and ex-vivo biodistribution

Uptake of PET probe [^18^F]**2** (1.40 ± 0.61 MBq) was assessed with and without co-administration of **2** in 10–13-week-old wild-type FVB/NJ mice (The Jackson Laboratory, Bar Harbor, ME) after tail vein injection followed by PET imaging at 5 min, 10 min, 15 min, 20 min, 25 min, 30 min, 40 min, 50 min, 60 min and 120 min post injection. Additionally, ex vivo biodistribution was performed at 5 min, 10 min and 120 min post tail vein injection. To prepare [^18^F]**2** formulation for injection, 0.76 ± 0.48 µg (2.01 ± 1.27 nmol) of [^18^F]**2** was formulated in 10% ethanol/ 90% isotonic saline. For the blocking experiment, 20.21 ± 1.63 µg (53.75 ± 4.33 nmol) of **2** in DMSO was added to [^18^F]**2** (A_m_ of 87.27 ± 77.26 (*n* = 13) GBq/µmole, having 0.46 ± 0.31 µg or 1.23 ± 0.83 nmol of nonradioactive **2** in it) in the final formulation volume of ~ 100 µL. This study had 12 wild type FVB/NJ male mice with body weight 31.8 ± 2.7 g and 10 wild type FVB/NJ female mice with body weight 27.0 ± 3.6 g at the time of imaging. All the animals were anesthetized for tail vein injection and PET imaging using an induction dose of 3% isoflurane and a maintenance dose of 2–3% isoflurane, which was administered via isoflurane vaporizer. All anesthetized animals were kept warm during imaging using a heat supported imaging chamber. For ex vivo biodistribution studies, animals were sacrificed at 5 min, 10 min and 120 min post-injection by cervical dislocation and tissues were harvested for gamma counting. For PET imaging studies, each animal underwent a 60 min dynamic PET scan (Framing Sequence: 1 × 10 s, 4 × 15 s, 8 × 30 s, 5 × 60 s, 4 × 300 s, 3 × 600 s) immediately post injection followed by 10 min static PET scan at 120 min post-injection on small animal micro-PET/X-ray system, Genesys 4 (Sofie BioSystems, Dulles, VA, USA). The PET images were reconstructed using Genesys 4 imaging software installed on the imaging system. The obtained PET images were visualized and analyzed using image analysis software, “Amide's a medical image data examiner-AMIDE”^53^. PET images were normalized to the standardized uptake value (SUV) where;

$$SUV = \frac{Concentration \,\,of \,\,dose\,\, in\,\, tissue\,\, \left(\mu Ci/g\right) \times Weight \,\,of \,\,the\,\, animal\,\, (g)}{Injected \,\,dose\,\, (\mu Ci) }.$$

The SUV data was presented as coronal, transverse, and sagittal sectional maximum intensity projection image generated using MIM 6 software (MIM Software Inc., Cleveland, OH).

### Statistics

The data generated were compared using unpaired Student’s *t*-test analyses. Differences were considered statistically significant only when *P* value was < 0.05.

### Ethical standards

All the animal studies were performed following institutional guidelines and regulations for animal research and after approval from the Institutional Animal Care and Use Committee (IACUC) of the Mayo Clinic. All methods in relation to animal studies were in accordance with ARRIVE guidelines (https://arriveguidelines.org) which are equivalent to IACUC guidelines and regulations. Anesthetized animals were sacrificed for ex vivo biodistribution at 5 min, 10 min, and 120 min post-injection as per IACUC approved euthanasia method of cervical dislocation.

### Supplementary Information

Supplementary Information.

## Data Availability

All data generated and/or analyzed during this study are included in the main article and as associated supplementary information files.
