# Supplementary material for: Synthesis and preliminary evaluation of novel PET probes for GSK-3 imaging
Source: Sci Rep. 2024 Jul 10;14:15960. doi: 10.1038/s41598-024-65943-z (PMC11237012; doi:10.1038/s41598-024-65943-z)
Supplement: Supplementary file 1 — Supplementary Information. [file 41598_2024_65943_MOESM1_ESM.pdf]

Supplementary Information

Synthesis and preliminary evaluation of novel PET probes for GSK-3 imaging

Surendra Reddy Gundam, [1] Aditya Bansal, [1] Manasa Kethamreddy, [1] Sujala Ghatamaneni, [1] Val J. Lowe, [1] Melissa E. Murray [2] & Mukesh K. Pandey [1]*

[1] Division of Nuclear Medicine, Department of Radiology, Mayo Clinic, Rochester, MN 55905, USA

[2] Department of Neuroscience, Mayo Clinic, Jacksonville, FL 32224, USA.

CONTENTS Page

1. NMR Spectra

1.1 ^{1}H NMR Spectra of compound **2** S-6

1.2 ^{13}C NMR Spectra of compound **2** S-7

1.3 ^{19}F NMR Spectra of compound **2** S-8

1.4 ^{1}H NMR Spectra of compound **3** S-9

1.5 ^{13}C NMR Spectra of compound **3** S-10

1.6 ^{1}H NMR Spectra of compound **4** S-11

1.7 ^{13}C NMR Spectra of compound **4** S-12

1.8 ^{1}H NMR Spectra of compound **5** S-13

1.9 ^{13}C NMR Spectra of compound **5** S-14

1.10 ^{1}H NMR Spectra of compound **6** S-15

1.11 ^{13}C NMR Spectra of compound **6** S-16

1.12 ^{19}F NMR Spectra of compound **6** S-17

2. HPLC Profiles

2.1 **Supplementary Figure S1**: Representative analytical HPLC UV trace of compound **2** S-18

2.2 **Supplementary Figure S2**: Representative analytical HPLC trace of post semipreparative column purified [^{18}F]**2** S-19

2.3 **Supplementary Figure S3**: Representative analytical HPLC trace of post semipreparative column purified [^{18}F]**2** with co-injection of compound **2** S-20

2.4 **Supplementary Figure S4**: Representative analytical HPLC UV trace of compound **6** S-21

2.5 **Supplementary Figure S5**: Representative analytical HPLC trace of post semipreparative column purified [^{18}F]**6** S-22

2.6 **Supplementary Figure S6**: Representative analytical HPLC trace of post semipreparative column purified [^{18}F]**2** with co-injection of compound **6** S-23

3. Calibration Curve

3.1 **Supplementary Figure S7**: Calibration curve of compound **2** used for molar activity calculation**.** S-24

3.2 **Supplementary Figure S8**: Calibration curve of compound **6** used for molar activity calculation. S-25

4. Radio-TLC Profile

4.1 **Supplementary Figure S9**: Radio-TLC of [^{18}F]**2** and [^{18}F]**6** S-26

5. HRMS Spectrum

5.1 HRMS Spectrum and Elemental Composition Report of compound **2** S-27, S-28

5.2 HRMS Spectrum of compound **3** S-29, S-30

5.3 HRMS Spectrum and Elemental Composition Report of compound **4** S-31, S-32

5.4 HRMS Spectrum and Elemental Composition Report of compound **5** S-33, S-34

5.5 HRMS Spectrum and Elemental Composition Report of compound **6** S-35, S-36

6. In vitro Studies

6.1 **Supplementary Table S1**: Stability of [^{18}F]**2** and [^{18}F]**6** in isotonic NaCl solution, mouse, and human serums over time S-37

6.2 **Supplementary Figure S10**: Representative radio Radio-TLCs of [^{18}F]**6** Stability in 0.9% Saline solution over time. S-38

6.3 **Supplementary Figure S11**: Representative radio -TLCs of [^{18}F]**6** Stability in mice serum over time. S-39

6.4 **Supplementary Figure S12**: Representative radio -TLCs of [^{18}F]**6** Stability in human serum over time. S-40

6.5 **Supplementary Figure S13**: Representative radio -TLCs of [^{18}F]**2** Stability in 0.9% Saline solution over time. S-41

6.6 **Supplementary Figure S14**: Representative radio -TLCs of [^{18}F]**2** Stability in mice serum over time. S-42

6.7 **Supplementary Figure S15**: Representative radio -TLCs of [^{18}F]**2** Stability in human serum over time. S-43

6.8 **Supplementary Figure S16**: Non-linear regression fit curves of (A) glycogen synthase kinase 3 alpha (GSK-3α) vs concentration of **2** (nM), (B) glycogen synthase kinase 3 beta (GSK-3β) vs concentration of **2** (nM), (C) glycogen synthase kinase 3 alpha (GSK-3α) vs concentration of **6** (nM) and (D) glycogen synthase kinase 3 beta (GSK-3β) *vs* concentration of **6** (nM), S-44

7. In vivo Studies

7.1 **Supplementary Figure S17:** Representative PET images (coronal and sagittal section) showing uptake of [^{18}F]**2** and [^{18}F]**2** with 20µg of **2** in liver of FVB/NJ mice at different time points post-injection. S-45

7.2 **Supplementary Figure S18:** Representative PET images showing uptake of [^{18}F]**2** and [^{18}F]**2** with 20µg of **2** in whole body of FVB/NJ mice with major uptake in liver at different time points post-injection. S-46

7.3 **Supplementary Table S2.** Uptake of [^{18}F]**2** and [^{18}F]**2** with **2** in brain and liver of FVB/NJ mice at different time points

post-injection assessed by image analysis. Data expressed as standardized uptake value (SUV). The SUVs were calculated by image analysis and each data point is average ± standard deviation. *P < 0.05 [^{18}F]**2** vs [^{18}F]**2** + 20µg of **2** S-47

7.4 **Supplementary Figure S19.** Uptake of [^{18}F]**2** (n=4) and [^{18}F]**2** with 20µg of **2** (n=3) in (A) brain and (B and C) other major organs in FVB/NJ mice at 5 min post intravenous (i.v.) administration. *P < 0.05 [^{18}F]**2** vs [^{18}F]**2** + 20µg of **2**. S-48

7.5 **Supplementary Figure 20.** Uptake of [^{18}F]**2** (n=4) and [^{18}F]**2** with 20µg of **7** (n=3) in (A) brain and (B and C) other major organs in FVB/NJ mice at 10 min post intravenous (i.v.) administration. *P < 0.05 [^{18}F]**2** vs [^{18}F]**2** + 20µg of **2**. S-49

7.6 **Supplementary Figure 21.** Uptake of [^{18}F]**2** (n=3) and [^{18}F]**2** with 20µg of **2** (n=3) in (A) brain and (B and C) other major organs in FVB/NJ mice at 120 min post intravenous (i.v.) administration. *P < 0.05 [^{18}F]**2** vs [^{18}F]**2** + 20µg of **2**. S-50

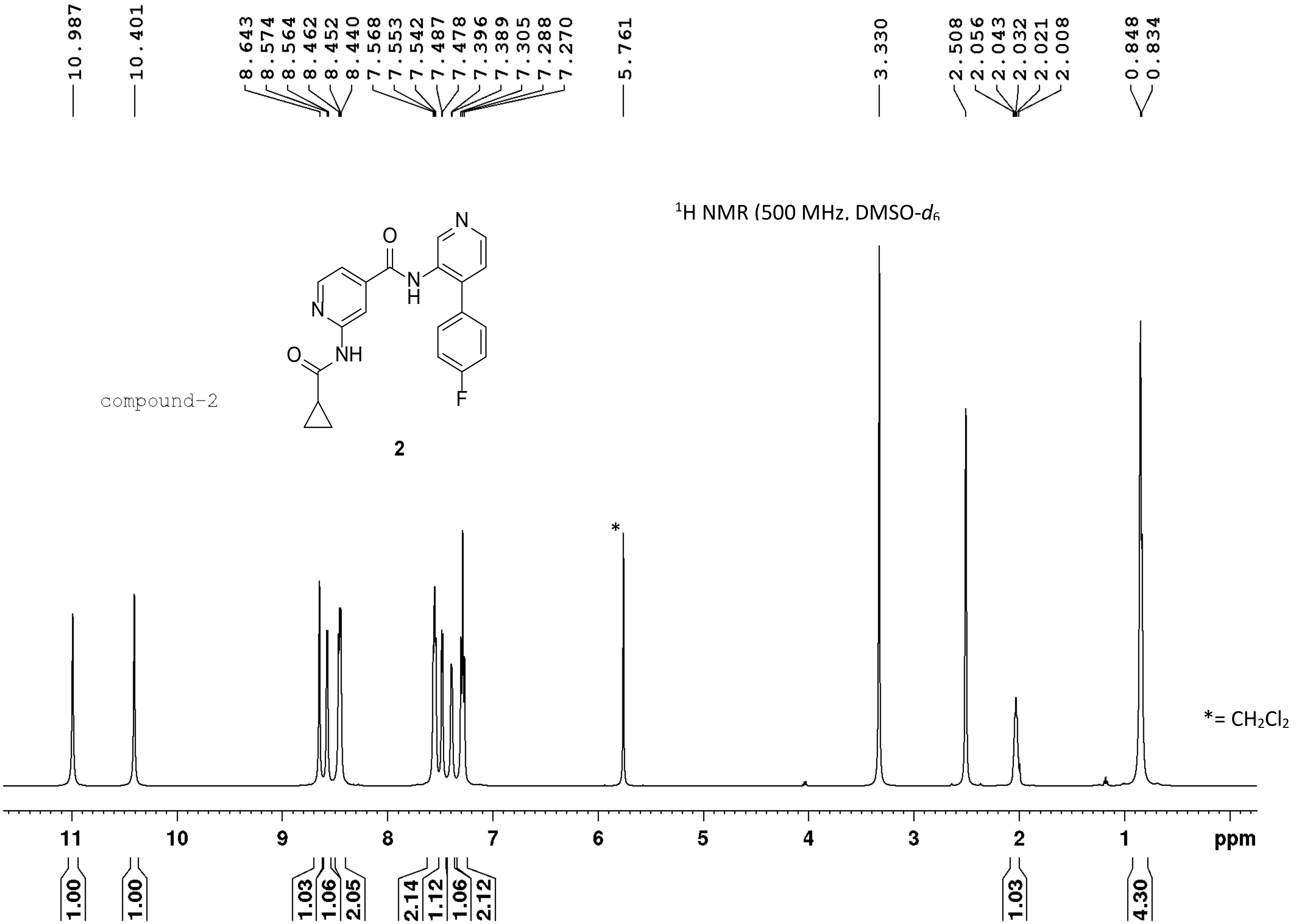

10.987
10.401
8.643 8.574 8.564 8.462 8.452 8.440 7.568 7.553 7.542 7.487 7.478 7.396 7.389 7.305 7.288 7.270
5.761
3.330
2.508 2.056 2.043 2.032 2.021 2.008
0.848 0.834
1H NMR (500 MHz, DMSO-d6
compound-2
2
*
*= CH2Cl2
11 10 9 8 7 6 5 4 3 2 1 ppm
1.00 1.00 1.03 1.06 2.05 2.14 1.12 1.06 2.12 1.03 4.30

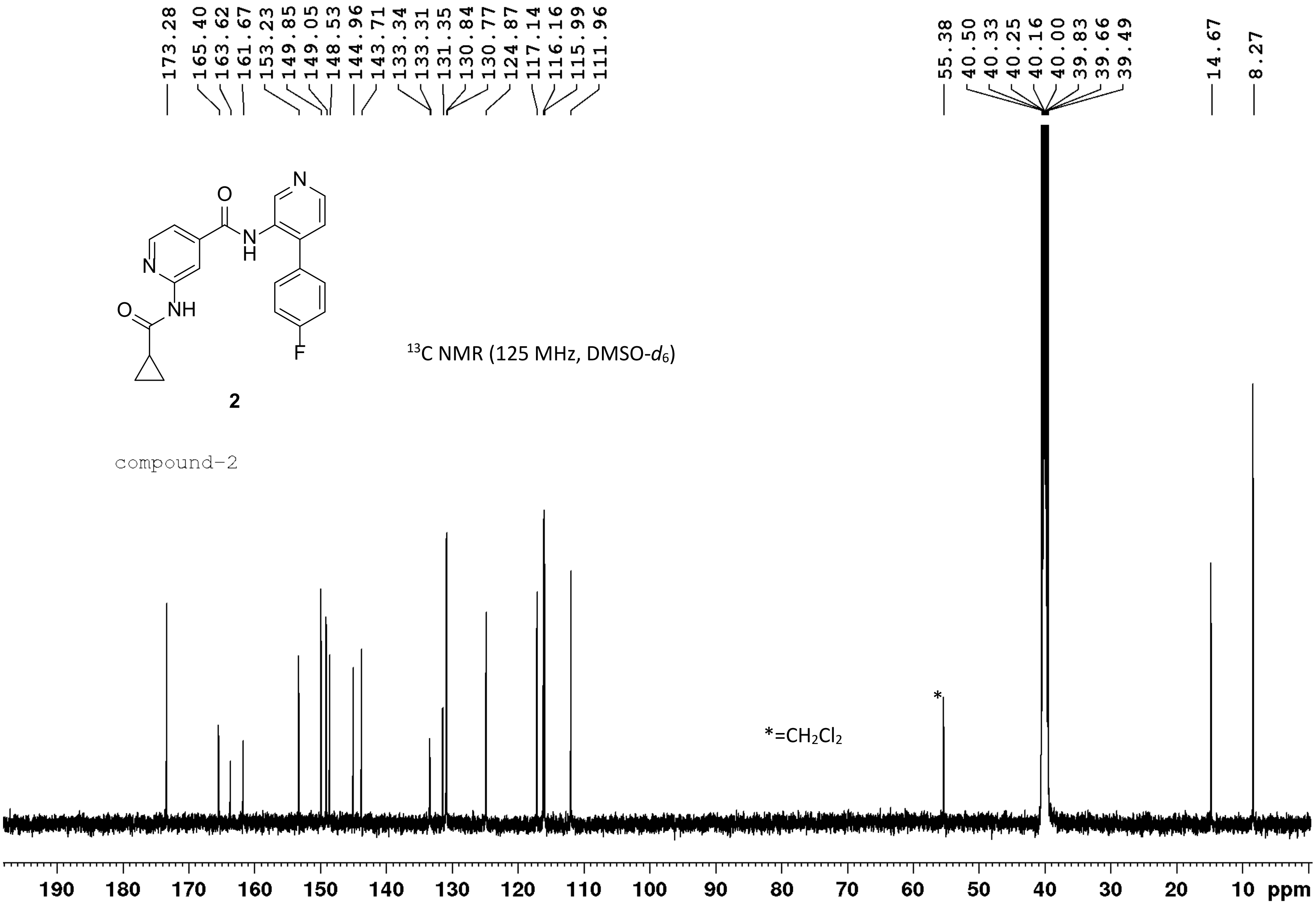
173.28
165.40
163.62
161.67
153.23
149.85
149.05
148.53
144.96
143.71
133.34
133.31
131.35
130.84
130.77
124.87
117.14
116.16
115.99
111.96
55.38
40.50
40.33
40.25
40.16
40.00
39.83
39.66
39.49
14.67
8.27
O
N
H
N
NH
O
F
2
^{13}C NMR (125 MHz, DMSO-d_6)
compound-2
*
*=CH_2Cl_2
190 180 170 160 150 140 130 120 110 100 90 80 70 60 50 40 30 20 10 ppm

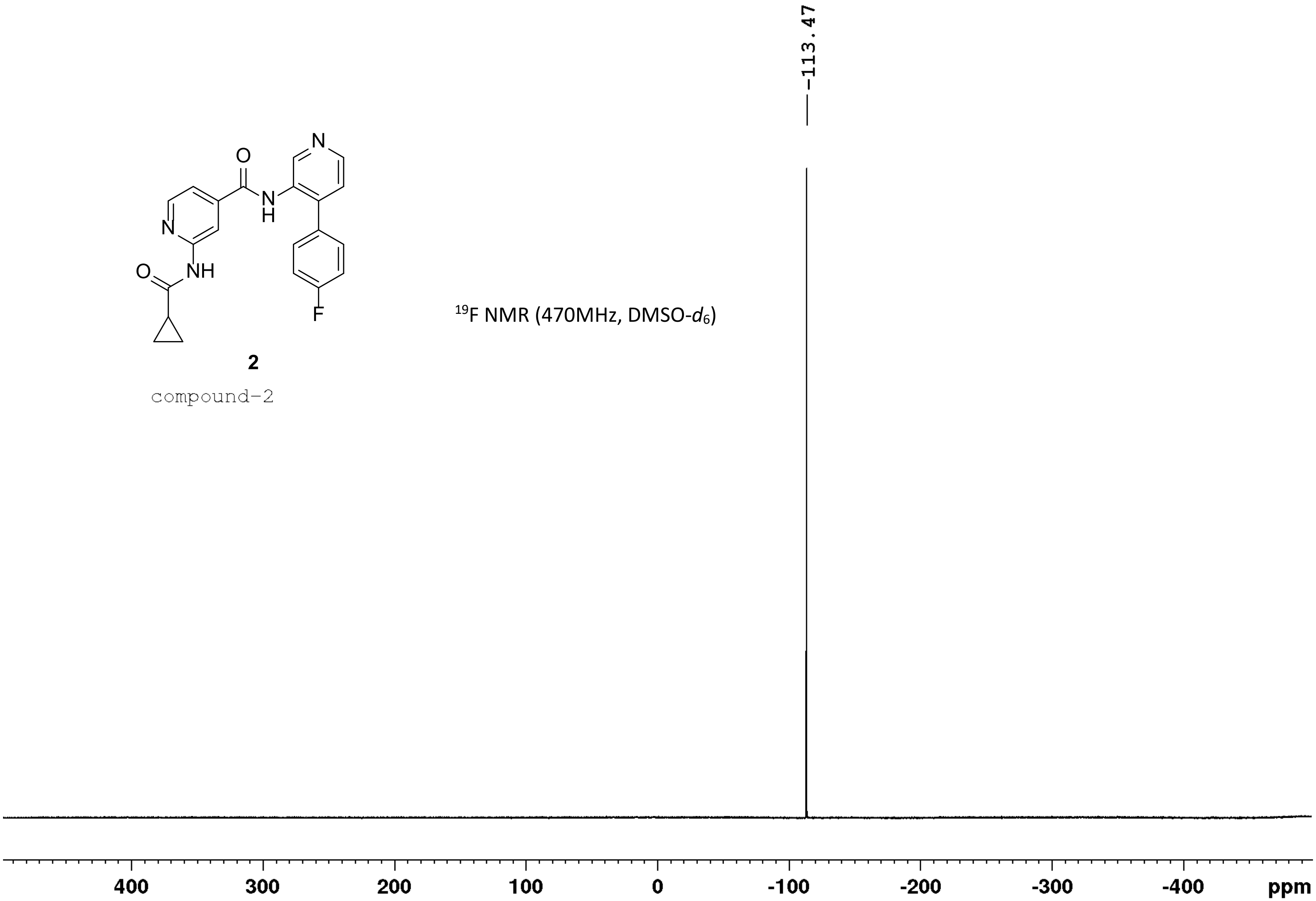
-113.47
O
N
NH
N
H
O
NH
F
2
compound-2
^{19}F NMR (470MHz, DMSO-d_6)
400
300
200
100
0
-100
-200
-300
-400
ppm

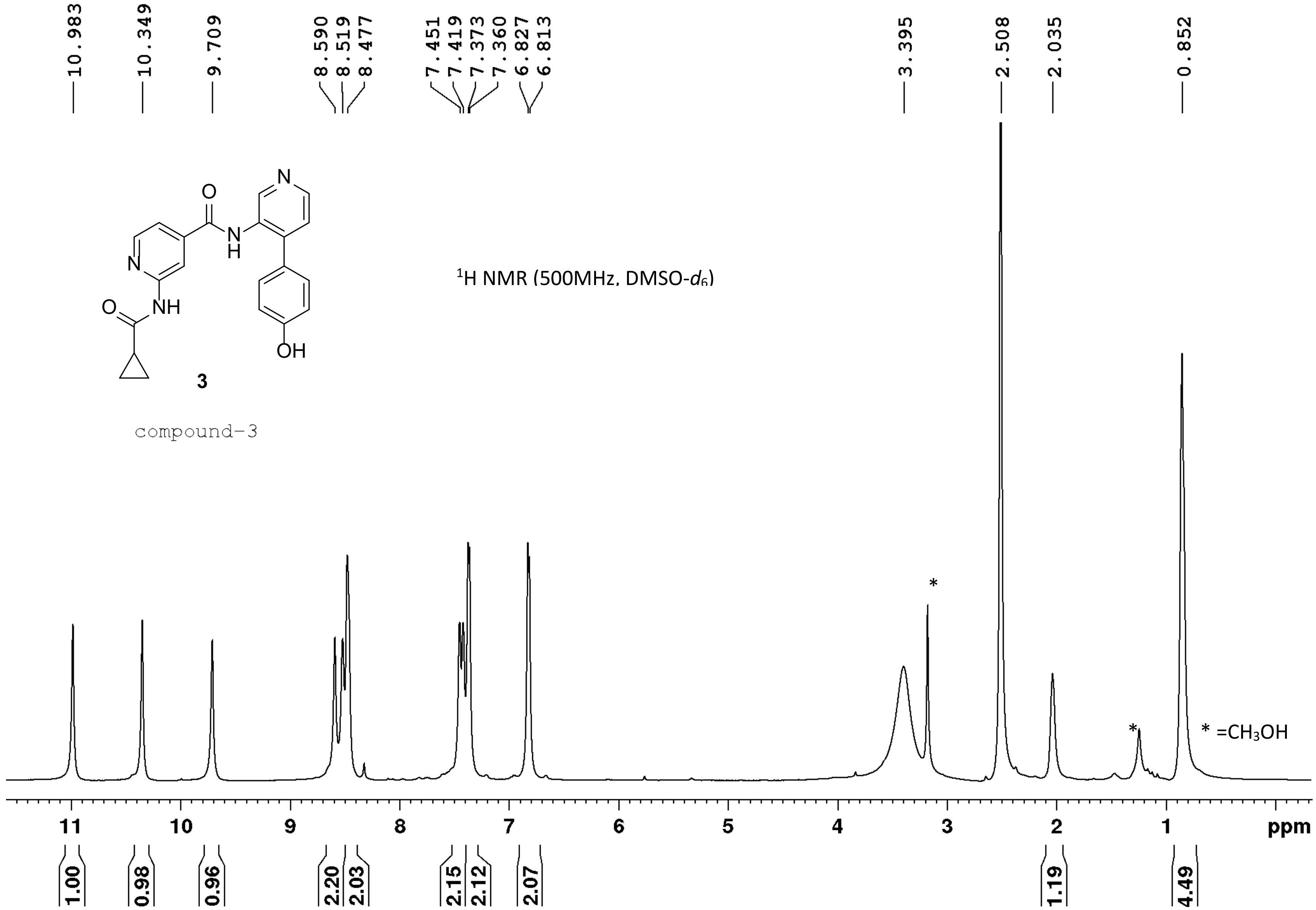
10.983
10.349
9.709
8.590
8.519
8.477
7.451
7.419
7.373
7.360
6.827
6.813
3.395
2.508
2.035
0.852
O
N
NH
N
O
NH
OH
3
compound-3
1H NMR (500MHz, DMSO-d6)
*
* =CH3OH
11
10
9
8
7
6
5
4
3
2
1
ppm
1.00
0.98
0.96
2.20
2.03
2.15
2.12
2.07
1.19
4.49

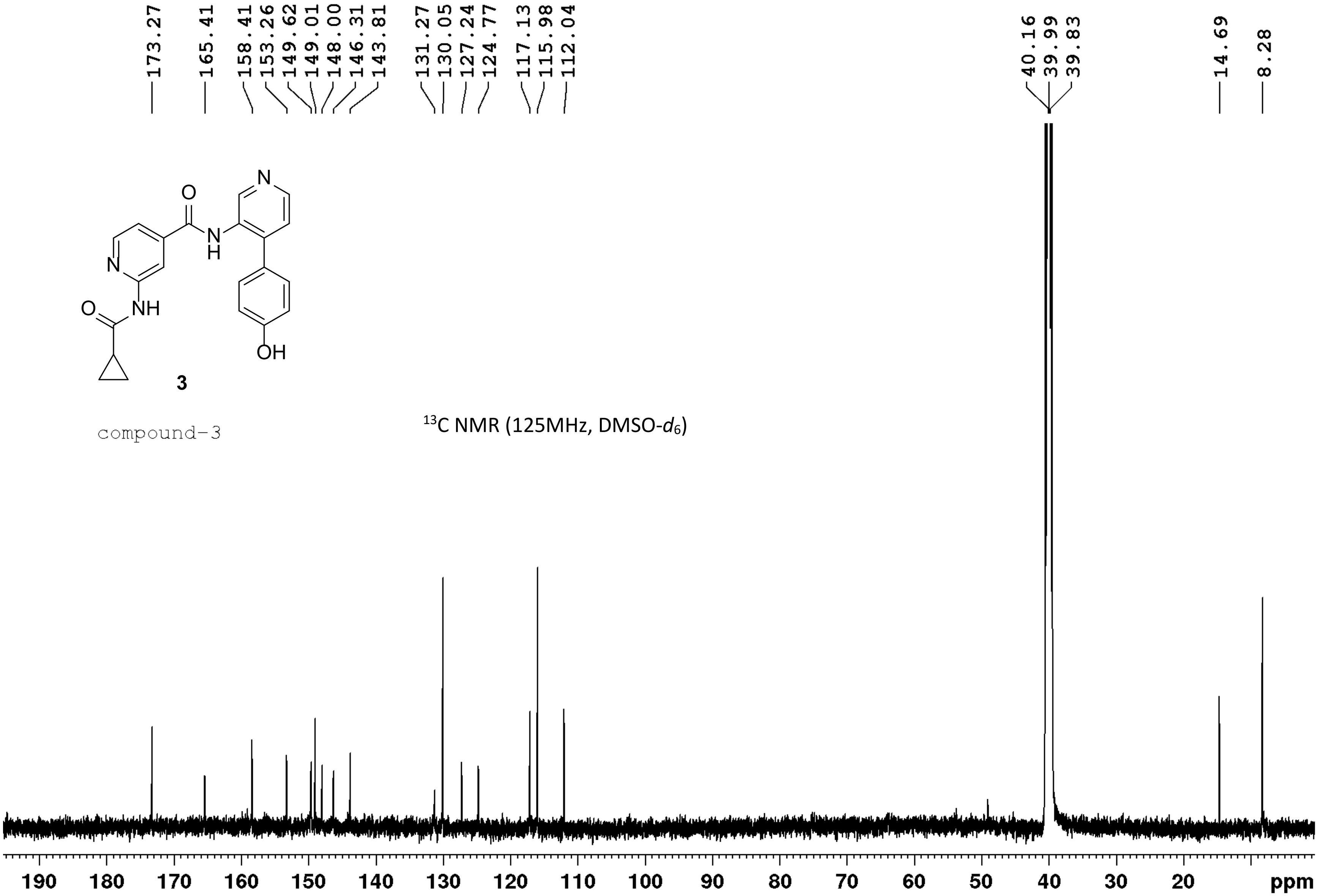

3

compound-3

^{13}C NMR (125MHz, DMSO-d_6)

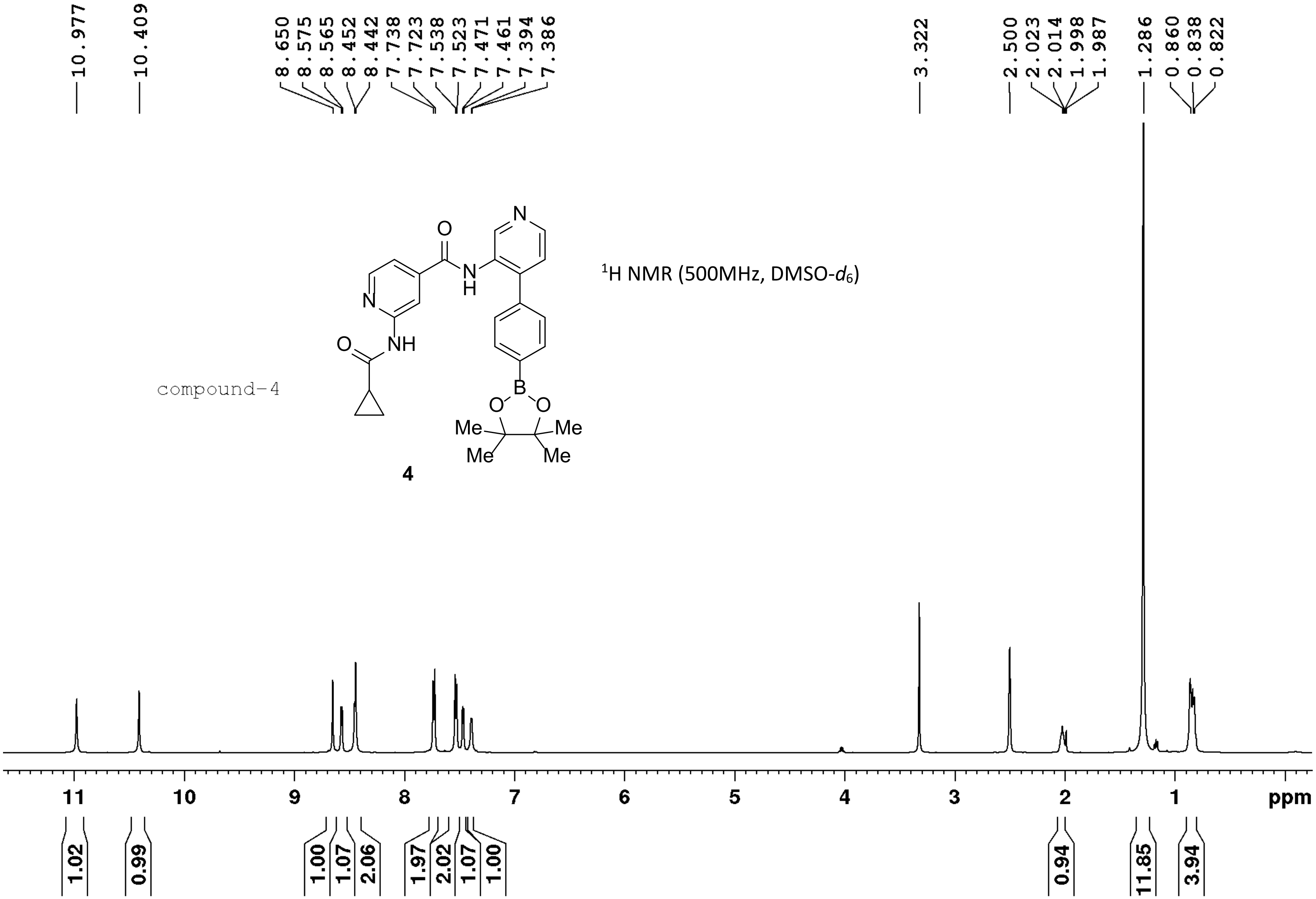
10.977
10.409
8.650
8.575
8.565
8.452
8.442
7.738
7.723
7.538
7.523
7.471
7.461
7.394
7.386
3.322
2.500
2.023
2.014
1.998
1.987
1.286
0.860
0.838
0.822
compound-4
4
^{1}H NMR (500MHz, DMSO-d_6)
11
10
9
8
7
6
5
4
3
2
1
ppm
1.02
0.99
1.00
1.07
2.06
1.97
2.02
1.07
1.00
0.94
11.85
3.94

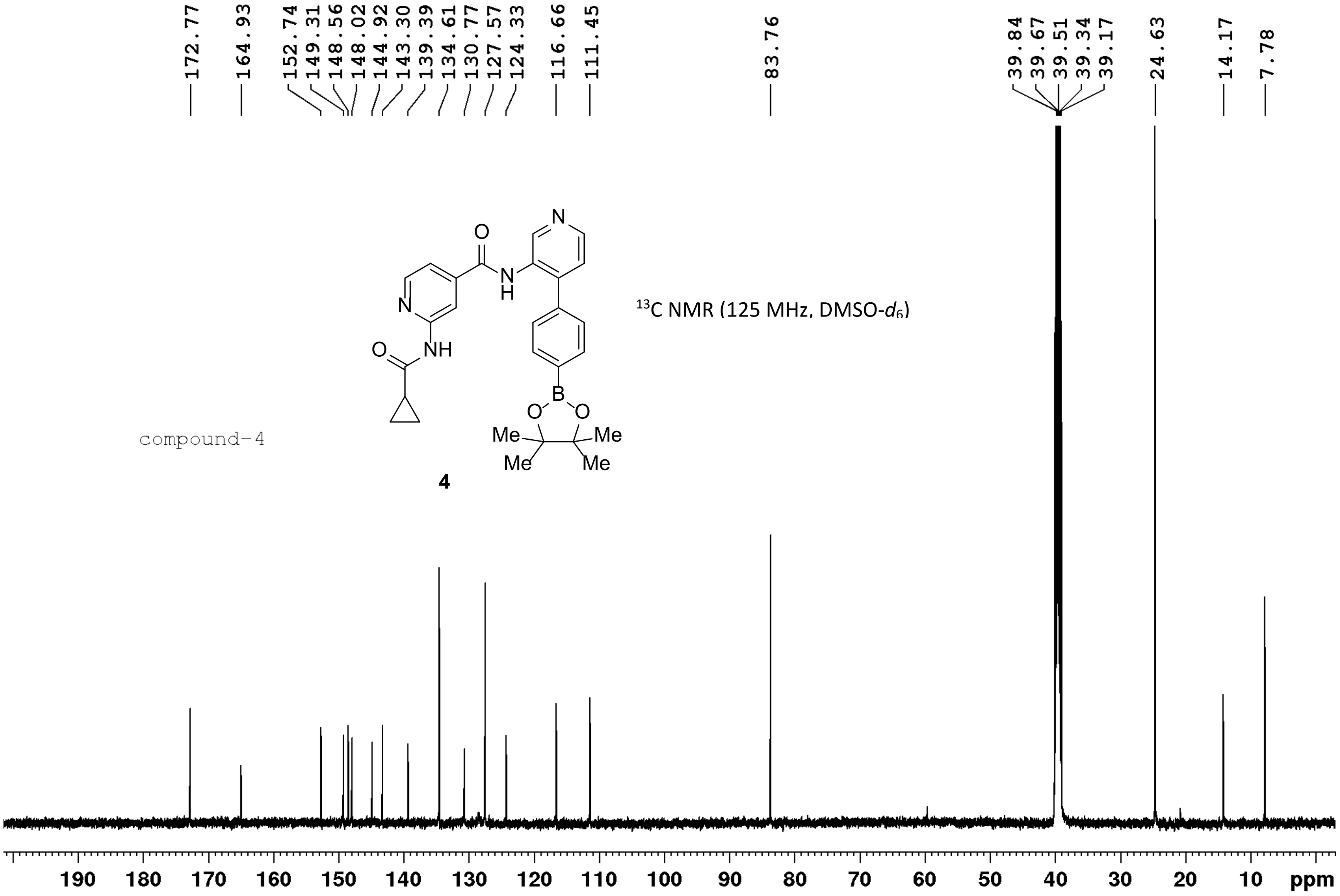
172.77
164.93
152.74
149.31
148.56
148.02
144.92
143.30
139.39
134.61
130.77
127.57
124.33
116.66
111.45
83.76
39.84
39.67
39.51
39.34
39.17
24.63
14.17
7.78
^{13}C NMR (125 MHz, DMSO-d_6)
compound-4
4
190 180 170 160 150 140 130 120 110 100 90 80 70 60 50 40 30 20 10 ppm

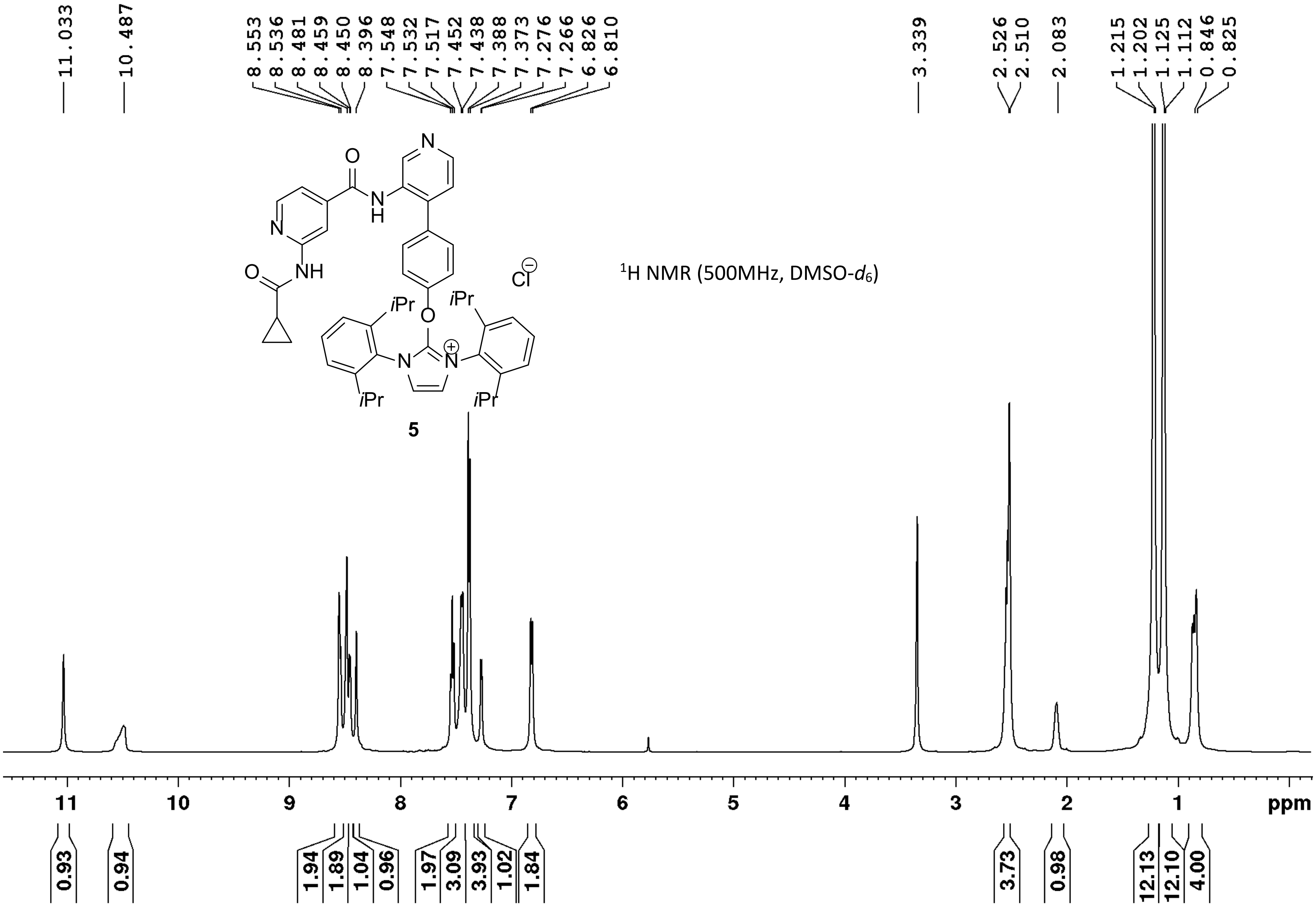
11.033
10.487
8.553
8.536
8.481
8.459
8.450
8.396
7.548
7.532
7.517
7.452
7.438
7.388
7.373
7.276
7.266
6.826
6.810
3.339
2.526
2.510
2.083
1.215
1.202
1.125
1.112
0.846
0.825
O
NH
N
H
N
NH
O
iPr
O
iPr
N
N
iPr
iPr
Cl
5
^{1}H NMR (500MHz, DMSO-d_6)
11
10
9
8
7
6
5
4
3
2
1
ppm
0.93
0.94
1.94
1.89
1.04
0.96
1.97
3.09
3.93
1.02
1.84
3.73
0.98
12.13
12.10
4.00

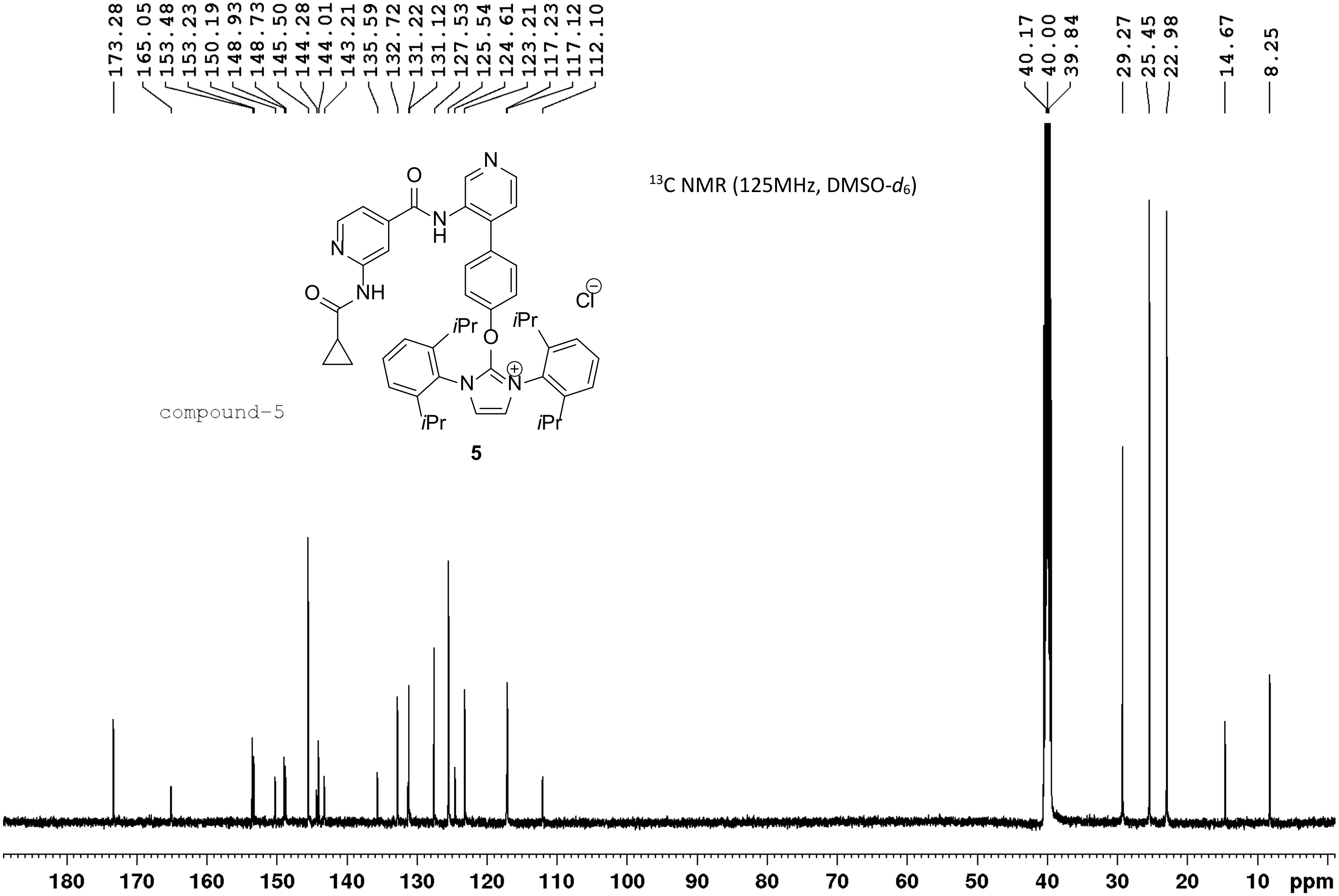
173.28
165.05
153.48
153.23
150.19
148.93
148.73
145.50
144.28
144.01
143.21
135.59
132.72
131.22
131.12
127.53
125.54
124.61
123.21
117.23
117.12
112.10
40.17
40.00
39.84
29.27
25.45
22.98
14.67
8.25
^{13}C NMR (125MHz, DMSO-d_6)
compound-5
5
180 170 160 150 140 130 120 110 100 90 80 70 60 50 40 30 20 10 ppm

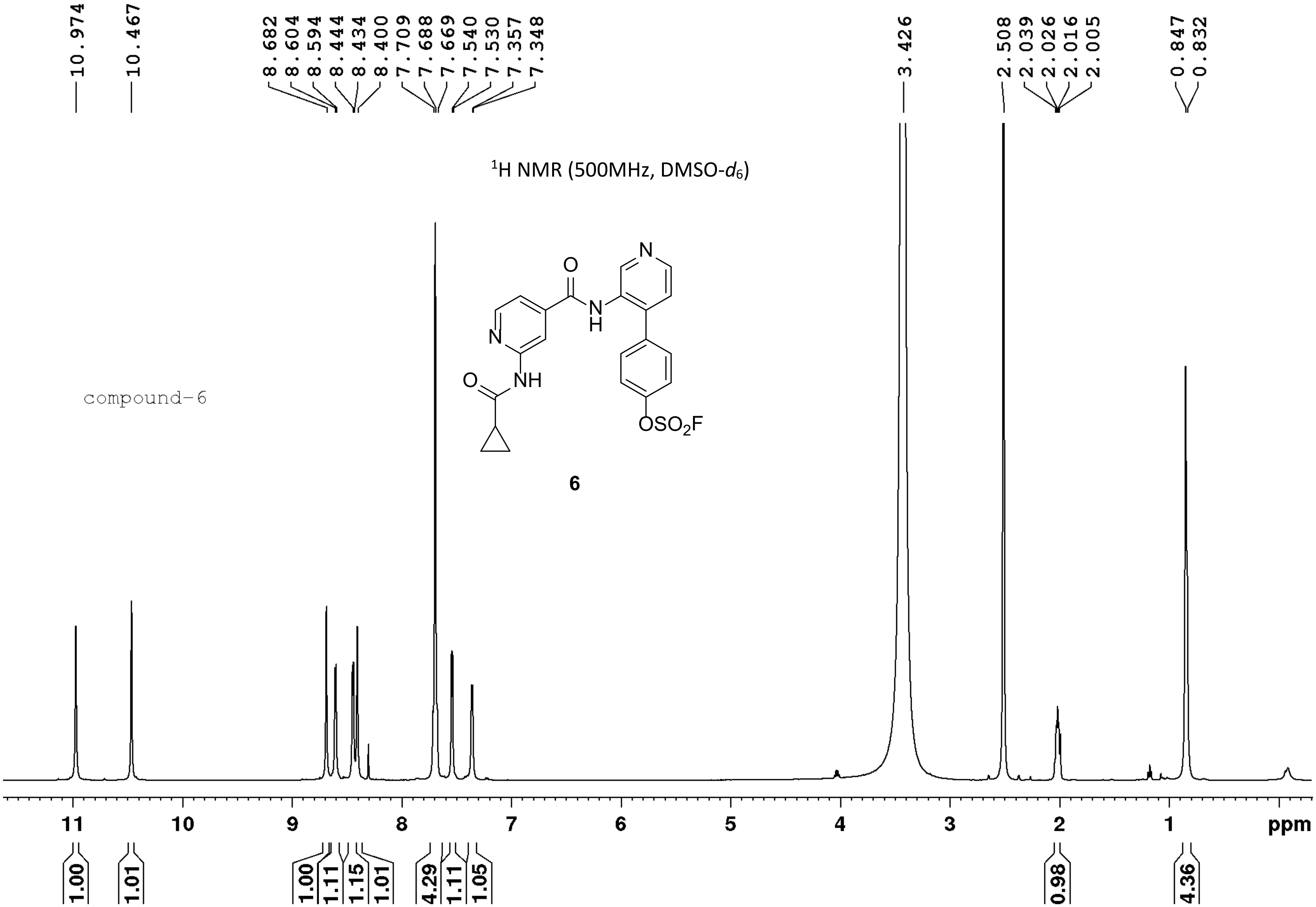
10.974
10.467
8.682
8.604
8.594
8.444
8.434
8.400
7.709
7.688
7.669
7.540
7.530
7.357
7.348
3.426
2.508
2.039
2.026
2.016
2.005
0.847
0.832
1H NMR (500MHz, DMSO-d6)
compound-6
O
N
NH
H
N
O
NH
OSO2F
6
11
10
9
8
7
6
5
4
3
2
1
ppm
1.00
1.01
1.00
1.11
1.15
1.01
4.29
1.11
1.05
0.98
4.36

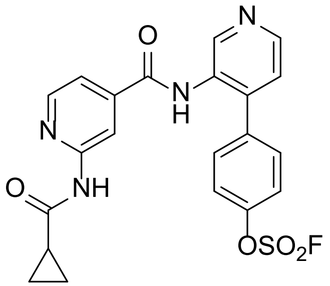

6

^{13}C NMR (125 MHz, DMSO-d_6)

compound-6

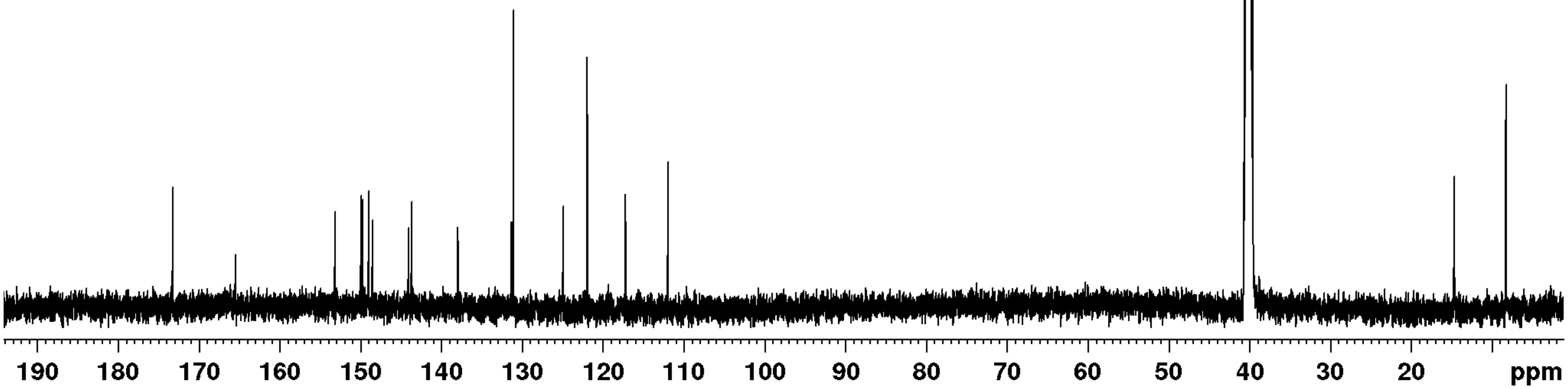

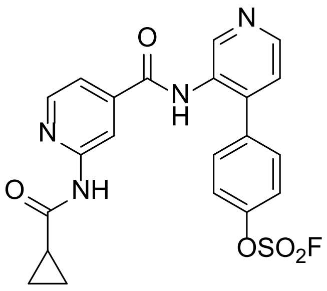

6

compound-6

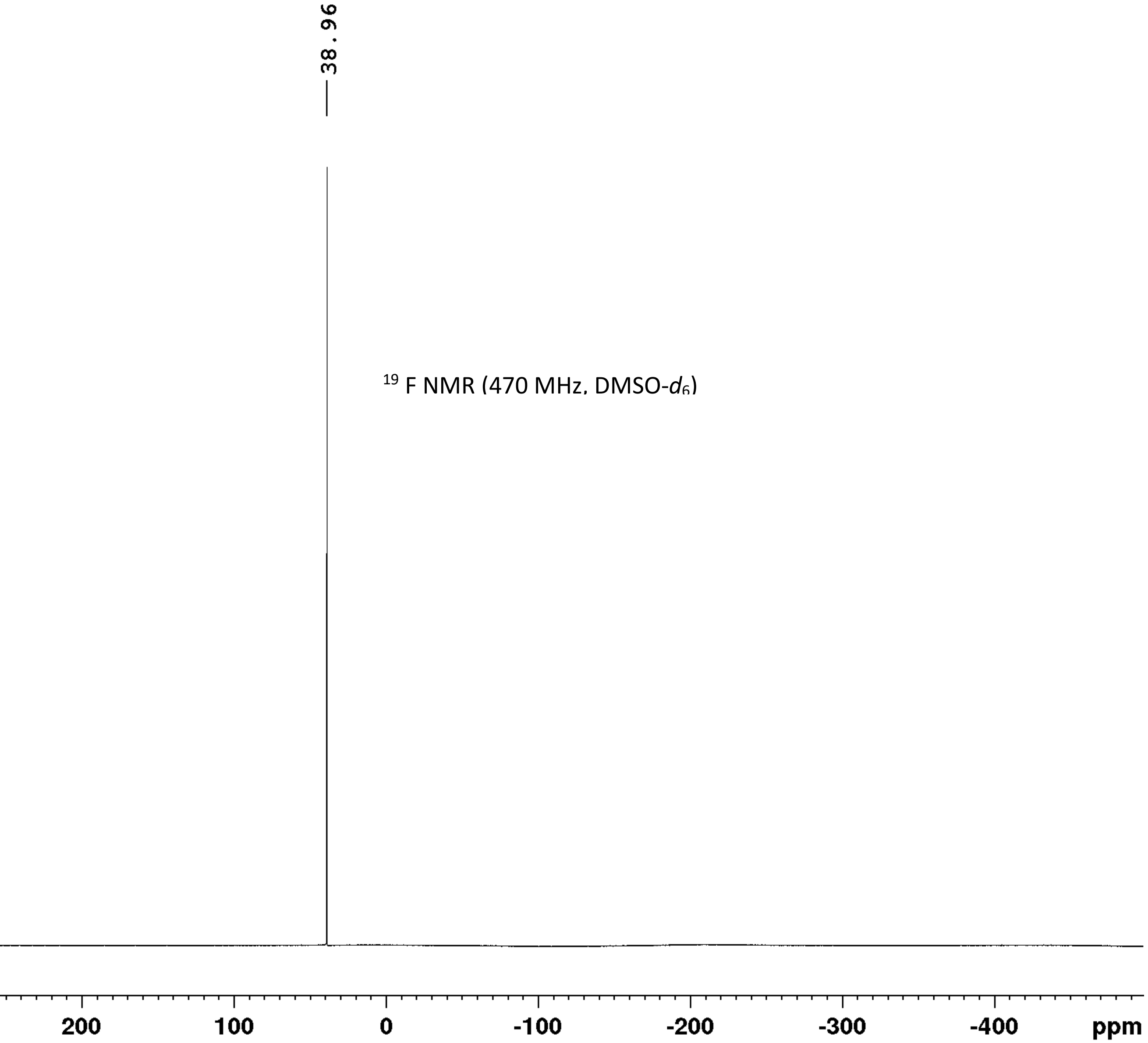
38.96
^{19}F NMR (470 MHz, DMSO-d_6)
400
300
200
100
0
-100
-200
-300
-400
ppm

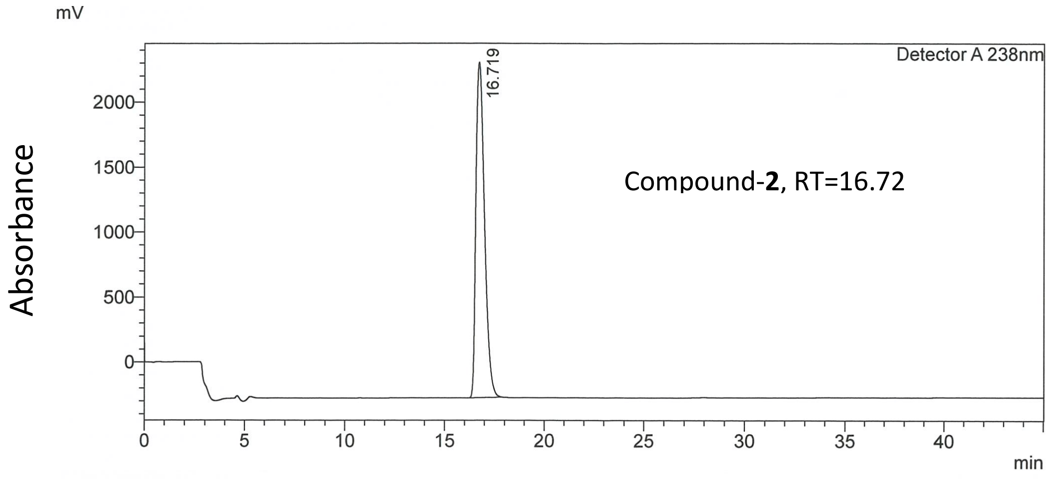

Peak#	Name	Ret. Time	Area	Height	Area%
1		16.719	77387194	2578236	100.000
Total			77387194	2578236	100.000

AD2

Peak#	Name	Ret. Time	Area	Height	Area%
Total					

Supplementary Figure S1: Representative analytical HPLC trace of compound **2.**

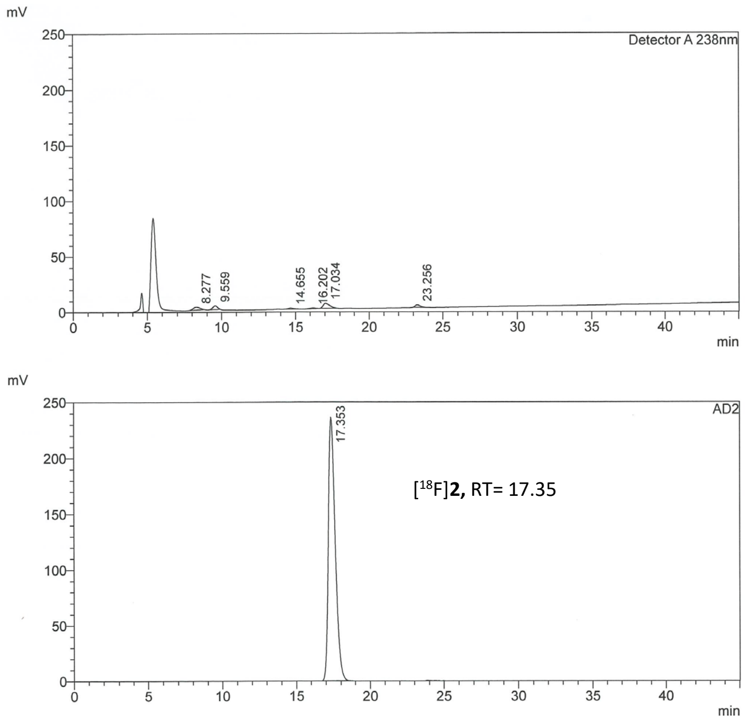

Peak#	Name	Ret. Time	Area	Height	Area%
1		8.277	86829	2679	21.873
2		9.559	75613	3435	19.048
3		14.655	25698	964	6.474
4		16.202	20562	681	5.180
5		17.034	137060	4597	34.527
6		23.256	51201	2228	12.898
Total			396963	14584	100.000

AD2

Peak#	Name	Ret. Time	Area	Height	Area%
1		17.353	7521580	236160	100.000
Total			7521580	236160	100.000

Supplementary Figure S2: Representative analytical HPLC trace of post semi preparative column purified [^{18}F]**2.**

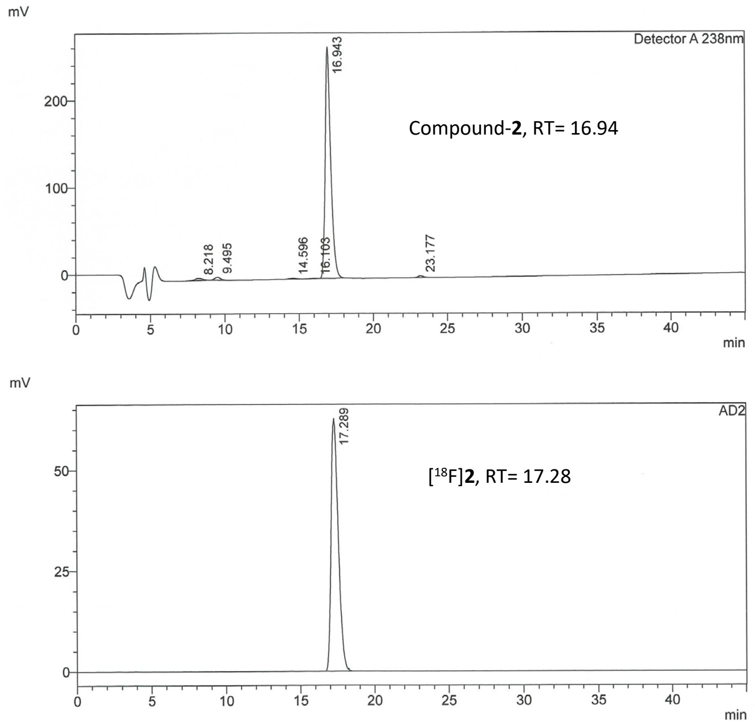

Peak#	Name	Ret. Time	Area	Height	Area%
1		8.218	85918	2438	1.201
2		9.495	90144	3349	1.260
3		14.596	21835	837	0.305
4		16.103	6308	252	0.088
5		16.943	6894483	265349	96.354
6		23.177	56661	2177	0.792
Total			7155349	274402	100.000

AD2

Peak#	Name	Ret. Time	Area	Height	Area%
1		17.289	2082501	62786	100.000
Total			2082501	62786	100.000

Supplementary Figure S3: Representative analytical HPLC trace of post semi preparative column purified [^{18}F]**2** with coinjection compound **2.**

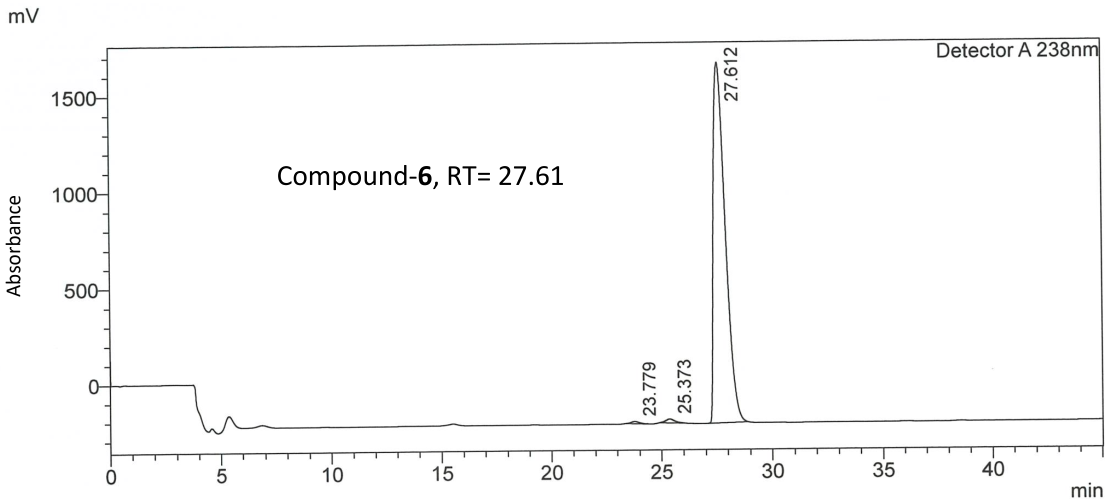

Peak#	Name	Ret. Time	Area	Height	Area%
1		23.779	292772	11541	0.416
2		25.373	581385	20286	0.826
3		27.612	69547590	1874885	98.759
Total			70421747	1906712	100.000

AD2

Peak#	Name	Ret. Time	Area	Height	Area%
Total					

Supplementary Figure S4: Representative analytical HPLC trace of compound **6.**

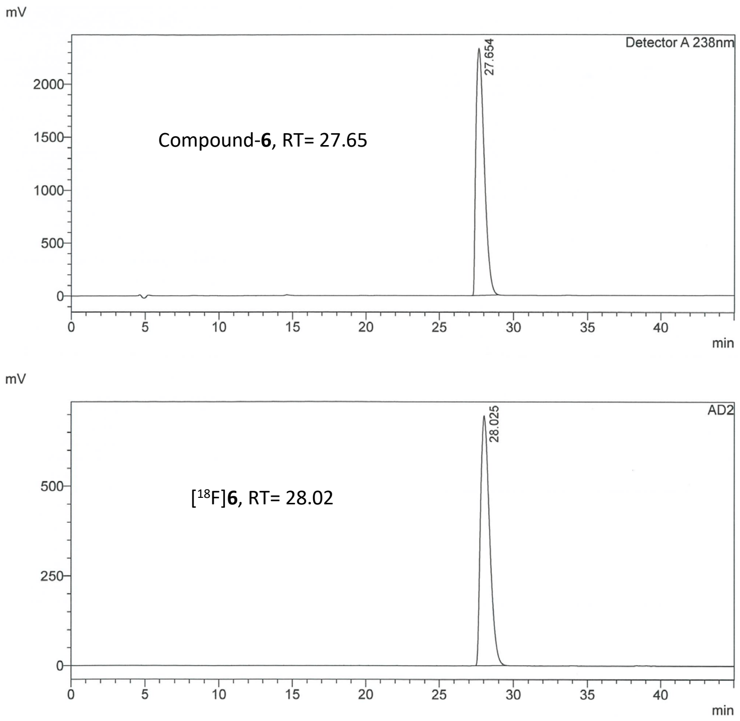

Peak#	Name	Ret. Time	Area	Height	Area%
1		27.654	90645800	2326882	100.000
Total			90645800	2326882	100.000

AD2

Peak#	Name	Ret. Time	Area	Height	Area%
1		28.025	29663446	696187	100.000
Total			29663446	696187	100.000

Supplementary Figure S5: Representative analytical HPLC trace of post semi preparative column purified [^{18}F]**6.**

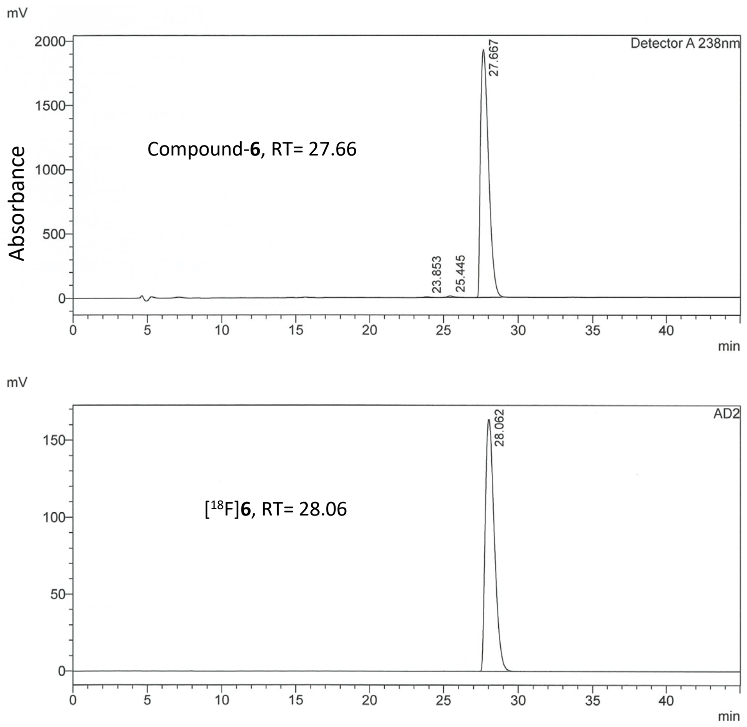

Peak#	Name	Ret. Time	Area	Height	Area%
1		23.853	136647	5945	0.190
2		25.445	342958	11725	0.476
3		27.667	71625828	1926958	99.335
Total			72105432	1944628	100.000

AD2

Peak#	Name	Ret. Time	Area	Height	Area%
1		28.062	6878861	163446	100.000
Total			6878861	163446	100.000

Supplementary Figure S6: Representative analytical HPLC trace of post semi preparative column purified [^{18}F]**6** with coinjection compound **6.**

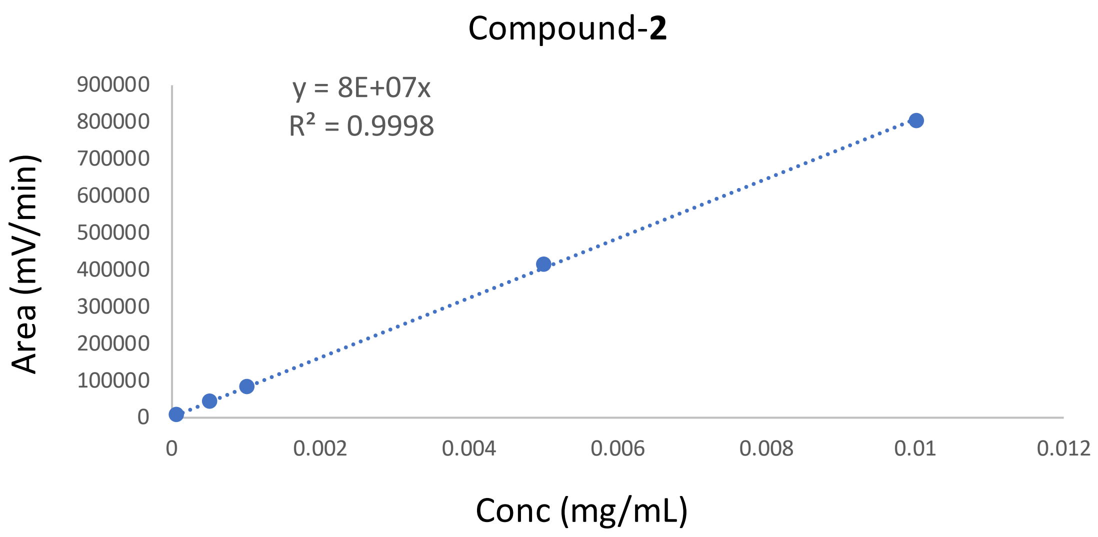

Supplementary Figure S7: Calibration curve of compound **2** used for molar activity calculation**.**

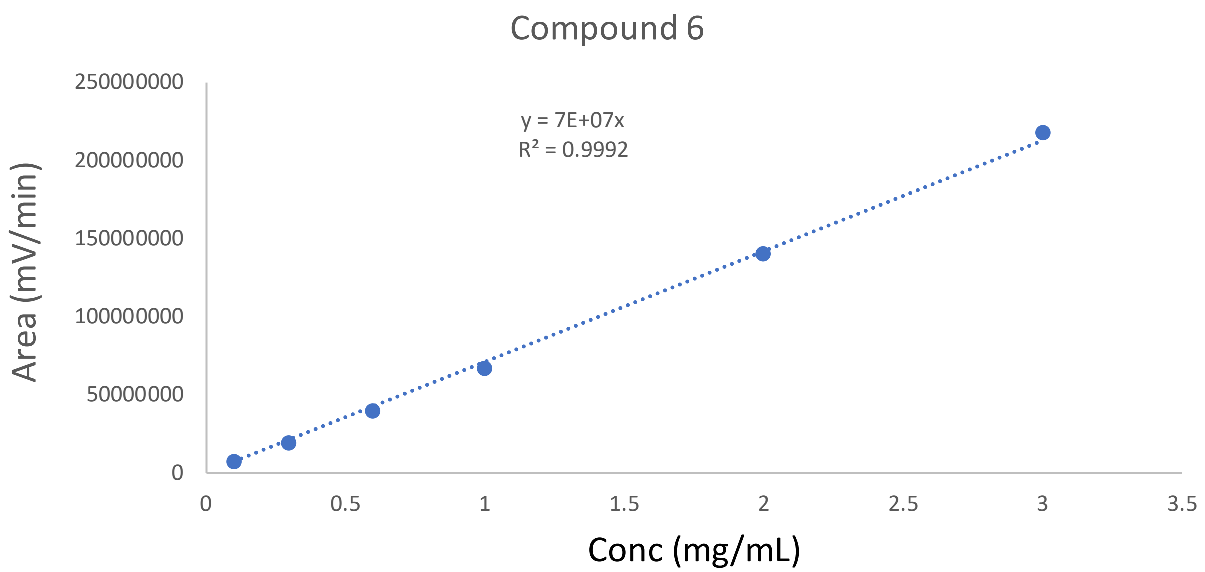

Supplementary Figure S8: Calibration curve of compound **6** used for molar activity calculation**.**

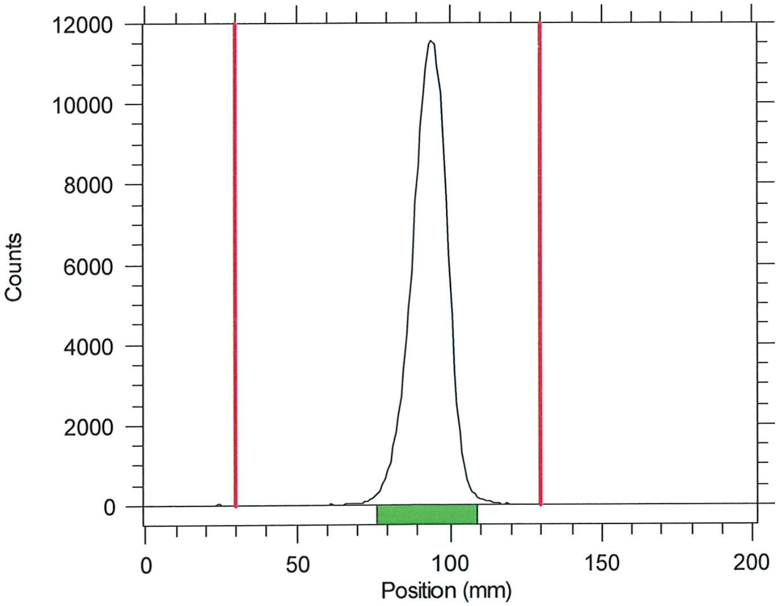

Radio-TLC of [^{18}F]**2** in Methanol/$CHCl_3$ (0.5:9.5).

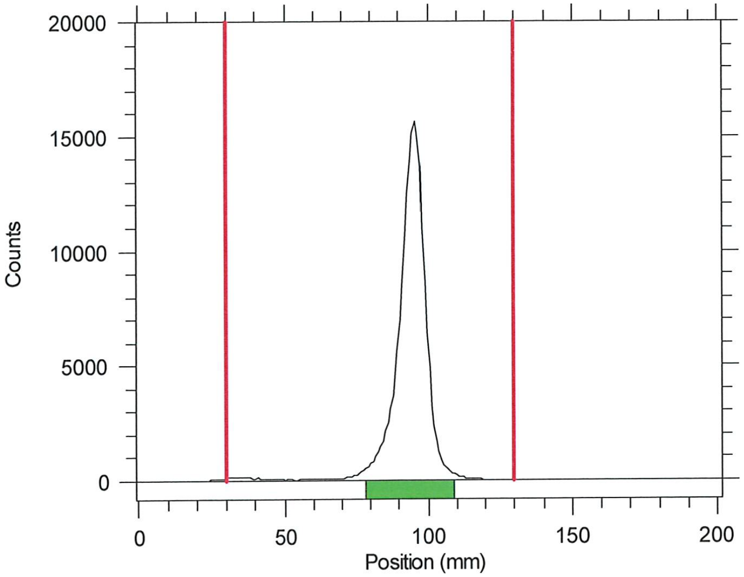

Radio-TLC of [^{18}F]**6** in Methanol/$CHCl_3$ (0.5:9.5).

Supplementary Figure S9: Radio-TLC of [^{18}F]**2** and [^{18}F]**6.**

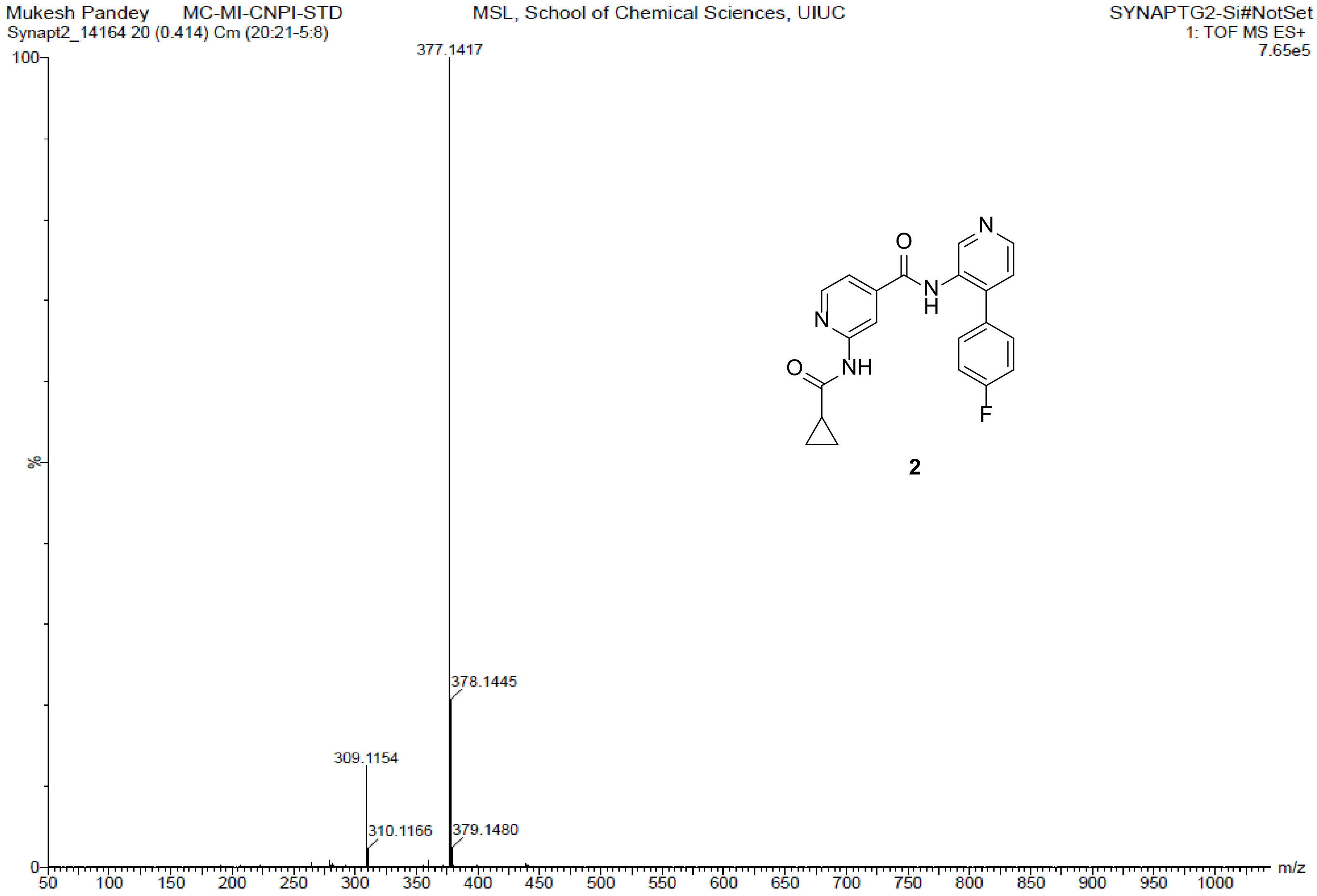
Mukesh Pandey MC-MI-CNPI-STD
MSL, School of Chemical Sciences, UIUC
SYNAPTG2-Si#NotSet
Synapt2_14164 20 (0.414) Cm (20:21-5:8)
1: TOF MS ES+
7.65e5
377.1417
378.1445
309.1154
310.1166
379.1480
100
%
0
m/z
50
100
150
200
250
300
350
400
450
500
550
600
650
700
750
800
850
900
950
1000
O
N
H
N
NH
O
F
2

Elemental Composition Report

Single Mass Analysis

Tolerance = 5.0 PPM / DBE: min = -1.5, max = 200.0
Element prediction: Off
Number of isotope peaks used for i-FIT = 9

Monoisotopic Mass, Even Electron Ions
159 formula(e) evaluated with 1 results within limits (up to 50 closest results for each mass)
Elements Used:
C: 0-50 H: 0-100 N: 0-5 O: 0-5 F: 1-1

Mukesh Pandey MC-MI-CNPI-STD MSL, School of Chemical Sciences, UIUC SYNAPTG2-Si#NotSet
Synapt2_14164 20 (0.414) Cm (20:21-5:8) 1: TOF MS ES+
7.65e+005

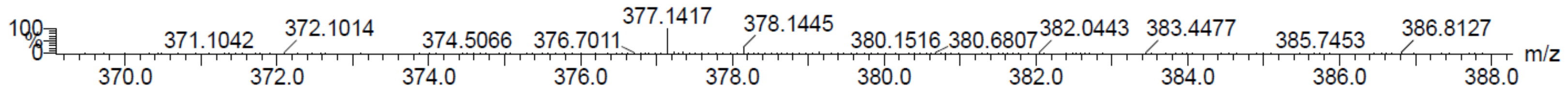

Minimum:				-1.5				
Maximum:		5.0	5.0	200.0				
Mass	Calc. Mass	mDa	PPM	DBE	i-FIT	Norm	Conf(%)	Formula
377.1417	377.1414	0.3	0.8	14.5	819.0	n/a	n/a	C21 H18 N4 O2 F

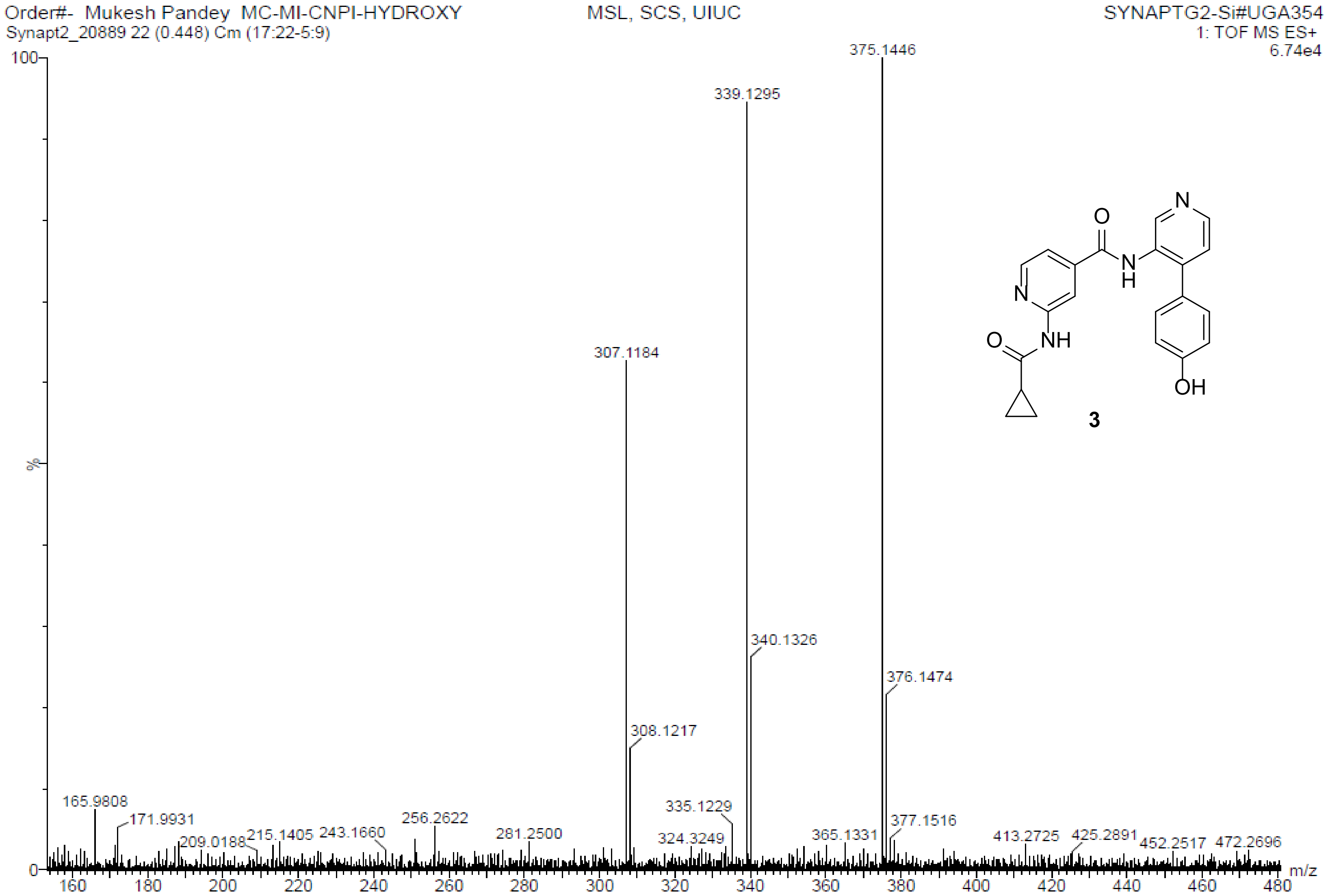
Order#- Mukesh Pandey MC-MI-CNPI-HYDROXY
MSL, SCS, UIUC
SYNAPTG2-Si#UGA354
Synapt2_20889 22 (0.448) Cm (17:22-5:9)
1: TOF MS ES+
6.74e4
100
%
0
375.1446
339.1295
307.1184
340.1326
376.1474
308.1217
165.9808
171.9931
209.0188
215.1405
243.1660
256.2622
281.2500
335.1229
324.3249
365.1331
377.1516
413.2725
425.2891
452.2517
472.2696
160
180
200
220
240
260
280
300
320
340
360
380
400
420
440
460
480
m/z
O
N
N
H
N
O
NH
OH
3

Elemental Composition Report

Single Mass Analysis

Tolerance = 5.0 PPM / DBE: min = -1.5, max = 200.0
Element prediction: Off
Number of isotope peaks used for i-FIT = 9

Monoisotopic Mass, Even Electron Ions
170 formula(e) evaluated with 1 results within limits (up to 50 best isotopic matches for each mass)
Elements Used:
C: 0-50 H: 0-60 N: 0-5 O: 0-5

Order#- Mukesh Pandey MC-MI-CNPI-HYDROXY MSL, SCS, UIUC SYNAPTG2-Si#UGA354
Synapt2_20889 22 (0.448) Cm (17:22-5:9) 1: TOF MS ES+
6.74e+004

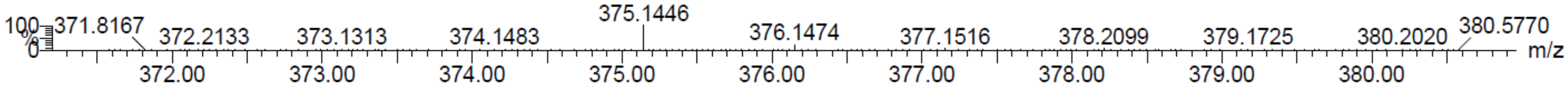

Minimum:				-1.5				
Maximum:		5.0	5.0	200.0				

Mass	Calc. Mass	mDa	PPM	DBE	i-FIT	Norm	Conf(%)	Formula
375.1446	375.1457	-1.1	-2.9	14.5	1019.7	n/a	n/a	C21 H19 N4 O3

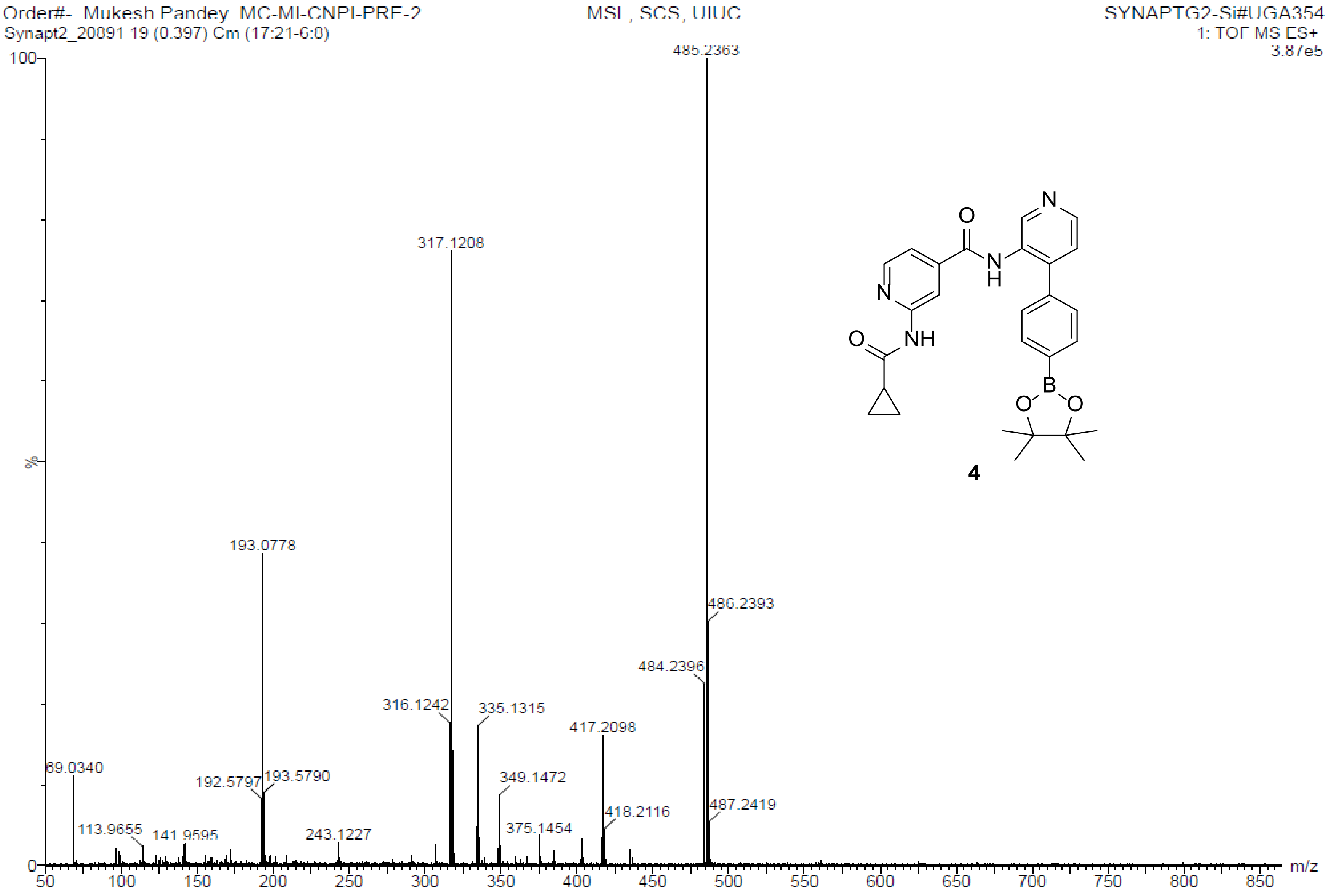
Order#- Mukesh Pandey MC-MI-CNPI-PRE-2
MSL, SCS, UIUC
SYNAPTG2-Si#UGA354
Synapt2_20891 19 (0.397) Cm (17:21-6:8)
1: TOF MS ES+
3.87e5
100
%
0
485.2363
317.1208
193.0778
486.2393
484.2396
316.1242
335.1315
417.2098
69.0340
192.5797
193.5790
349.1472
487.2419
418.2116
113.9655
141.9595
243.1227
375.1454
50
100
150
200
250
300
350
400
450
500
550
600
650
700
750
800
850
m/z
O
N
N
H
N
O
NH
B
O
O
4

Elemental Composition Report

Single Mass Analysis

Tolerance = 5.0 PPM / DBE: min = -1.5, max = 200.0
Element prediction: Off
Number of isotope peaks used for i-FIT = 9

Monoisotopic Mass, Even Electron Ions
186 formula(e) evaluated with 1 results within limits (up to 50 best isotopic matches for each mass)
Elements Used:
C: 0-50 H: 0-60 N: 0-5 O: 0-5 B: 1-1

Order#- Mukesh Pandey MC-MI-CNPI-PRE-2 MSL, SCS, UIUC SYNAPTG2-Si#UGA354
Synapt2_20891 19 (0.397) Cm (17:21-6:8) 1: TOF MS ES+
3.87e+005

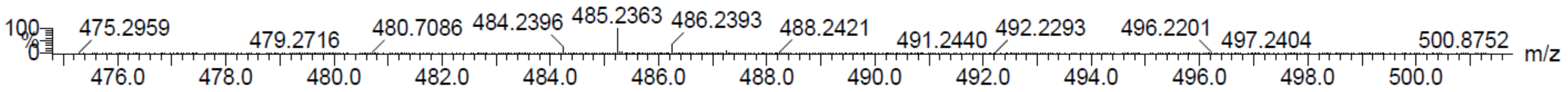

Minimum:				-1.5				
Maximum:		5.0	5.0	200.0				
Mass	Calc. Mass	mDa	PPM	DBE	i-FIT	Norm	Conf(%)	Formula
485.2363	485.2360	0.3	0.6	15.5	2029.0	n/a	n/a	C27 H30 N4 O4 B

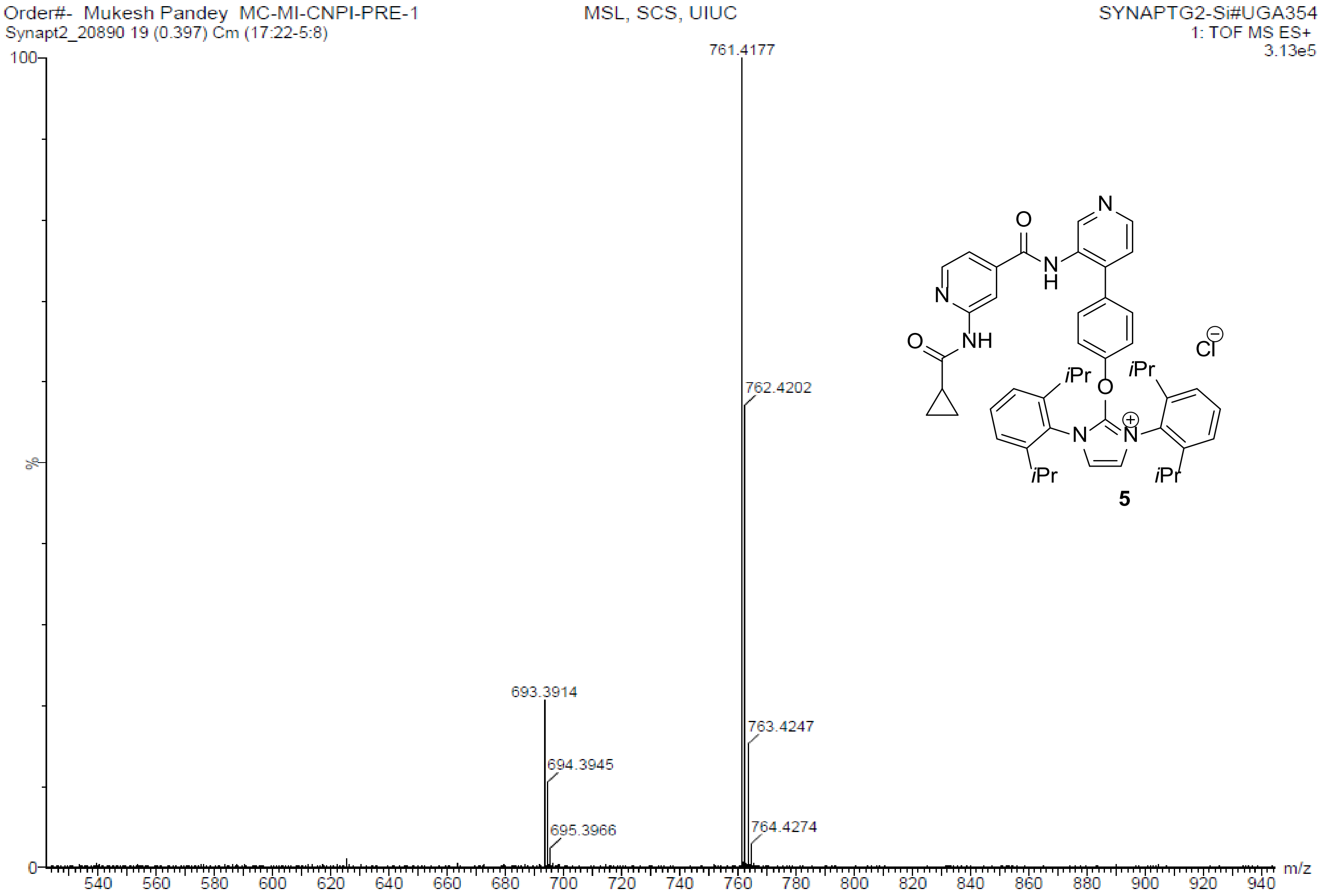
Order#- Mukesh Pandey MC-MI-CNPI-PRE-1
MSL, SCS, UIUC
SYNAPTG2-Si#UGA354
Synapt2_20890 19 (0.397) Cm (17:22-5:8)
1: TOF MS ES+
3.13e5
761.4177
762.4202
763.4247
764.4274
693.3914
694.3945
695.3966
100
%
0
m/z
540
560
580
600
620
640
660
680
700
720
740
760
780
800
820
840
860
880
900
920
940
5

Elemental Composition Report

Single Mass Analysis

Tolerance = 10.0 PPM / DBE: min = -1.5, max = 200.0
Element prediction: Off
Number of isotope peaks used for i-FIT = 9

Monoisotopic Mass, Even Electron Ions
58 formula(e) evaluated with 1 results within limits (up to 50 best isotopic matches for each mass)
Elements Used:
C: 0-50 H: 0-60 N: 1-7 O: 1-5

Order# - Mukesh Pandey MC-MI-CNPI-PRE-1 MSL, SCS, UIUC SYNAPTG2-Si#UGA354
Synapt2_20890 19 (0.397) Cm (17:22-5:8) 1: TOF MS ES+
3.13e+005

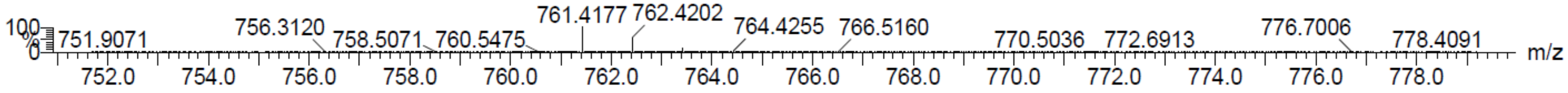

Mass	Calc. Mass	mDa	PPM	DBE	i-FIT	Norm	Conf(%)	Formula
Minimum:				-1.5				
Maximum:		5.0	10.0	200.0				
761.4177	761.4179	-0.2	-0.3	25.5	3437.0	n/a	n/a	C48 H53 N6 O3

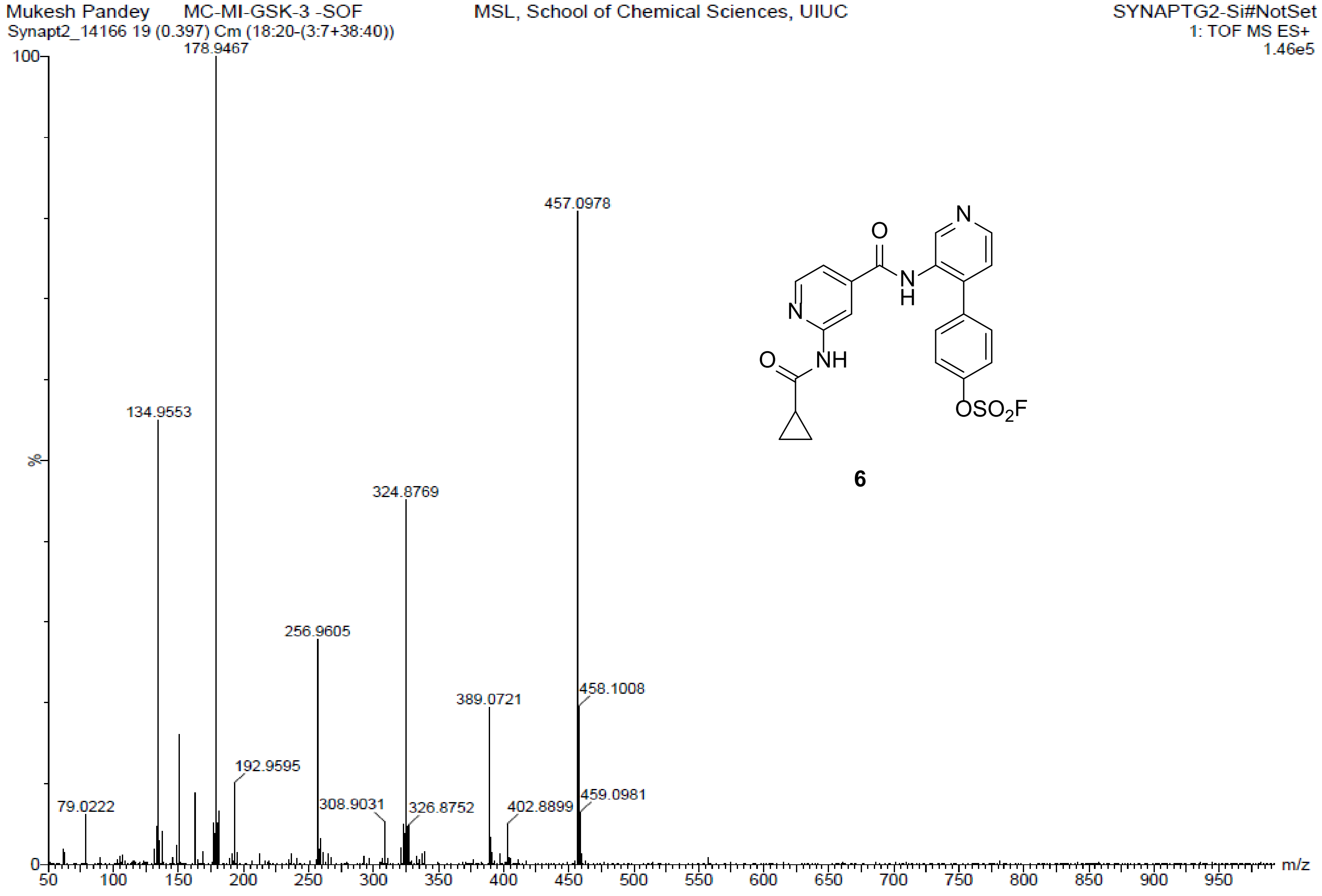
Mukesh Pandey MC-MI-GSK-3 -SOF
MSL, School of Chemical Sciences, UIUC
SYNAPTG2-Si#NotSet
Synapt2_14166 19 (0.397) Cm (18:20-(3:7+38:40))
1: TOF MS ES+
1.46e5
100
%
0
178.9467
457.0978
134.9553
324.8769
256.9605
389.0721
458.1008
192.9595
79.0222
308.9031
326.8752
402.8899
459.0981
50
100
150
200
250
300
350
400
450
500
550
600
650
700
750
800
850
900
950
m/z
O
N
N
H
N
O
NH
OSO2F
6

Elemental Composition Report

Single Mass Analysis

Tolerance = 5.0 PPM / DBE: min = -1.5, max = 200.0
Element prediction: Off
Number of isotope peaks used for i-FIT = 9

Monoisotopic Mass, Even Electron Ions
348 formula(e) evaluated with 2 results within limits (up to 50 closest results for each mass)
Elements Used:
C: 0-50 H: 0-100 N: 0-5 O: 0-5 F: 1-1 S: 1-2

Mukesh Pandey MC-MI-GSK-3 -SOF MSL, School of Chemical Sciences, UIUC SYNAPTG2-Si#NotSet
Synapt2_14166 19 (0.397) Cm (18:20-(3:7+38:40)) 1: TOF MS ES+
1.18e+005

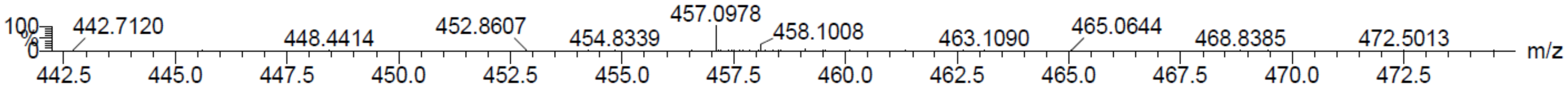

Minimum:				-1.5				
Maximum:		5.0	5.0	200.0				

Mass	Calc. Mass	mDa	PPM	DBE	i-FIT	Norm	Conf(%)	Formula
457.0978	457.0982	-0.4	-0.9	14.5	226.0	0.009	99.15	$C_{21} H_{18} N_{4} O_{5} F S$
	457.0957	2.1	4.6	18.5	230.7	4.762	0.85	$C_{25} H_{18} N_{4} F S_{2}$

Time (min)	Percentage of intact [^{18}F]**2**			Percentage of intact [^{18}F]**6**		
	In isotonic NaCl solution (%)	In mouse serum (%)	In human serum (%)	In isotonic NaCl solution (%)	in mouse serum (%)	human serum (%)
0 (n=3)	100 ± 0.0	100 ± 0.0	100 ± 0.0	100 ± 0.0	100 ± 0.0	100 ± 0.0
30 (n=3)	100 ± 0.0	100 ± 0.0	100 ± 0.0	100 ± 0.0	87.30±8.99	92.46±2.61
60 (n=3)	100 ± 0.0	100 ± 0.0	100 ± 0.0	100 ± 0.0	81.17±6.38	80.13±14.24
120 (n=3)	100 ± 0.0	100 ± 0.0	100 ± 0.0	100 ± 0.0	67.01±4.93	66.20±6.38

Supplementary Table S1. Average stability of [^{18}F]**2** and [^{18}F]**6** in isotonic NaCl solution, mouse, and human serums over time.

Reg	(mm) Start	(mm) Stop	(mm) Centroid	RF	Region Counts	Region CPM	% of Total	% of ROI
Rgn 1	91.9	119.2	106.9	0.699	97885.0	172738.2	97.70	100.00
1 Peaks					97885.0	172738.2	97.70	100.00

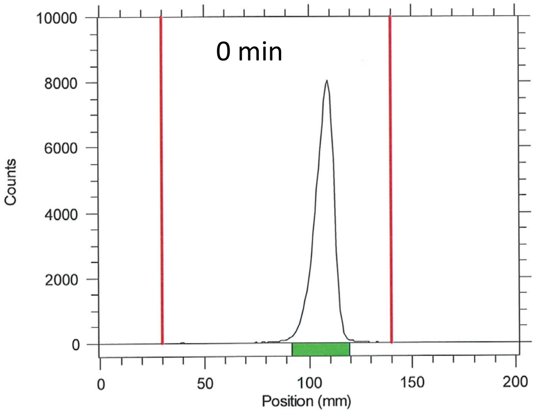

Reg	(mm) Start	(mm) Stop	(mm) Centroid	RF	Region Counts	Region CPM	% of Total	% of ROI
Rgn 1	89.4	120.0	105.9	0.690	81555.0	47507.8	97.08	100.00
1 Peaks					81555.0	47507.8	97.08	100.00

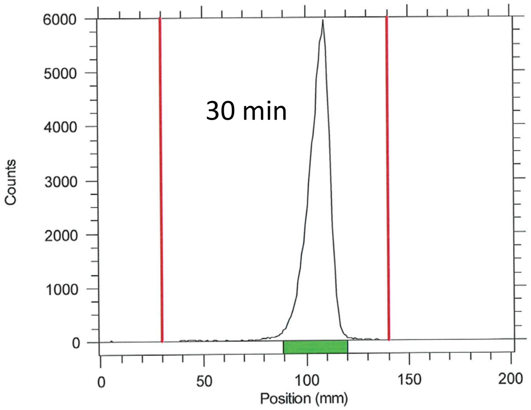

Reg	(mm) Start	(mm) Stop	(mm) Centroid	RF	Region Counts	Region CPM	% of Total	% of ROI
Rgn 1	91.9	120.9	107.8	0.707	43453.0	21196.6	94.83	100.00
1 Peaks					43453.0	21196.6	94.83	100.00

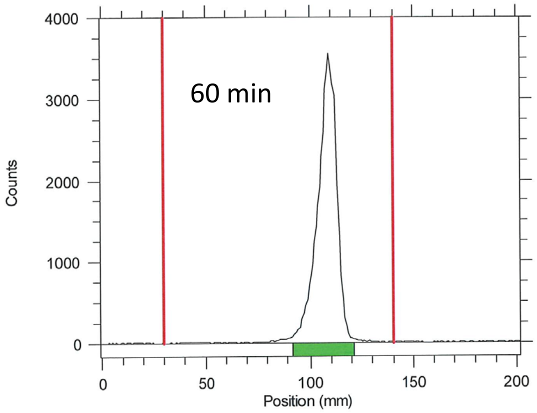

Reg	(mm) Start	(mm) Stop	(mm) Centroid	RF	Region Counts	Region CPM	% of Total	% of ROI
Rgn 1	103.9	128.5	115.8	0.780	36620.0	26472.3	97.15	100.00
1 Peaks					36620.0	26472.3	97.15	100.00

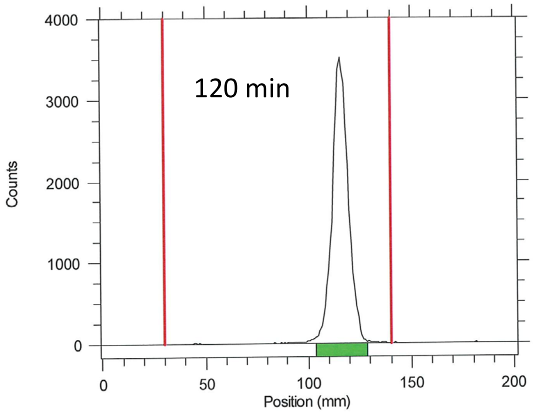

Supplementary Figure S10: Representative radio-TLCs of [^{18}F]**6** stability in 0.9% Saline solution over time.

Reg	(mm) Start	(mm) Stop	(mm) Centroid	RF	Region Counts	Region CPM	% of Total	% of ROI
Rgn 1	94.5	120.9	109.6	0.724	38550.0	42833.3	96.97	100.00
1 Peaks					38550.0	42833.3	96.97	100.00

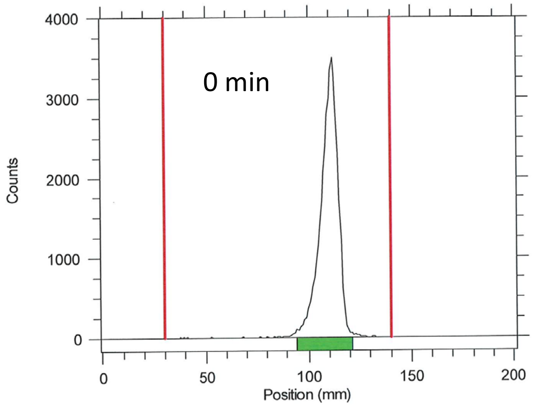

Reg	(mm) Start	(mm) Stop	(mm) Centroid	RF	Region Counts	Region CPM	% of Total	% of ROI
Rgn 1	29.9	46.9	38.0	0.073	5331.0	4569.4	6.17	6.34
Rgn 2	104.7	127.7	116.6	0.787	78814.0	67554.9	91.27	93.66
2 Peaks					84145.0	72124.3	97.45	100.00

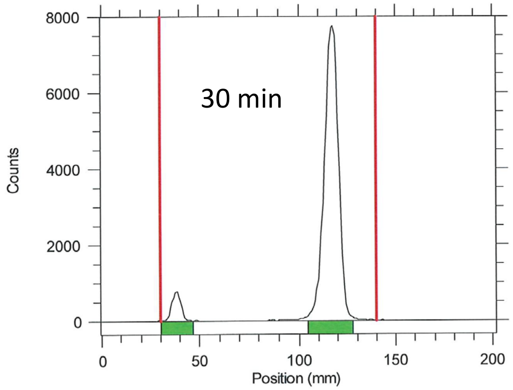

Reg	(mm) Start	(mm) Stop	(mm) Centroid	RF	Region Counts	Region CPM	% of Total	% of ROI
Rgn 1	32.4	46.0	39.0	0.082	9423.0	5489.1	13.77	14.09
Rgn 2	96.2	129.4	112.7	0.752	57456.0	33469.5	83.94	85.91
2 Peaks					66879.0	38958.6	97.71	100.00

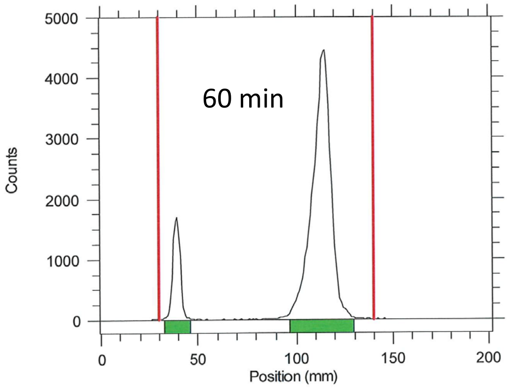

Reg	(mm) Start	(mm) Stop	(mm) Centroid	RF	Region Counts	Region CPM	% of Total	% of ROI
Rgn 1	34.1	50.3	41.9	0.108	15393.0	8396.2	36.61	38.31
Rgn 2	110.7	131.1	120.4	0.822	24790.0	13521.8	58.96	61.69
2 Peaks					40183.0	21918.0	95.57	100.00

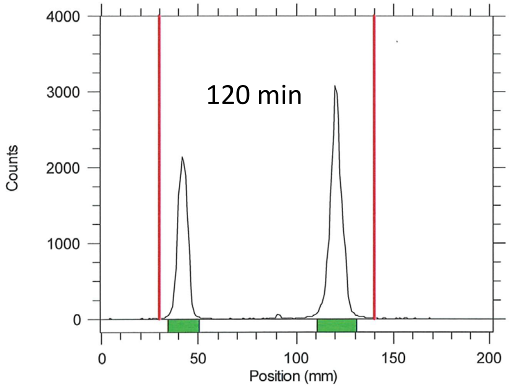

Supplementary Figure S11: Representative radio-TLCs of [^{18}F]**6** stability in mice serum over time.

Reg	(mm) Start	(mm) Stop	(mm) Centroid	RF	Region Counts	Region CPM	% of Total	% of ROI
Rgn 1	85.1	114.9	101.6	0.651	62177.0	63230.8	96.84	100.00
1 Peaks					62177.0	63230.8	96.84	100.00

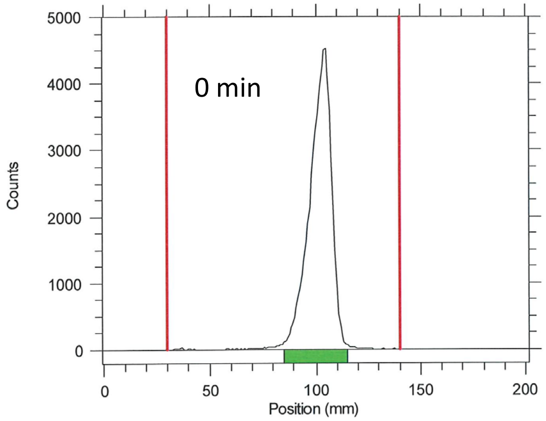

Reg	(mm) Start	(mm) Stop	(mm) Centroid	RF	Region Counts	Region CPM	% of Total	% of ROI
Rgn 1	26.5	44.3	36.0	0.055	3110.0	3217.2	4.46	4.59
Rgn 2	96.2	122.6	110.0	0.728	64692.0	66922.8	92.83	95.41
2 Peaks					67802.0	70140.0	97.30	100.00

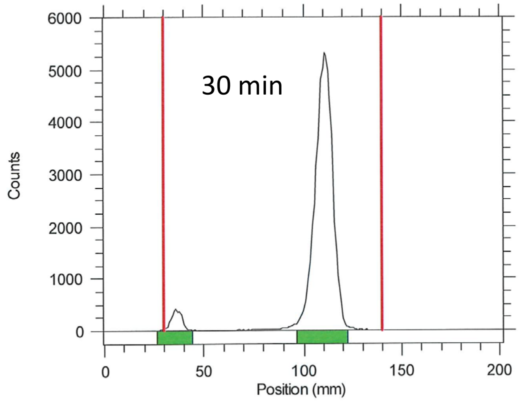

Reg	(mm) Start	(mm) Stop	(mm) Centroid	RF	Region Counts	Region CPM	% of Total	% of ROI
Rgn 1	33.3	47.7	39.7	0.088	6312.0	3104.3	12.09	12.43
Rgn 2	93.6	122.6	109.9	0.726	43192.0	21242.0	82.70	85.04
Rgn 3	127.7	145.5	135.7	0.960	1285.0	632.0	2.46	2.53
3 Peaks					50789.0	24978.2	97.25	100.00

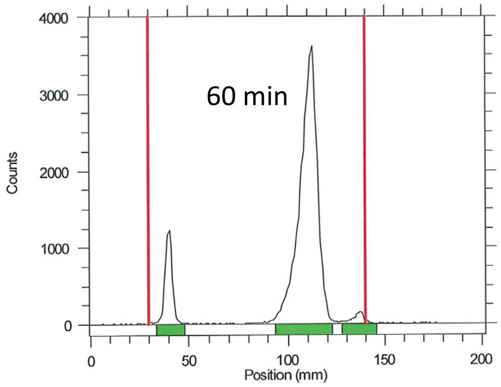

Reg	(mm) Start	(mm) Stop	(mm) Centroid	RF	Region Counts	Region CPM	% of Total	% of ROI
Rgn 1	30.7	45.2	37.2	0.066	9820.0	4120.3	25.64	26.61
Rgn 2	104.7	126.8	115.9	0.781	27082.0	11363.1	70.71	73.39
2 Peaks					36902.0	15483.4	96.34	100.00

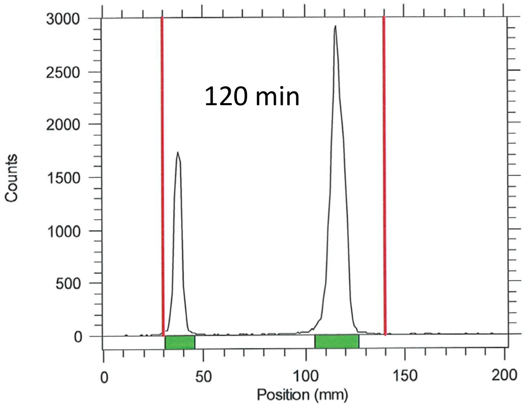

Supplementary Figure S12: Representative radio-TLCs of [^{18}F]**6** stability in human serum over time.

Reg	(mm) Start	(mm) Stop	(mm) Centroid	RF	Region Counts	Region CPM	% of Total	% of ROI
Rgn 1	87.7	111.5	99.7	0.871	170682.0	112537.6	96.53	100.00
1 Peaks					170682.0	112537.6	96.53	100.00

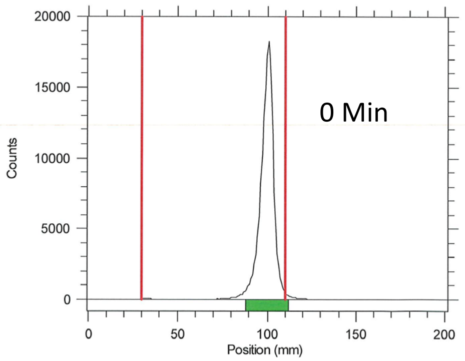

Reg	(mm) Start	(mm) Stop	(mm) Centroid	RF	Region Counts	Region CPM	% of Total	% of ROI
Rgn 1	84.3	108.1	96.3	0.948	173489.0	306157.1	96.07	100.00
1 Peaks					173489.0	306157.1	96.07	100.00

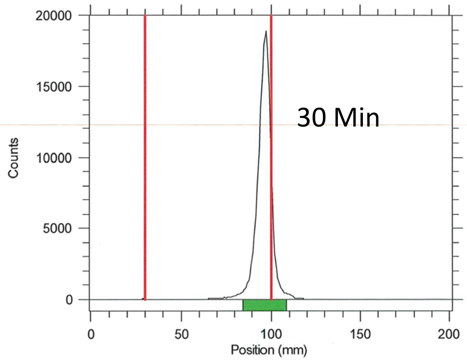

Reg	(mm) Start	(mm) Stop	(mm) Centroid	RF	Region Counts	Region CPM	% of Total	% of ROI
Rgn 1	86.0	117.5	101.7	0.956	169288.0	161226.7	94.97	100.00
1 Peaks					169288.0	161226.7	94.97	100.00

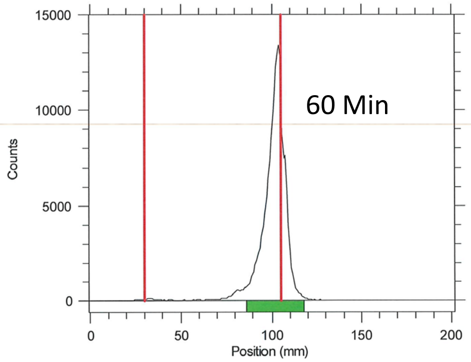

Reg	(mm) Start	(mm) Stop	(mm) Centroid	RF	Region Counts	Region CPM	% of Total	% of ROI
Rgn 1	84.3	108.1	96.3	0.948	173489.0	306157.1	96.07	100.00
1 Peaks					173489.0	306157.1	96.07	100.00

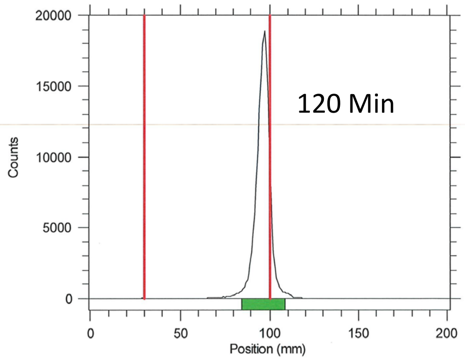

Supplementary Figure S13: Representative radio-TLCs of [^{18}F]**2** stability in 0.9% Saline solution over time.

Reg	(mm) Start	(mm) Stop	(mm) Centroid	RF	Region Counts	Region CPM	% of Total	% of ROI
Rgn 1	87.7	116.6	102.8	1.040	171027.0	155479.1	97.06	100.00
1 Peaks					171027.0	155479.1	97.06	100.00

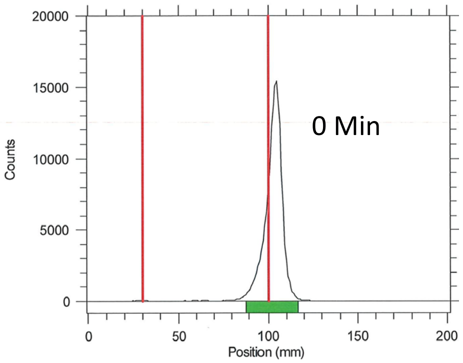

Reg	(mm) Start	(mm) Stop	(mm) Centroid	RF	Region Counts	Region CPM	% of Total	% of ROI
Rgn 1	80.0	115.8	97.8	0.969	174965.0	349930.0	97.35	100.00
1 Peaks					174965.0	349930.0	97.35	100.00

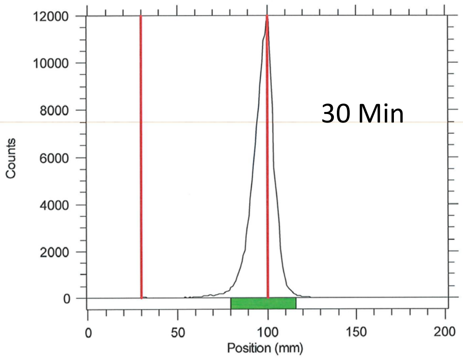

Reg	(mm) Start	(mm) Stop	(mm) Centroid	RF	Region Counts	Region CPM	% of Total	% of ROI
Rgn 1	70.7	107.3	90.0	0.857	175350.0	263025.0	96.69	100.00
1 Peaks					175350.0	263025.0	96.69	100.00

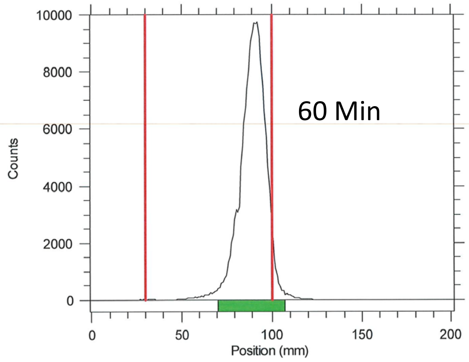

Reg	(mm) Start	(mm) Stop	(mm) Centroid	RF	Region Counts	Region CPM	% of Total	% of ROI
Rgn 1	75.8	108.1	92.5	0.782	169721.0	72221.7	96.10	100.00
1 Peaks					169721.0	72221.7	96.10	100.00

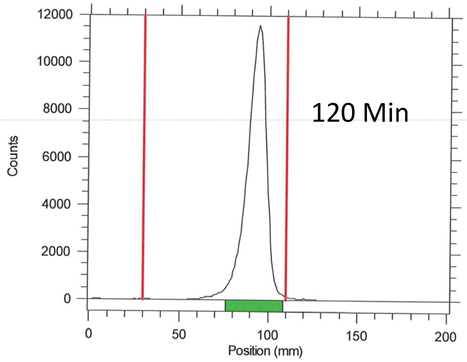

Supplementary Figure S14: Representative radio-TLCs of [^{18}F]**2** stability in mice serum over time.

Reg	(mm) Start	(mm) Stop	(mm) Centroid	RF	Region Counts	Region CPM	% of Total	% of ROI
Rgn 1	77.5	107.3	93.4	0.905	172376.0	313410.9	96.76	100.00
1 Peaks					172376.0	313410.9	96.76	100.00

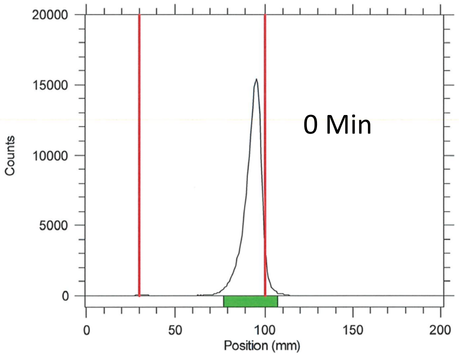

Reg	(mm) Start	(mm) Stop	(mm) Centroid	RF	Region Counts	Region CPM	% of Total	% of ROI
Rgn 1	73.2	104.7	89.6	0.851	169127.0	195146.5	95.57	100.00
1 Peaks					169127.0	195146.5	95.57	100.00

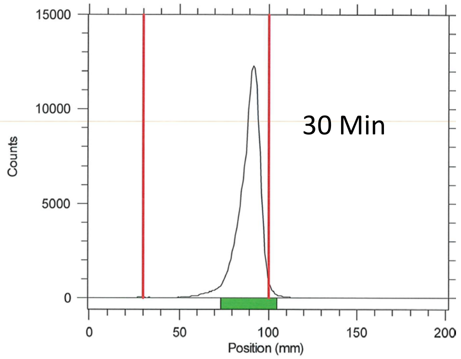

Reg	(mm) Start	(mm) Stop	(mm) Centroid	RF	Region Counts	Region CPM	% of Total	% of ROI
Rgn 1	78.3	112.4	96.0	0.879	175375.0	202355.8	98.07	100.00
1 Peaks					175375.0	202355.8	98.07	100.00

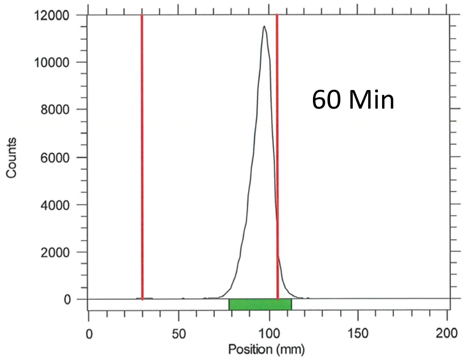

Reg	(mm) Start	(mm) Stop	(mm) Centroid	RF	Region Counts	Region CPM	% of Total	% of ROI
Rgn 1	80.9	112.4	96.6	0.833	168468.0	60527.4	97.00	100.00
1 Peaks					168468.0	60527.4	97.00	100.00

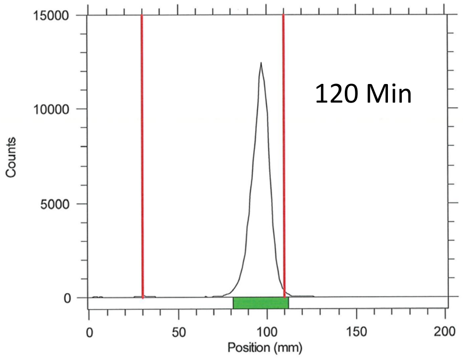

Supplementary Figure S15: Representative radio-TLCs of [^{18}F]**2** stability in human serum over time.

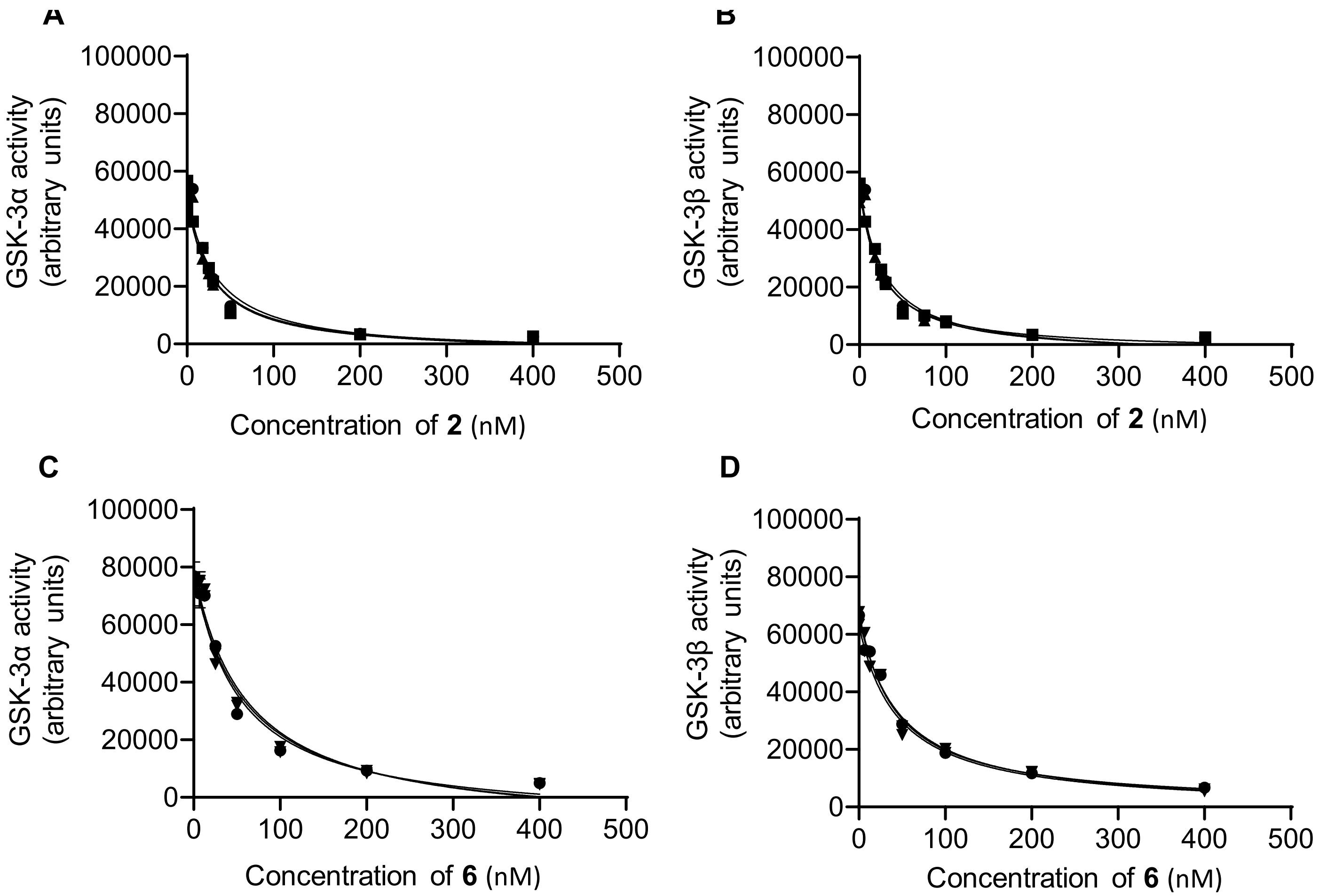

Supplementary Figure S16: Non-linear regression fit curves of (A) glycogen synthase kinase 3 alpha (GSK-3α) vs concentration of **2** (nM), (B) glycogen synthase kinase 3 beta (GSK-3β) vs concentration of **2** (nM), (C) glycogen synthase kinase 3 alpha (GSK-3α) vs concentration of **6** (nM) and (D) glycogen synthase kinase 3 beta (GSK-3β) *vs* concentration of **6** (nM).

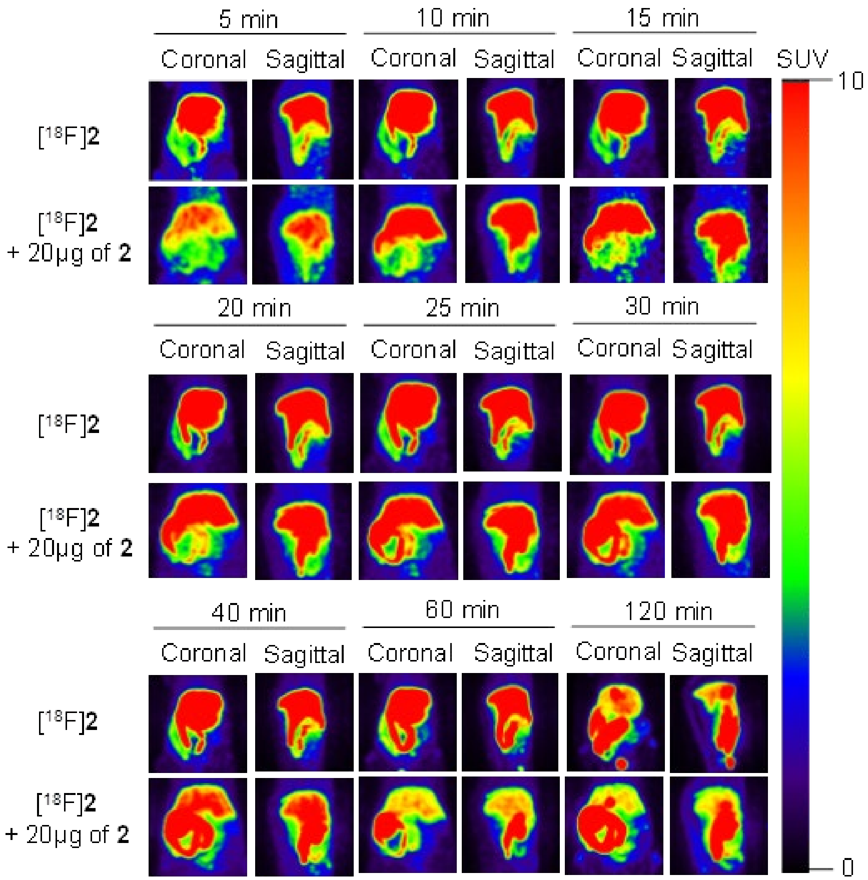

Supplementary Figure S17: Representative PET images (coronal and sagittal section) showing uptake of [^{18}F]**2** and [^{18}F]**2** with 20µg of **2** in liver of FVB/NJ mice at different time points post-injection.

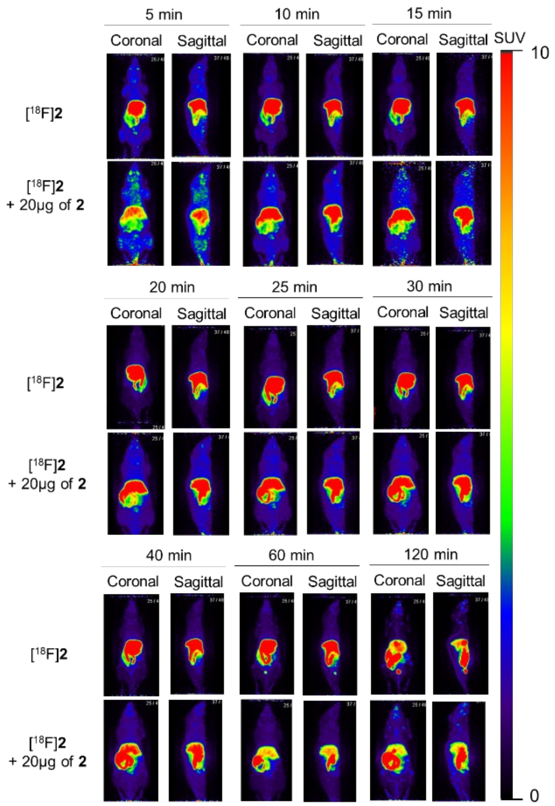

Supplementary Figure S18: Representative PET images showing uptake of [^{18}F]**2** and [^{18}F]**2** with 20µg of **2** in whole body of FVB/NJ mice with major uptake in liver at different time points post-injection.

	Brain		Liver	
Time	[^{18}F]**2** (n=6)	[^{18}F]**2** +20µg of **2** (n=6)	[^{18}F]**2** (n=6)	[^{18}F]**2** +20µg of **2** (n=6)
5 min	0.92 ± 0.28	0.93 ± 0.06	5.66 ± 1.18	5.24 ± 1.46
10 min	0.38 ± 0.06	0.57 ± 1.06*	7.43 ± 0.66	5.86 ± 0.51*
15 min	0.24 ± 0.03	0.50 ± 0.06*	7.89 ± 0.59	5.49 ± 0.28*
20 min	0.21 ± 0.02	0.43 ± 0.06*	7.88 ± 0.60	5.19 ± 0.27*
25 min	0.19 ± 0.03	0.40 ± 0.06*	7.61 ± 0.65	4.93 ± 0.30*
30 min	0.19 ± 0.04	0.36 ± 0.06*	7.34 ± 0.77	4.76 ± 0.33*
40 min	0.18 ± 0.04	0.31 ± 0.06*	6.91 ± 0.91	4.55 ± 0.23*
50 min	0.17 ± 0.03	0.28 ± 0.06*	6.40 ± 1.04	4.31 ± 1.15*
60 min	0.16 ± 0.03	0.26 ± 0.06*	5.95 ± 1.12	4.09 ± 1.16*
120 min	0.09 ± 0.01	0.18 ± 0.01*	2.78 ± 1.40	3.19 ± 0.37

Supplementary Table S2. Uptake of [^{18}F]**2** and [^{18}F]**2** with **2** in brain and liver of FVB/NJ mice at different time points post-injection assessed by image analysis. Data expressed as standardized uptake value (SUV). The SUVs were calculated by image analysis and each data point is average ± standard deviation. *$P < 0.05$ [^{18}F]**2** vs [^{18}F]**2** + 20µg of **2**

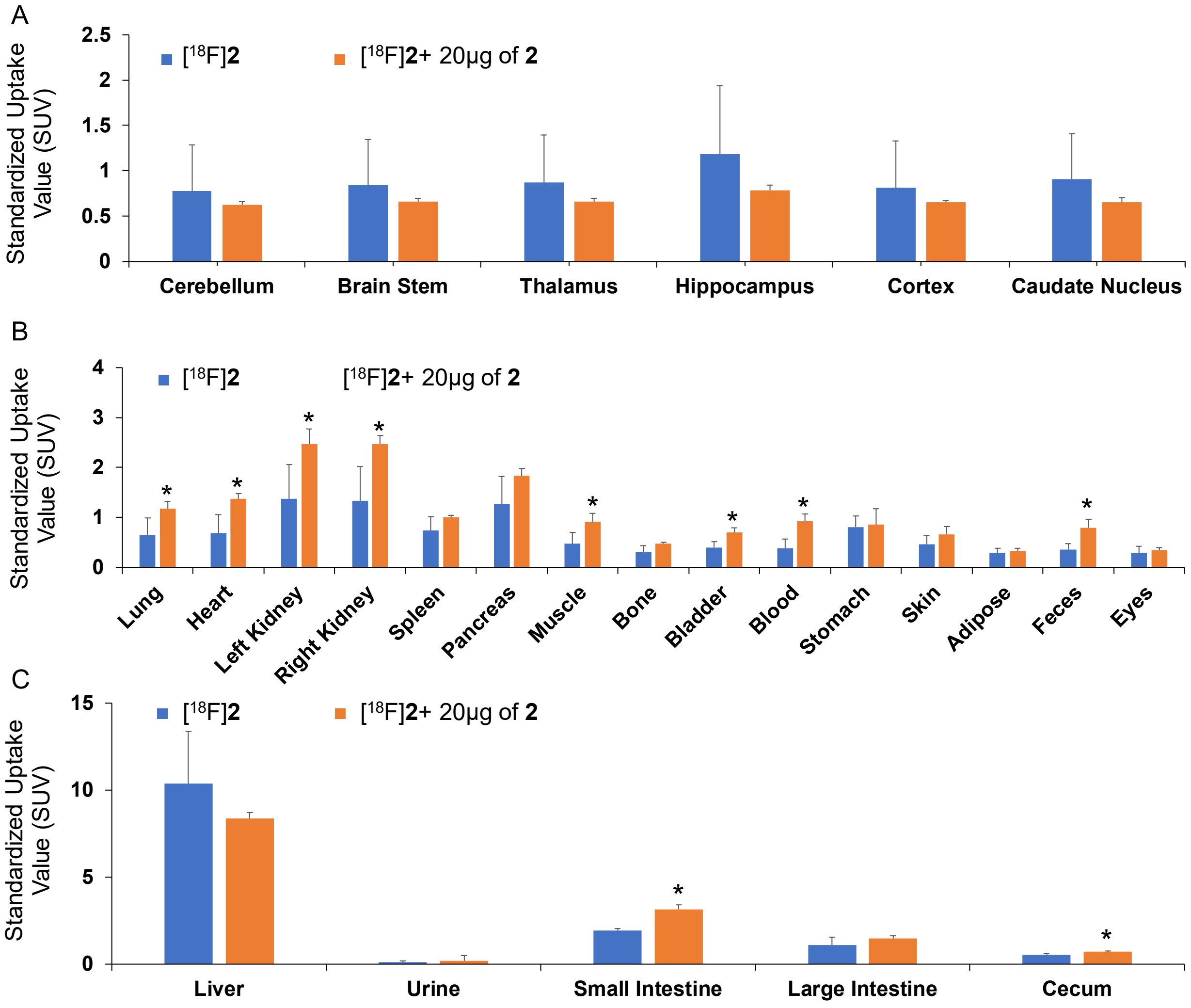

Supplementary Figure S19. Uptake of [^{18}F]**2** (n=4) and [^{18}F]**2** with 20μg of **2** (n=3) in (A) brain and (B and C) other major organs in FVB/NJ mice at 5 min post intravenous (i.v.) administration. *P < 0.05 [^{18}F]**2** vs [^{18}F]**2** + 20μg of **2**.

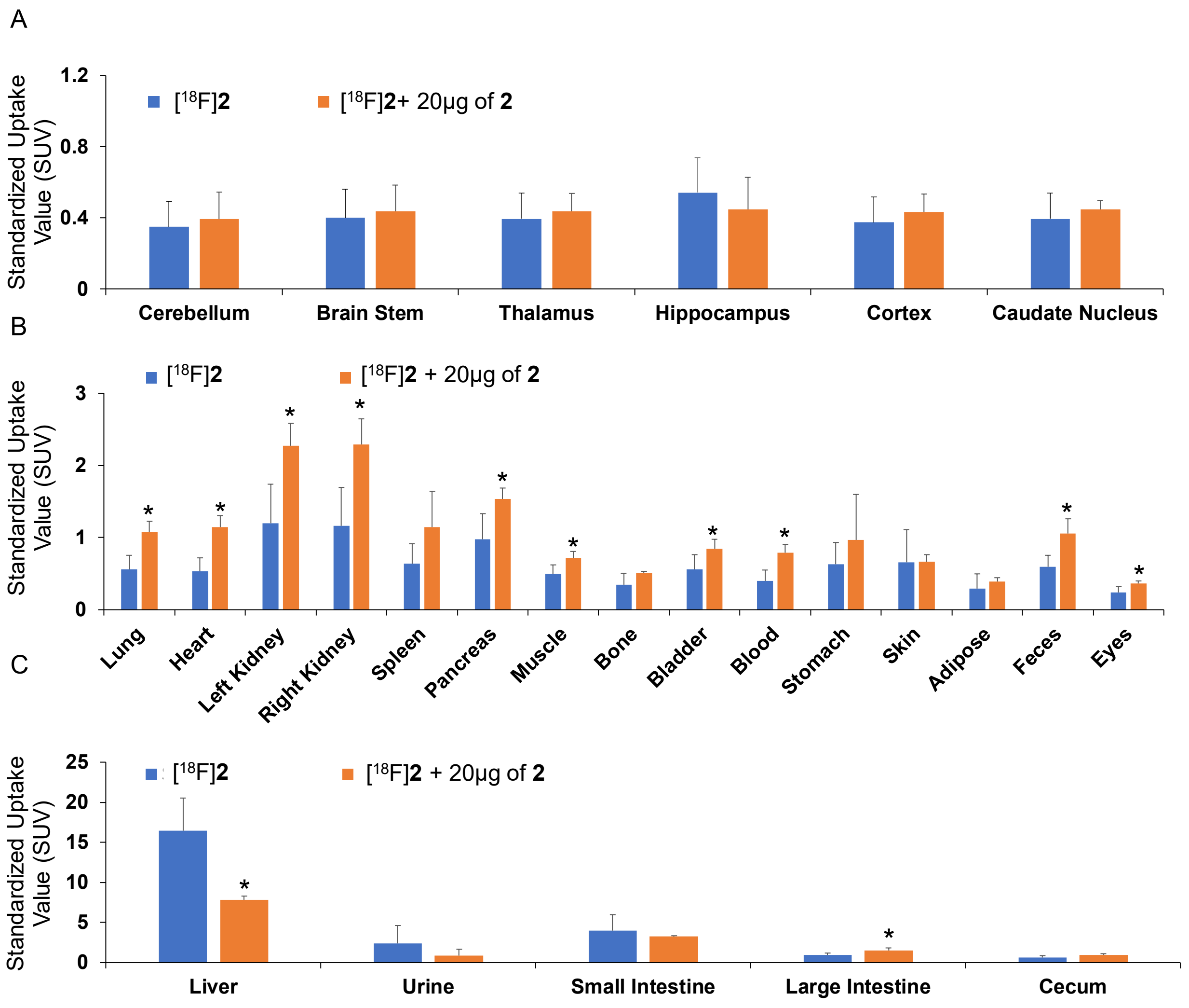

Supplementary Figure S20. Uptake of [^{18}F]**2** (n=4) and [^{18}F]**2** with 20µg of **7** (n=3) in (A) brain and (B and C) other major organs in normal FVB/NJ mice at 10 min post intravenous (i.v.) administration. *P < 0.05 [^{18}F]**2** vs [^{18}F]**2** + 20µg of **2**.

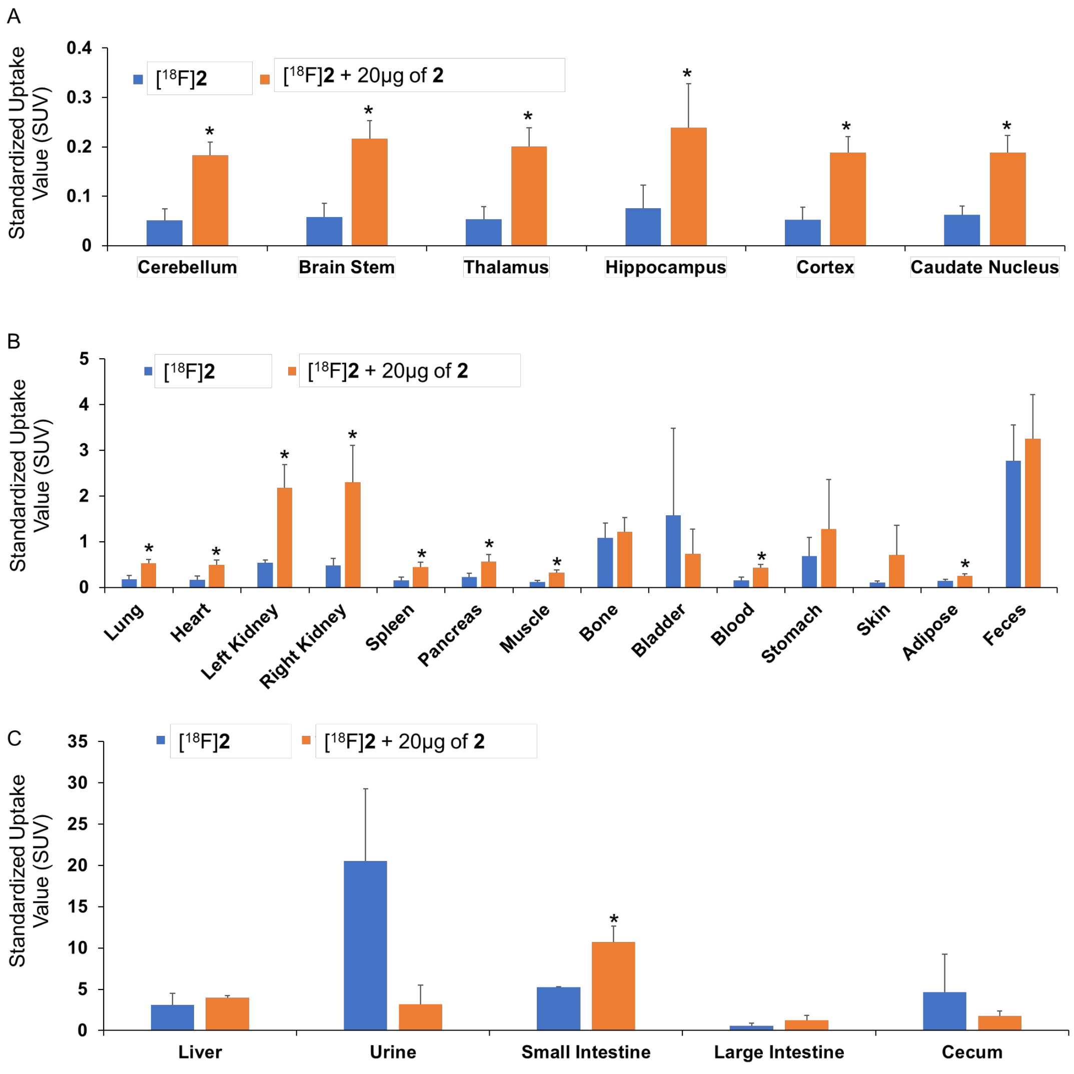

Supplementary Figure S21. Uptake of [^{18}F]**2** (n=3) and [^{18}F]**2** with 20μg of **2** (n=3) in (A) brain and (B and C) other major organs in normal FVB/NJ mice at 120 min post intravenous (i.v.) administration. *P < 0.05 [^{18}F]**2** vs [^{18}F]**2** + 20μg of **2**.
